# Sublingual immunotherapy: World Allergy Organization position paper 2013 update

**DOI:** 10.1186/1939-4551-7-6

**Published:** 2014-03-28

**Authors:** Giorgio Walter Canonica, Linda Cox, Ruby Pawankar, Carlos E Baena-Cagnani, Michael Blaiss, Sergio Bonini, Jean Bousquet, Moises Calderón, Enrico Compalati, Stephen R Durham, Roy Gerth van Wijk, Désirée Larenas-Linnemann, Harold Nelson, Giovanni Passalacqua, Oliver Pfaar, Nelson Rosário, Dermot Ryan, Lanny Rosenwasser, Peter Schmid-Grendelmeier, Gianenrico Senna, Erkka Valovirta, Hugo Van Bever, Pakit Vichyanond, Ulrich Wahn, Osman Yusuf

**Affiliations:** 1 Respiratory and Allergy Clinic, DIMI—Department of Internal Medicine, University of Genoa, IRCCS Aou San Martino, Largo Rosanna Benzi 10, Genoa 1-16132, Italy; 2 Department of Medicine, Nova Southeastern University, College of Osteopathic Medicine, Davie Florida, USA; 3 Division of Allergy, Department of Pediatrics, Nippon Medical School, Tokyo, Japan; 4 Research Center for Respiratory Medicine (CIMER), Catholic University, Fundación LIBRA, Córdoba, Argentina; 5 Department of Pediatrics and Medicine, University of Tennessee Health Science Center, Memphis, Tennessee, USA; 6 Department of Medicine, Second University of Naples, Institute of Translational Pharmacology, Italian National Research Council, Rome, Italy; 7 Centre Hospitalier Regional Universitaire de Montpellier, Université de Montpellier, Montpellier, France; 8 Section of Allergy and Clinical Immunology, Imperial College of London, National Heart and Lung Institute, Royal Brompton Hospital, London, UK; 9 Allergy and Respiratory Diseases Clinic, Department of Internal Medicine, University of Genoa, Genova, Italy; 10 Allergy and Clinical Immunology, National Heart and Lung Institute, Imperial College of London, London, UK; 11 Department of Allergology, Erasmus MC, University Medical Center, Rotterdam, The Netherlands; 12 Allergy Department, Hospital Medica Sur, Mexico City, Mexico; 13 National Jewish Health, University of Colorado – Denver School of Medicine, Denver, Colorado, USA; 14 Allergy and Respiratory Diseases, IRCCS San Martino IST, University of Genoa, Genova, Italy; 15 Center for Rhinology and Allergology Wiesbaden, Department of Otorhinolaryngology, Head and Neck Surgery, University Hospital Mannheim, Mannheim, Germany; 16 Pediatric Allergy and Immunology Division, Hospital de Clínicas, Federal University of Parana, Curitiba, Brazil; 17 Academic Centre of Primary Care, Division of Applied Health Sciences, University of Aberdeen, Aberdeen, UK; 18 Children’s Mercy Hospital, University of Missouri – Kansas City School of Medicine, Kansas City, Missouri; 19 Allergy Unit, Department of Dermatology, University Hospital of Zürich, Zürich, Switzerland; 20 Allergy Unit, Verona General Hospital, Verona, Italy; 21 Department of Paediatrics, University Children’s Medical Institute, Yong Loo Lin School of Medicine, National University of Singapore, Singapore, Singapore; 22 Department of Clinical Allergology and Pulmonary Diseases, University of Turku, Finland, and Allergy Clinic, Terveystalo, Turku, Finland; 23 Department of Pediatrics, Faculty of Medicine, Siriraj Hospital, Mahidol University, Bangkok, Thailand; 24 Department of Pediatric Pneumology and Immunology, Charité, Humboldt University, Berlin, Germany; 25 The Allergy and Asthma Institute, Islamabad, Pakistan

**Keywords:** Sublingual immunotherapy, Allergen-specific immunotherapy, Mechanisms of SLIT, Safety of SLIT, Efficacy of SLIT, Clinical trials methodology in SLIT

## Abstract

We have prepared this document, “Sublingual Immunotherapy: World Allergy Organization Position Paper 2013 Update”, according to the evidence-based criteria, revising and updating chapters of the originally published paper, “Sublingual Immunotherapy: World Allergy Organization Position Paper 2009”, available at http://www.waojournal.org. Namely, these comprise: “Mechanisms of sublingual immunotherapy;” “Clinical efficacy of sublingual immunotherapy” – reporting all the data of all controlled trials published after 2009; “Safety of sublingual immunotherapy” – with the recently published Grading System for adverse reactions; “Impact of sublingual immunotherapy on the natural history of respiratory allergy” – with the relevant evidences published since 2009; “Efficacy of SLIT in children” – with detailed analysis of all the studies; “Definition of SLIT patient selection” – reporting the criteria for eligibility to sublingual immunotherapy; “The future of immunotherapy in the community care setting”; “Methodology of clinical trials according to the current scientific and regulatory standards”; and “Guideline development: from evidence-based medicine to patients' views” – including the evolution of the methods to make clinical recommendations.

Additionally, we have added new chapters to cover a few emerging crucial topics: “Practical aspects of schedules and dosages and counseling for adherence” – which is crucial in clinical practice for all treatments; “Perspectives and new approaches” – including recombinant allergens, adjuvants, modified allergens, and the concept of validity of the single products. Furthermore, “Raising public awareness about sublingual immunotherapy”, as a need for our patients, and strategies to increase awareness of allergen immunotherapy (AIT) among patients, the medical community, all healthcare stakeholders, and public opinion, are also reported in detail.

## Foreword

### An introductory address by Professor Guido Rasi, Executive Director, European Medicines Agency (EMA)

Immunotherapy and Biologics represent some of the most important hot topics in the field of medicine. In fact, not only is there a tremendous increase in the requests for scientific advice and marketing authorization for biologics and immunological treatments to the European Medicines Agency (EMA), but these therapies call for new methods of research, strategies for development, evaluation, use and pharmacovigilance.

The allergen products used for both the diagnosis and treatment of allergic diseases require particular attention among the immunological and biological therapeutic areas, due to the increase in the prevalence and social relevance of allergies as well as the regulatory actions being requested by the Directive 2001/83/EC and the following amending EC Directives.

In order to discuss the available EMA Guidelines on Allergen Products and to address the many issues that need harmonizing, and the regulatory aspects of allergen products in Europe, the EMA on February 28, 2013 called a workshop in London with the participation of all stakeholders.

The WAO SLIT document presented in this issue of the World Allergy Organization Journal, and the EAACI “Task Force Report on Clinical Outcomes to be used in studies of Allergen Products”, represent relevant contributions to answer the recommendations emerging from this workshop.

From our side, we are working on a document, to be shared with all stakeholders, on possible pathways for facilitating marketing authorization of allergen products. These might include:

▪ a wider utilization of collaborative trials, with the advantage of using the same control group for studies of comparable active products, a substantial advantage for pediatric investigational plans;

▪ new facilitated pathways of marketing authorization allowing a faster access for patients to immunotherapy without affecting an evidence-based risk/benefit ratio;

▪ new study designs for well-conducted post-registration trials, which, with the help of registries, will allow a progressive evaluation of efficacy and safety of new products and a pharmacovigilance in line with the new ad hoc law;

▪ adequate information technology to take full advantage of a wider access to individual data of registration trials.

The harmonization of regulatory aspects of allergen products represents however a work still in progress, as shown by the concordant position on efficacy but the different end-points adopted as well by the different safety considerations in the recent FDA Advisory Committee votes on two SLIT products already approved by EMA and marketed in most European Countries.

Therefore, we expect that the regulatory bodies, scientific societies, manufacturers of allergen products, and allergy patients’ organizations will continue their joint commitment with the common goal of providing worldwide to the many millions of subjects suffering from allergic diseases an early and harmonized access to safe and effective products.

## Chapter 1 Introduction and historical background to sublingual immunotherapy

**Figure 1:**
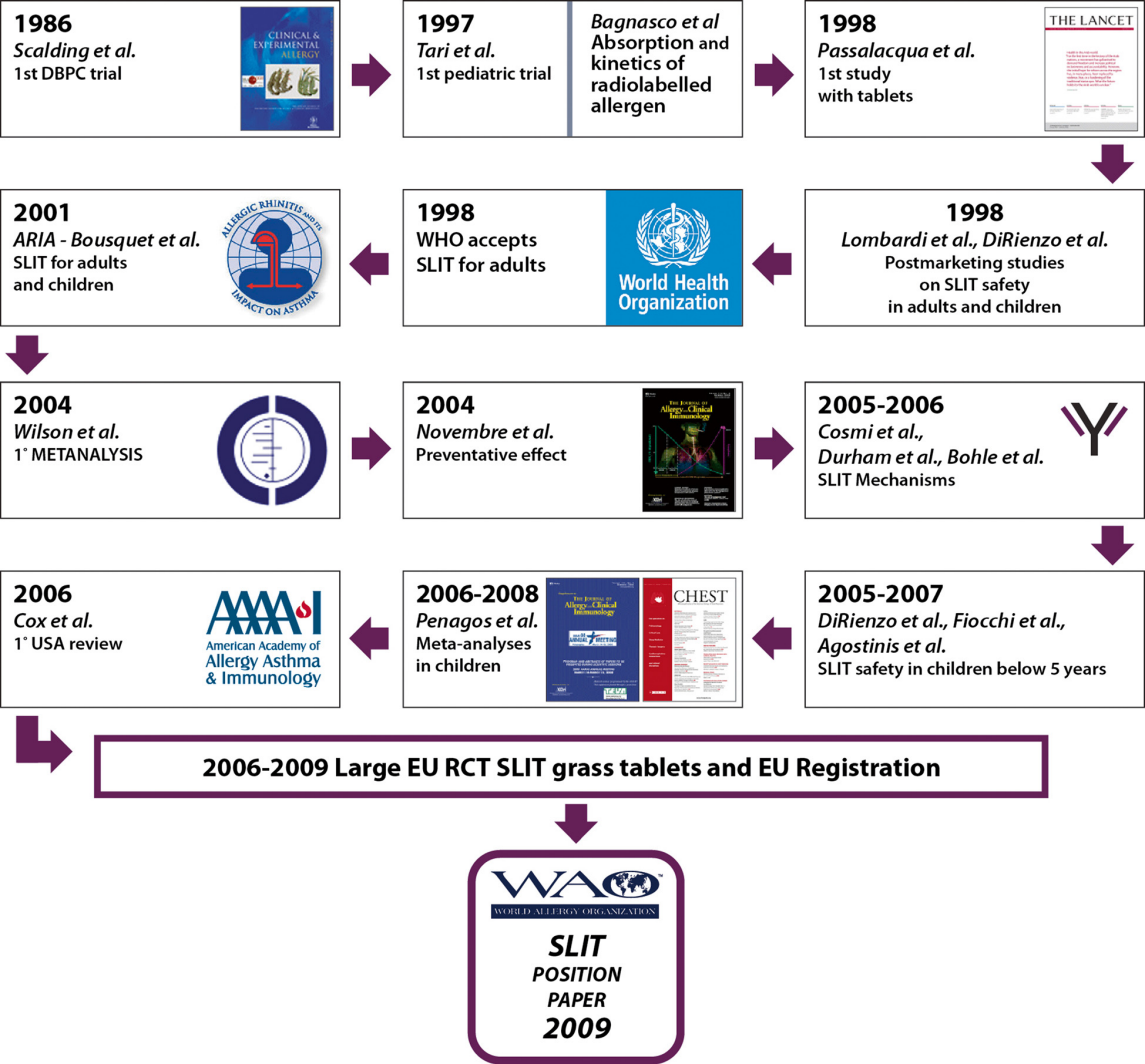
The historical process leading to the WAO SLIT Position Paper 2009.

The update of chapter one consists of two figures. Figure 1 provides an overview of the history of the development of the first position paper, which was published in 2009.

**Figure 2:**
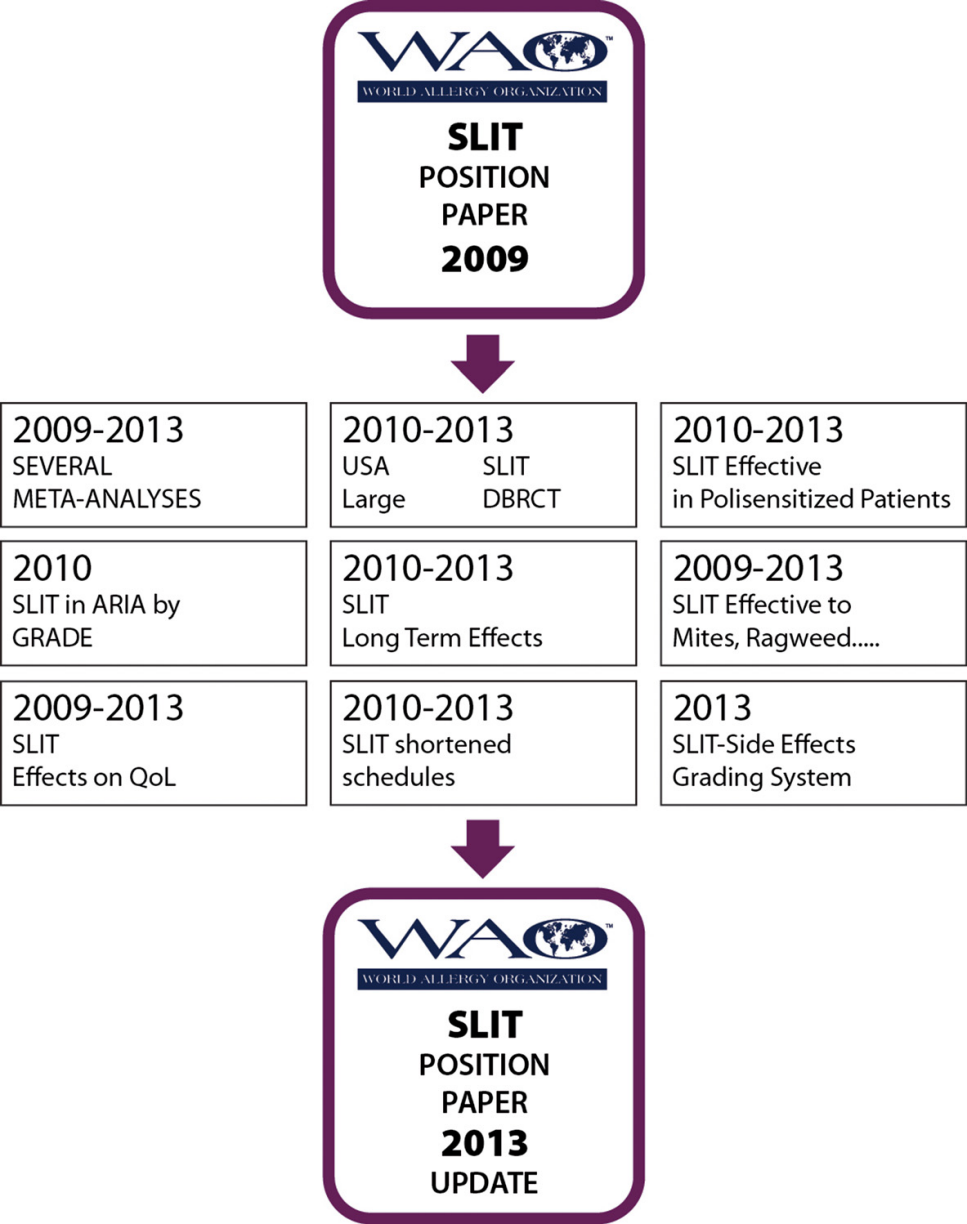
Highlights of the evidences in SLIT, from 2009 to 2013, the scientific basis for the updating of “Sublingual Immunotherapy: World Allergy Organization Position Paper 2009”.

The next figure displays the evidence base for sublingual immunotherapy since the publication of the first position paper (Figure 2).

## Chapter 2 Allergen-specific immunotherapy

There were no changes indicated for this chapter, and related updated details were integrated into other chapters.

## Chapter 3 Mechanisms of sublingual immunotherapy

• Allergen immunotherapy provides an opportunity to study antigen-specific tolerance in humans.

• Subcutaneous immunotherapy (SCIT) suppresses allergic Th2-mediated inflammation and increases antigen-specific IgG, probably by induction of regulatory T cells (Tregs), immune deviation (Th2 to Th1), and/or apoptosis of effector memory Th2 cells.

• The oral mucosa is a natural site of immune tolerance (Langerhans cells, FcR1, IL-10, IDO [indoleamine 2,3-dioxygenase]).

• Sublingual immunotherapy (SLIT) in optimal doses is effective; SLIT has been shown to induce long-term remission after discontinuation and may prevent new sensitizations, features consistent with the induction of tolerance.

• SLIT induces modest systemic changes consistent with SCIT, but additional local mechanisms in the oral mucosa and/or regional lymph nodes are likely important.

• Sublingual immunotherapy is associated with

○ retention of allergen in sublingual mucosa for several hours.

○ early increases in antigen-specific IgE and blunting of seasonal IgE.

○ persistent increases in antigen-specific IgG4 and IgE blocking activity that parallel long-term clinical benefits of both SCIT and SLIT.

○ inhibition of eosinophils and reduction of adhesion molecules in target organs.

○ an early (at 4-12 weeks) increase in peripheral phenotypic Tregs and delayed (at 12 months) immune deviation in favor of Th1 responses.

○ detection of CD25 + FOXP + phenotypic Treg cells in the sublingual mucosa.

○ alterations in dendritic cell markers (e.g., increases in expression of complement component C1Q) that correlate with clinical response to treatment and merit further study.

• Biomarkers that are predictive of or surrogates for the clinical response to immunotherapy are not currently available for routine use.

○ Molecular diagnosis of IgE sensitivities will aid patient selection for immunotherapy.

○ Serum IgG–associated functional blocking activity and basophil activation tests merit further study.

○ Studies of peripheral T cell and dendritic cell signatures have yielded important information, but these tests are currently impractical for routine clinical use.

### Clinical evidence for disease modification and induction of tolerance

Since 2009, Cochrane meta-analyses have confirmed the efficacy and safety of SLIT for seasonal and perennial allergic rhinitis [1] and conjunctivitis [2]. Several large ‘definitive’ trials have now confirmed the efficacy and safety for seasonal rhinitis in both children and adults. Long-term benefits of SLIT for at least 1 [3] or 2 [4] years following discontinuation of treatment have been demonstrated in 2 large independent trials of immunotherapy with grass pollen allergen tablets in adults. These studies provide evidence for long-term disease remission and disease modification consistent with the induction of antigen-specific tolerance. In parallel with these novel clinical data, there have been advances in our understanding of the underlying mechanisms of SLIT that may give rise to putative biomarkers to predict the clinical response [5-8].

### The oropharyngeal mucosa as a tolerogenic organ

The oral cavity is a naturally tolerogenic environment, remaining noninflamed despite being exposed continuously to multiple foreign proteins. The presence of Langerhans cells and monocytes capable of producing IL-10 and TGF-β are major contributors to the maintenance of tolerance (see Chapter 3 of the 2009 WAO position paper [9]). A recent study [10] has shown that T cells isolated from the human buccal mucosa, in contrast to those isolated from skin, express TGF-β1, IL-10, interferon-γ and IL-17, particularly in the vestibular region, and markedly express toll-like receptor (TLR) 2 and TLR4. Another study from the same group [11] confirmed that oral Langerhans cells within fresh human oral mucosal biopsies are capable of rapidly taking up Phl p 5 (within 5 minutes) in a dose-dependent fashion, resulting in their attenuated maturation and enhanced production of inhibitory cytokines (IL-10 and TGF-β). Taken together with the paucity of mast cells in the oral vestibule compared to the sublingual region [12], these data raise the possibility that targeting the vestibule with allergen vaccine with or without adjuvant has the potential to induce enhanced immune deviation or tolerance, possibly with a lower potential for mast cell–related local side effects, although this remains to be tested.

Palomares et al. [13] recently highlighted that the tonsils and surrounding lymphoid tissue are located in the gateway of both the alimentary and respiratory tracts and may be important for local induction of tolerance to both food and inhalant allergens. Abundant FOXP3^+^ Treg cells were detected in lingual and palatine tonsils. Tonsil-derived plasmacytoid dendritic cells (DCs) were shown to have the ability to generate functional CD4^+^ CD25^+^ CD127^–^ FOXP3^+^ Treg cells. Triggering of tonsil-derived T cells with TLR4 or TLR8 agonists or the proinflammatory cytokines IL-1β or IL-6 was able to break allergen-specific T-cell tolerance through a mechanism dependent on the adaptor molecule myeloid differentiation primary response gene 88 (MyD88) [14]. In particular, myeloid DCs and stimuli that activate them broke the tolerance of allergen-specific CD4^+^ T cells, whereas plasmacytoid DCs and stimuli that activate them, such as TLR7 and TLR9 agonists, did not have any effect. These human *ex vivo* data raise the possibility that immunotherapy strategies that target tonsillar tissue may enhance the induction of tolerance, but this remains to be tested in the context of different strategies of oral immunotherapy.

In a double-blind 18-month controlled trial of high-dose grass pollen SLIT (20 mcg Phl p 5 daily), biopsies of the sublingual mucosa demonstrated more CD3^+^ FOXP3^+^ and CD25^+^ FOXP3^+^ T cells in SLIT-treated patients than in placebo-treated patients, and some CD3^+^ FOXP3^+^ cells were shown with triple immunofluorescence to express IL-10, a direct illustration of the induction of local phenotypic Treg cells following successful treatment [15].

### Specific antibody levels

Studies of sublingual grass pollen immunotherapy in general show an increase in serum allergen-specific IgG4 and IgG, although the increase is not as great as that seen with SCIT [16,17]. Transient early increases in allergen-specific IgE have also been observed and are associated with blunting of seasonal increases in IgE [4]. However, some SLIT studies have not shown increases in IgG levels, particularly following house dust mite immunotherapy [18]. In a study of high-dose grass pollen SLIT, increases in grass pollen–specific IgA2 as well as IgG and IgG4 occurred in parallel with local increases in sublingual FOXP3^+^ Tregs [15]. These increases were accompanied by increases in serum inhibitory activity for binding of allergen-IgE complexes to B cells (IgE-FAB), a validated surrogate of IgE-facilitated antigen presentation to T cells. Furthermore, the long-term clinical benefit observed for 2 years following a 3-year course of grass pollen SLIT was associated with persistent elevations in serum levels of both allergen-specific IgG4 [3,4] and functional IgG-associated inhibitory activity for IgE-FAB when compared to the levels in placebo-treated patients [4].

### Effector cells in the target organ

Eosinophils and inflammatory mediators are elevated in nasal lavage fluid and nasal biopsies after nasal allergen challenge and during seasonal pollen exposure [Chapter 3 of ref. 9]. Recent attempts have been made to standardize the collection of cytokines, mediators, and antibodies in fluid collected on filters and sponges applied directly to the nasal mucosa after nasal challenge [19]. For example, tryptase levels peak at 5 minutes, consistent with early mast cell activation, whereas increases in eosinophil cationic protein (ECP), a marker of eosinophils and Th2 cytokines (IL-4, IL-5, and IL-13), peak at 3 to 8 hours during the late nasal response. Whether alterations in these local mediators, antibodies, and cytokines correlate with the clinical response to immunotherapy remains to be tested.

### Effector cells in the periphery

Basophil activation can be measured *ex vivo* by short-term (1 h) allergen stimulation of freshly harvested whole blood. Techniques include measurement of basophil histamine release and expression of surface activation markers, including CD63 and CD203b. Inhibition of basophil activation has been shown following SLIT with a combined grass and mite extract [20]; however, another study of grass pollen SLIT [21] found no changes in basophil activation and no correlation with clinical response to treatment.

### T cells and dendritic cells

Studies of peripheral blood T cell phenotype, proliferation, and cytokine production following SLIT have given variable results. This is in part due to the different allergens, doses, and protocols employed for immunotherapy and the varying methods of T cell purification, processing, and storage, that are difficult to standardize in multicenter studies. Evaluation of T cell phenotype and function overall favor the induction of Tregs and/or immune deviation in favor of Th1 cells. Thus, birch pollen SLIT in one study resulted in the induction of CD25^+^ FOXP3^+^ T cells at 4 weeks [22]. CD25^+^ Tregs suppressed antigen-stimulated CD25^–^ T cell proliferation. This suppression was reversible at 4 weeks but not at 12 months by the addition of an anti-IL-10 antibody to the culture medium. In a study of house dust mite SLIT, suppression of antigen-stimulated purified CD4^+^ T cell proliferation was reversible at 6 but not 12 months by treatment with soluble TGF-β receptor [18]. These data support that Tregs are induced early, followed by delayed immune deviation in favor of Th1 responses at 12 months. Several other recent studies support these observations [23-27].

In a double-blind study [20] of daily SLIT with the combination of house dust mite and timothy grass pollen in dual-sensitized adults (n = 30), clinical improvement and decreased nasal allergen sensitivity were accompanied by an increase in allergen-stimulated CD4^+^ CD25^+^ CD127^low^ CD45RO^+^ Foxp3^+^ T cells with reduced DNA methylation at CpG sites [20], implying that tolerance might result from epigenetic modification of memory Treg cells at the Foxp3 locus. In contrast, a double-blind placebo-controlled study of 4 months of grass pollen SLIT (n = 89) that examined T cell phenotype and antigen-specific T cells [28] found that clinical improvement was accompanied by only minor changes in the proportions of CD4^+^ T cells expressing markers for Th1 (CCR5^+^, CXCR3^+^), Th2 (CRTh2^+^, CCR4^+^), or Treg (CD25^+^, CD127^–^, Foxp3^+^). A modest downregulation of IL-4 and IL-10 gene expression and IL-10 secretion (*P* < 0.001) and a decrease in the frequency of potential “pro-allergic” CD27^–^ Th2 cells did not correlate with clinical benefit. Class II-tetramer analyses of antigen-specific T cells failed to show any major impact on either numbers or polarization of circulating CD4^+^ T cells specific for Phl p 1 or Phl p 5. Further and more prolonged studies are required to confirm or exclude whether tetramer tracking of T cells is valuable as a biomarker of early onset of SLIT efficacy.

Tetramer studies of antigen-specific cells are limited by class II restriction and the need to identify relevant T cell epitopes. For this reason, Wambre and colleagues have used T cell surface/intracellular marker expression with or without associated tetramer staining to characterize subjects allergic to grass pollen and mites, with the aim of distinguishing antigen-specific T cells from bystander T cell responses [29]. These and other studies [30] have identified CD154 (CD40 ligand) as a potentially useful T cell marker for future immunotherapy studies.

Human effector and regulatory dendritic cells are important in directing T cell differentiation, phenotype, and function. Following a detailed evaluation *in vitro* of human effector and regulatory dendritic cells from human monocytes cultured under deforming conditions, candidate dendritic cell markers were evaluated at the messenger RNA and protein levels before and after grass pollen allergen tablet SLIT [31]. Complement component 1 (C1Q) and Stabilin-1 (STAB1) were increased in PBMCs from clinical responders in contrast to that seen in nonresponders or placebo-treated patients. Further evaluation of C1Q and STAB1 expression as candidate biomarkers during large trials of sublingual allergen immunotherapy are justified.

### Biomarkers of clinical response to immunotherapy

A useful biomarker is one that is either predictive of or a surrogate for the clinical response to immunotherapy. A predictive biomarker might aid in selecting patients who will respond (or in excluding potential nonresponders), whereas a surrogate biomarker might be effective in monitoring the clinical response to treatment. Ideally, the biomarker should be practical, easy to measure, technically robust, generalizable, and have good sensitivity and specificity. Skin prick testing and raised serum allergen-specific IgE are essential predictive biomarkers that clearly augment information obtained from the history alone. Nonetheless, these tests produce both false positive and false negative results. One study suggested that the ratio of specific IgE to total IgE might relate to the response to immunotherapy [32], whereas an earlier study suggested that the ratio of allergen-specific IgG4 to IgG1 may be more informative [33]. These hypotheses deserve further evaluation. The advent of molecular diagnosis may enhance predictive value by identifying IgE sensitivity to relevant major allergens [34], for example to Phl p 5 and Phl p 1 for grass allergy, rather than only cross-reactive IgE to panallergens such as Phl p 4 or Phl p 12 [35]. When there is discordance between the history and IgE testing, local provocation to the relevant target organ (eye, nose, bronchi) could be helpful, as might the presence of local IgE in nasal, lacrimal, or bronchoalveolar lavage fluid, although whether provocation testing or local IgE is predictive of response to immunotherapy has not been tested.

Based on knowledge of the mechanisms of SLIT, it is attractive to consider that alterations in peripheral T cell or dendritic cell signatures [31] would be useful for monitoring responses, although the complexity of processing and performing these assays makes them unlikely candidates for routine clinical use. Basophil activation testing is of potential value, although conflicting results have been obtained with SLIT [20,21]. Basophil testing requires fresh processing of whole blood and access to a flow cytometer within hours. Collection of nasal fluid on sponges or absorbent filters with measurement of mediators and cytokines has been validated in response to nasal provocation, but not in the context of immunotherapy trials [19], although studies are in progress.

Serum-based assays are feasible in the context of clinical trials and are largely restricted to measurements of antibodies (specific IgE, IgG, IgG4, and IgA) and functional antibody assays, such as detection of serum inhibitory activity for antigen-stimulated basophil activation tests [36] and inhibitory activity for binding of IgE-allergen complexes to B cells [37]. The latter has been shown to persist for at least 2 to 3 years following sublingual immunotherapy [3] and to correlate modestly but more convincingly with clinical response than measurement of serum immunoreactive IgG antibodies alone [38]. At the present time, apart from the use of serum allergen-specific IgE for patient selection [39], there are no biomarkers that can be reliably recommended for selection of individual patients in routine practice for immunotherapy, nor for monitoring the response to treatment. However, rapid advances in the molecular diagnosis of individual allergen IgE sensitivities combined with better information on the constituents of the allergen extracts available for therapy provide an exciting opportunity to relate this new knowledge to prediction of response to immunotherapy [34].

## Chapter 4. Clinical efficacy of sublingual immunotherapy

▪ As of June 2013, there were 77 randomized, double–blind, placebo-controlled (RDBPC) trials of SLIT, of which 62 were conducted with grass or house dust mite (HDM) extracts. The majority of these studies were heterogeneous for allergen dose, duration, and patient selection. All statements on efficacy of SLIT do refer to products which have demonstrated efficacy in appropriate studies.

▪ Seventeen trials, of which one was totally negative, were published after the previous WAO position paper.

▪ The literature suggests that, overall, SLIT is clinically effective in rhinoconjunctivitis and asthma, although differences exist among allergens.

▪ The available meta-analyses are in favor of SLIT (rhinitis and conjunctivitis in adults; asthma and rhinitis in children), although the conclusions are limited by the heterogeneity of the studies in term of doses, duration, and patient selection.

▪ Clinical efficacy and dose dependency have been demonstrated for rhinoconjuntivitis due to grass pollen in adequately powered, well-designed RDBPCs.

▪ Some open, controlled trials suggested that the clinical efficacy of SLIT is similar to that of injection immunotherapy.

▪ Dose-finding trials and large studies with properly defined outcomes and sample sizes are needed for the other relevant individual allergens.

### Double-blind, randomized, placebo-controlled trials

The previous World Allergy Organization (WAO) Position Paper [9] included 60 RDBPCs trials conducted with SLIT. From then through June 2013, 17 new RDBPC trials were published in English [40-55] (Table 1), 8 with grass extracts, 5 with HDM, 1 with *Alternaria*, and 3 with ragweed. Six studies were conducted in children [40,47-49,53,54], and 1 in elderly patients [50]. The number of patients enrolled ranged from about 30 to more than 700, and the duration varied from 4 months to 3 years. Five studies enrolled more than 400 patients [40,44,51,52]. Dropouts and patient dispositions were reported in all trials, and 11 trials [40,42,44,46,50-55] declared a formal sample size calculation. All of the trials but one [53] demonstrated a significant effect of SLIT, independent of the allergen considered. The relative change versus placebo, when reported, ranged between 20% and more than 35%.

**Table 1:** Randomized, double-blind, placebo-controlled trials of SLIT performed since 2009

**Author, year [reference]**	**Ages (y)**	**A/P**	**Dropouts (A/P)**	**Allergen**	**Duration**	**Dose and administration**	**Disease**	**Manu-facturer**	**Main positive results**	**Negative results**
Horak, 2009 [43]	18-50	45/44	3/4	Grass	4 mo	20 mcg	RC	STA	Significant reduction in RC score in Vienna challenge chamber at 4 mo in SLIT vs baseline and vs placebo (the reduction vs placebo was 29%)	Nasal airflow
						Phl p 5/day				Weight of secretions
						Tablets				Basophil activation
									Increased IgE and IgG4	
Skoner, 2010 [46]	18-50	39 med	4	Ragweed	6 mo	4.8 or 48 mcg	RC	GRE	Combined symptoms+drugs and drug score versus placebo	Nasal challenge, IgE
		36 high	5			Amb a 1/day				Symptom score
		40 plac	3			Metered pump				during peak season
Cortellini, 2010 [42]	16-44	15/12	0/1	*Alternaria*	10 mo	60 mcg Alt a 1 cumul. 6 mcg/mo	RCA	ANA	Significant reduction in combined score (-38% versus placebo).	Specifcic IgE and IgG4
						Drops			Significant reduction in skin reactivity	
Panizo, 2010 [45]	18-65	52/26	2/1	Grass	5 mo	25 mcg Phl p 5/day Tablets	RC	ALK	Increase in IgE, IgG4, and IgE blocking activity only in active	
Yonekura, 2010 [48]	7-15	20/11	1/2	Mite	1 y	0.5 mcg Der f 1 once a week	RC	TOR	Significant decrease in symptoms and combined score in wk 0–3 and 37–40 only in SLIT	Medication score
Blaiss, 2011 [40]	5-17	349/358	33/30	Grass	6 mo	450 g Phl p 5/mo	RC	STA	Significant reduction in combined score (-26%) versus placebo. Quality of Life 38% improvement vs placebo	Asthma symptoms
Nelson, 2011 [44]	18-63	213/225	33/33	Grass	10 mo	450 mcg Phl p 5/mo Tablets	RCA	STA	Significant reduction in combined score (-20%and medication score (-20%) versus placebo	Daily medication score
Bush, 2011 [41]	18-50	High 10	2	Mite	18 mo	70 or 1 mcg	RA	GRE	Significant reduction in specific bronchial reactivity	Symptoms and medication scores
		Low 10	3	(Der f)		Der f 1 per dose.				
		Pla 11	5			Drops				
									Increase in IgG4	
Stelmach, 2012 [47]	6-18	Cont 20	3	Grass	2 y	Cumulative 7.3 and 3.6 mcg Phl p 5. Drops	RCA	ALK	Significant improvement in drugs +symptoms with both continuous and precoseasonal regimen. Reduction in FeNO	Symptom score
		Prec 20	1							Medication score
		Pla 20	2							Pulmonary function
de Bot, 2012 [53]	6-18	126/125	15/17	Mite	2 y	4.06 mcg	RC	ART		Symptom score
						Der p 1/week				QoL
						Drops				Medication score
										Well days
Ahmadiasfshar 2012 [49]	5-18	12/12	2/2	Grass	6 mo	Cumulative: about 6,000 IR Spray	RC	STA	Significant improvement in symptom and medication scores	
									Reduction of skin wheal diameter	
Wahn, 2012 [54]	4-12	158/49	26/2	Grass	8 mo	Cumulative: 7.2–8.4 mg group 5	RC	ALL	Significant reduction versus placebo in combined symptom/medication and individual scores	
						Drops				
Cox, 2012 [51]	18-65	233/240	26/17	Grass	6 mo	Cumulative: approx 3.6 mg group 5 allergen.	RC	STA	Significant reduction of combined symptom + medication score (-28% versus placebo) and overall quality of life	Itchy nose symptom score versus placebo
						Tablets				
Bozek, 2013 [50]	60-75	51/57	7/9	Mite	3 y	NS	RC	STA	Total nasal scores decreased by 44% from baseline in SLIT and by 6% in placebo.	Symptoms after specific nasal provocation versus placebo
									Medication score decreased 35% from baseline in SLIT group.	
Wang, 2013 [55]	4-65	60/60	12/23	Mite	6 mo	NS	RC	ZHE	Significant decrease in each individual rhinitis symptom versus placebo starting from week 14.	No change versus placebo in medication intake
Nolte, 2013 [18]	18-50	High 187	142	Ragweed	1 y	6 or 12 mcg	RCA	MSD	Significant decrease in combined symptom + medication score for both active groups VS placebo (21% and 27%)	
		Low 190	overall			Amb a 1 daily				
		Pla 188				Tablets				
Creticos, 2013 [52]	18-50	Low 197	40	Ragweed	1 y	Cumulative dose	RCA	MSD	Only the high dose decreased daily symptom, medication, and combined scores during peak pollen season and whole season versus placebo.	Low dose overall less effective than the 2 other doses on symptoms/medications in peak pollen and whole season
		Med 195	43			4.38 mg Amb a 1				
		High 194	57			Tablets				
		Pla 198	38							

*Abbreviations:* A/P active/placebo, NS not stated, RC rhinoconjunctivitis, RCA rhinoconjuntivitis/asthma, STA Stallergenes, GRE Greer, ANA Anallergo, ALL Allergopharma, ALK ALK-Abellò, MSD Merck Sharp and Dome, TOR Torii Pharmaceuticals, ZHE Zheng Wolwo Bio Pharma.

Of note, the only totally negative trial published after the previous WAO Position Paper [53] was conducted in more than 200 children recruited in a primary care setting. In this case, the authors hypothesized that the amount of allergen given was too low to produce a clinical benefit.

Of the mentioned trials, one was performed using the Vienna challenge chamber [43] and demonstrated that the effect of SLIT begins quite early (at 4 months). This relatively early onset of the effect was confirmed in another trial with natural allergen exposure [55]. One of the trials [45] investigated only immunological parameters and showed a significant effect in the active group but not in the placebo group relative to baseline.

**Figure 3:**
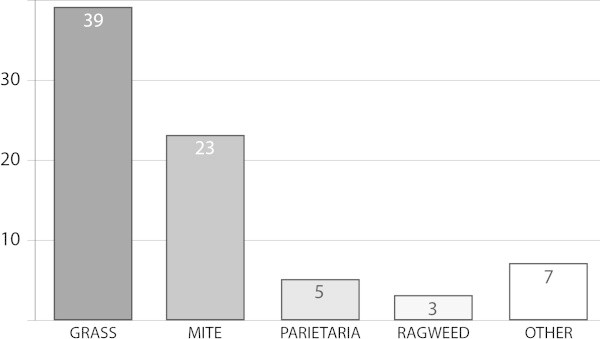
Number of double-blind, placebo-controlled trials investigating SLIT up to June 2013.

Overall, there have now been 77 RDBPC trials investigating SLIT (Figure 3): 39 with grasses, 23 with mite, 5 with *Parietaria*, and 10 with other allergens (ragweed, cypress, cat, olive, birch, cedar). Of the 77 trials, 5 were totally negative. Nonetheless, several trials consistently reported efficacy to be dose dependent (for review see [56]), reinforcing the robustness of the results so far reported in clinical trials.

### Meta-analyses

After the previous WAO position paper [9], 4 new meta-analyses of SLIT for respiratory allergy were published [2,57-59]. So far, the most comprehensive meta-analysis of SLIT in allergic rhinitis is that by Radulovic et al. [58], which included 22 trials and a total of 979 patients. The overall results favored SLIT over placebo for rhinitis symptoms and medication scores. For symptoms, the standardized mean difference (SMD) was –0.42 (95% CI –0.69 to –0.15, *P* = 0.002). For medications, the SMD was –0.43 (95% CI –0.63 to –0.23, *P* = 0.00003). However, great heterogeneity remained (I^2^ between 40% and 95%, depending on the outcome analyzed) due to the largely different inclusion criteria, outcomes, doses, and durations among the trials. In addition, this analysis did not include the most recent large trials. However, the increasing number of available studies has enabled more detailed meta-analyses, for instance according to the allergen or the disease. One meta-analysis was limited to mite [59]. Another that considered only grass allergens [57] clearly showed the superiority of SLIT over placebo for both symptoms and medications. Finally, another meta-analysis limited to conjunctivitis symptoms [2] confirmed a significant difference in favor of SLIT, with an SMD of 0.41 (95% CI 0.28 to 0.53) and moderate heterogeneity (I^2^: 59%). The new meta-analyses substantially confirmed the previous results, showing an overall efficacy of SLIT for different outcomes. However, the great heterogeneity of the results (due to the inherent heterogeneity of trials) still limits the reliability of the results [60,61].

### Comparison of the efficacy of SLIT and SCIT

The problem of comparing the efficacy of subcutaneous immunotherapy (SCIT) and SLIT is still open. The comparison is technically difficult, because head-to-head comparisons need a double-blind, double-dummy design, with a careful choice of outcomes and dosages. Surprisingly, given the technical difficulty, 3 comparative studies [24,27,62] were carried out after the 2009 WAO position paper [9], as summarized in Table 2. The study by Eifan et al. [27] was randomized, open, and controlled and involved 48 children monosensitized to mite. They received SCIT, SLIT, or pharmacotherapy alone for 1 year. The 2 routes of immunotherapy did not differ in terms of clinical efficacy, and both were superior to pharmacotherapy. The second study [24] was a double-blind, double-dummy, 4-parallel-group trial to assess the efficacy and feasibility of SCIT induction followed by SLIT maintenance. The 2 single routes were also compared. The combined regimen did better than the 2 taken separately, with a slight advantage for SCIT alone over SLIT alone on some outcomes. The third study [62] was again a double-dummy study versus placebo in which both treatments achieved a significant clinical improvement versus baseline, with SCIT doing on average better than SLIT in the direct comparison.

**Table 2:** Direct comparisons of SLIT and SCIT for efficacy

**Author, year design**	**Ages (y)**	**Treat-ment**	**Dropouts**	**Allergen**	**Duration**	**Cumulative doses**	**Disease**	**Main results**
Eifan, 2010 [27]	5–12	16 SCIT	2	Mite	1 y	SCIT 111 mg Der p 1/156 mg Der f 1	RA	Significant reduction of total rhinitis and asthma score, medication score, VAS, and skin reactivity *P* < 0.05 versus pharmacotherapy for both SCIT and SLIT. No difference between routes of administration.
Randomized, open, controlled		16 SLIT	1					
		16 CON	2					
						SLIT 295.5 mg Der p 1/f 1		
Keles, 2011 [24]	5–12	15 SCIT	2	Mite	18 mo	Der p 1: 53 mcg SLIT and 42 mcg SCIT	A	Decreased asthma attacks and use of steroids at 4, 12, 18 mo for SCIT and SCIT+SLIT, at 12 mo only for SLIT. No change in VAS for asthma with SCIT or SIT alone.
Double blind, double dummy, controlled		15 SLIT	2					
		15 SLIT	1					
		+ SCIT						
		15 CON	3					
Yukselen, 2012 [62]	7–14	10 SCIT	1	Mite	1 y	173,733 TU (86,866.5 TU *D pt.* and 86,866.5 TU D*f).*	RA	Significant reduction in symptom and medication score versus baseline with both treatments. SCIT better than SLIT versus placebo.
		10 SLIT	1					
Double blind, double dummy, placebo controlled								
		10 PLA	0					

*Abbreviations:* CON control, PLA placebo, SCIT subcutaneous immunotherapy, SLIT sublingual immunotheapy, RA Rhinitis with asthma, A asthma, VAS visual analog scale.

### Non-RDBPC trials

Additional open, randomized, controlled clinical trials were performed after the publication of the previous WAO position paper [9]. These studies, despite their methodological limitations, provided some interesting insights into the efficacy of SLIT. One study compared coseasonal and continuous regimens in children suffering from grass allergy [63]. In this study, the continuous (all year long) regimen started in the preseason. The main results were that in the first pollen season the continuous regimen performed better on symptoms, but starting from the second season, the 2 regimens became more and more similar, so that at the third season no difference could be detected. Another study was conducted in a randomized single-blind fashion for 3 years with an *Alternaria* extract [64]. There was a progressive reduction in symptoms, as measured with a visual analog scale, which became significant after the first year in the active group. Only few mild adverse events were reported. Another open randomized trial in adults (33 patients) showed that a preseasonal regimen was equivalent in efficacy, as measured by visual analog scale, to the continuous administration [65]. These results are further reinforced by those of a previous RDBPC trial comparing precoseasonal and continuous regimens in children [47]. A study in the United States [66] compared the efficacy of HDM SLIT in 134 monosensitized or polysensitized patients. After 1 year, symptoms and drug scores decreased significantly in both groups, but no difference between groups was detected. The same was observed in another trial comparing the quality of life in patients receiving SLIT who were either mono- or polysensitized, where no difference between the 2 groups could be observed [67]. Therefore, the authors concluded that SLIT can also be effective in patients with multiple sensitizations, provided that the clinically relevant allergen is correctly identified [68].

## Chapter 5: Safety of sublingual immunotherapy

• Sublingual immunotherapy (SLIT) appears to be better tolerated than subcutaneous immunotherapy (SCIT).

• SLIT should only be prescribed by physicians with appropriate allergy training and expertise.

• Specific instructions should be provided to patients regarding the management of adverse reactions, unplanned interruptions in treatment, and situations when SLIT should be withheld.

• The majority of SLIT adverse events are local reactions (e.g., oromucosal pruritus) that occur during the beginning of treatment and resolve within a few days or weeks without any medical intervention (e.g., dose adjustment, medication).

• A few cases of SLIT-related anaphylaxis have been reported but there have been no fatalities.

• Risk factors for the occurrence of SLIT severe adverse events (SAEs) have not yet been established, although there is some suggestion that patients who have had prior systemic reactions to SCIT may be at increased risk.

• There is a need for a generally accepted system of reporting allergen immunotherapy (AIT) adverse reactions that is applicable to both clinical practice and research.

○ A uniform classification system for grading for AIT systemic reactions has been developed.

○ A classification system for grading SLIT local reactions has also been developed.

○ Consistent use of the Systemic Reaction and SLIT Local Reaction Grading Systems is recommended.

### Classification and frequency of SLIT adverse events

One of the purported advantages of SLIT over SCIT is greater safety, which allows for administration of this treatment outside of the medical setting. In a comprehensive review of 104 articles on SLIT, there were 66 studies that provided some information on safety and tolerance, representing 4378 patients who received approximately 1,181,000 SLIT doses [69]. Oromucosal reactions, considered a SLIT local reaction, were relatively common, affecting up to 75% of patients, and were seen most frequently in the build-up phase. In the studies that specified the type of reaction, 169 (0.056%) of 314,959 doses administered were classified as producing systemic reactions. To provide some perspective, in an American Academy of Allergy Asthma & Immunology/American College Of Allergy Asthma & Immunology (AAAAI/ACAAI) 3-year immunotherapy safety surveillance study, the incidence of reported SCIT systemic reactions was 0.1% of injections, the majority of which were classified as Grade 1 (cutaneous or upper respiratory symptoms) [70,71].

Since the publication of the first WAO SLIT Position Paper [9], at least 5 large randomized double-blind placebo-controlled (RDBPC) trials that included more than 100 patients have been published [40,44,46,51,53]. In these trials, the overall occurrence of side effects did not differ from the occurrence in previous studies, and no new safety concerns were raised. Local side effects (oral and gastrointestinal) continue to represent the majority (~80% to 90%) of all reported adverse reactions. Long-term follow-up of some of the large trials indicated that the number and intensity of AEs tend to decline with additional courses of SLIT [3,72].

The amount of information provided about AEs varied greatly in many of the earlier SLIT studies; some included only general summary statements, such as “no relevant side effects,” whereas others provided detailed analyses of the AEs [69]. One consideration with SLIT is that the majority of doses are administered outside of the clinic setting with no direct medical supervision, and the accuracy of the AE reporting depends on the patient’s and/or family’s recall and interpretation of the event. The heterogeneity in classifying and reporting SCIT and SLIT adverse reactions in clinical trials makes comparisons and analysis of safety difficult. In recognition of the need for a uniform classification system for AIT adverse reactions, an international Task Force composed of members of the academic, clinical, and research allergy communities was formed. Using existing grading systems as a template, the Task Force combined information from the members’ clinical experience and data from published studies and safety surveys to develop a 5-grade classification system for systemic AEs (the World Allergy Organization Subcutaneous Immunotherapy Systemic Reaction Grading System, Table 3) [73]. Although the grading system was developed for SCIT, it also applies to SLIT. The grade is based on the organ system(s) involved and severity and is to be determined by the physician’s clinical judgment after the event is over.

**Table 3:** World Allergy Organization subcutaneous systemic reaction grading system

**Grade 1**	**Grade 2**	**Grade 3**	**Grade 4**	**Grade 5**
Symptoms(s)/sign(s) of one organ system present:*	Symptoms(s)/sign(s) of more than one organ system present:	**Lower respiratory**	**Lower or upper respiratory**	Death
		Asthma (eg, 40% PEF or FEV1 drop, NOT responding to an inhaled bronchodilator)	Respiratory failure with or without loss of consciousness	
**Cutaneous**				
Generalized pruritis, urticaria, flushing, or sensation of heat or warmth**				
**or**	**or**	**or**	**or**	
Angioedema (not laryngeal, tongue, or uvular)	**Lower respiratory**	**Upper respiratory**	**Cardiovascular**	
**or**	Asthma: cough, wheezing, shortness of breath (eg, <40% PEF or FEV1 drop, responding to an inhaled bronchodilator)	Laryngeal, uvula, or tongue edema with or without stridor	Hypotension with or without loss of consciousness	
**Upper respiratory**				
Rhinitis (eg, sneezing, rhinorrhea, nasal pruritis, and/or nasal congestion)				
**or**	**or**			
Throat-clearing (itchy throat)				
**or**	**Gastrointestinal**			
Cough perceived to come from the upper airway, not the lung, larynx, or trachea	Abdominal cramps, vomiting, or diarrhea			
**or**	**or**			
**Conjunctival**				
Conjunctival erythema, pruritus, or tearing	**Other**			
	Uterine cramps			
**Other**				
Nausea, metallic taste, or headache				
Patients may also have a feeling of impending doom, especially in grades 2, 3, or 4.				
**Note:** children with anaphylaxis seldom convey a sense of impending doom and their behavior changes may be a sign of anaphylaxis, eg, becoming very quiet or irritable and cranky.				
**Scoring includes a suffix that denotes if and when epinephrine is administered in relationship to symptom(s)/sign(s) of the SR: a, ≤5 minutes; b, >5 minutes to ≤10 minutes; c, >10 to ≤20 minutes; d, >20 minutes; z, epinephrine not administered.**				
The final grade of the reaction will not be determined until the event is over, regardless of the medication administered. The final report should include the first symptom(s)/sign(s) and the time of onset after the subcutaneous allergen immunotherapy injection^†^ and a suffix reflecting if and when epinephrine was administered, **eg, Grade 2a; rhinitis: 10 minutes.**				
**Final report: Grade, a-d or z ________________First symptom ________________ Time of onset of first symptom ________________**				
**Comments**^††^				

*Each Grade is based on organ system involved and severity. Organ systems are defined as: cutaneous, conjunctival, upper respiratory, lower respiratory, gastrointestinal, cardiovascular, and other. A reaction from a single organ system such as cutaneous, conjunctival, or upper respiratory, but not asthma, gastrointestinal, or cardiovascular is classified as a Grade 1. Symptom(s)/sign(s) from more than one organ system or asthma, gastrointestinal, or cardiovascular are classified as Grades 2 or 3. Respiratory failure or hypotension, with or without loss of consciousness, defines Grade 4 and death Grade 5. The grade is determined by the physician’s clinical judgment.

**This constellation of symptoms may rapidly progress to a more severe reaction.

^†^Symptoms occurring within the first minutes after the injection may be a sign of severe anaphylaxis. Mild symptoms may progress rapidly to severe anaphylaxis and death.

^††^If signs or symptoms are not included in the Table or the differentiation between an systemic response and vasovagal (vasodepressor) reaction, which may occur with any medical intervention, is difficult, please include comment, as appropriate.

Adapted from [73]. Reprinted with permission from Elsevier Publishers.

Local side effects are the most frequent adverse reactions associated with SLIT, and these local reactions can be severe and/or bothersome enough to cause treatment discontinuation. In the previously discussed review [69], SLIT adverse reactions accounted for treatment withdrawal in 3% of the SLIT patients, compared with 1.4% of the placebo-treated patients. Local adverse reactions are often the reason cited for treatment discontinuation in both RDBPC and observational studies [69,74,75]. Similar to the need for a grading system for systemic events, a uniform system for grading the severity of local AEs was perceived as necessary for uniform reporting and classification of SLIT local adverse reactions. A WAO Task Force was formed for the purpose of developing a classification system for grading SLIT local reactions [76]. The Task Force examined the clinical trials and the postmarketing surveillance data, and considered the MedDRA nomenclature [77] (Table 4) in the development of the clinically based 3-grade classification system for local SLIT reactions (Table 5). This grading system is primarily based on the patient’s subjective reporting, with a severe reaction (Grade 3) being one that leads to treatment discontinuation. Note that gastrointestinal symptoms associated with SLIT can be classified as either local reactions, if only oromucosal symptoms are present, or as a systemic reaction, if occurring in conjunction with other systemic symptoms.

**Table 4:** **Description of the local side effects related to SLIT (MedDRA 14.1)**[78]

	**Local side effect**	**MedDRA preferred term**	**MedDRA CODE**	**MedDRA low level term (LLT)**
Mouth/ear	Altered taste perception	Dysgeusia	10013911	Taste alteration
	Itching of lips	Oral pruritus	10052894	Itching mouth
	Swelling of lips	Lip swelling	10024570	Swelling lips
	Itching of the oral mucosa	Oral pruritus	10052894	Itching mouth
	Swelling of the oral mucosa	Oedema mucosal	10030111	Mucosal swelling
	Itching of the ears	Ear pruritus	10052138	Ear pruritus
	Swelling of the tongue	Swollen tongue	10042727	Tongue swelling non-specific
	Glossodynia	Glossodynia	10018388	Glossodynia
	Mouth ulcer	Mouth ulceration	10028034	Mouth ulcer
	Tongue ulcer	Tongue ulceration	10043991	Tongue ulceration
	Throat irritation	Throat irritation	10043521	Throat irritation
	Uvular edema	Pharyngeal oedema	10034829	Pharyngeal oedema
	Nausea	Nausea	10028813	Nausea
Upper gastro-intestinal	Stomach-ache	Abdominal pain upper	10000087	Stomach ache
	Vomiting	Vomiting	10047700	Vomiting
	Abdominal pain	Abdominal pain	10000081	Abdominal pain
Lower gastro-intestinal	Diarrhea	Diarrhoea	10012735	Diarrhea

**Table 5:** WAO Grading system for SLIT local adverse events

**Symptom/sign (see Table**1**)**	**Grade 1 – Mild**	**Grade 2 – Moderate**	**Grade 3 - Severe**	**Unknown severity**
Pruritus/swelling of mouth, tongue or lip	Not troublesome	Troublesome	• Grade 2	The treatment is discontinued but there is no subjective and/or objective description of the severity from the patient/physician
Throat irritation	**AND**	**OR**	**AND**	
Nausea	No symptomatic treatment required	Requires symptomatic treatment	• SLIT discontinued because of local side effects	
Abdominal pain	**AND**	**AND**		
Vomiting	No discontinuation of SLIT because of local side effects	No discontinuation of SLIT because of local side effects		
Diarrhea				
Heartburn				
Uvular oedema				
**Each local adverse event can be early (<30 minutes) or delayed**				

From [76]. Reprinted with permission from Elsevier.

### SLIT serious adverse events

In the SLIT comprehensive review [69], there were no fatalities or SLIT-related systemic reactions associated with hypotension, although there were 14 probable SLIT-related SAEs in 3984 patients treated with a total of 1,019,826 doses. This represents 1.4 SAEs per 100,000 SLIT doses. The most common SLIT-related SAEs were asthmatic reactions (n = 7), one of which required hospitalization; the others were abdominal pain/vomiting (n = 3), uvula edema (n = 1), and urticaria lasting 48 hours. Subsequent to this review, there have been a few case reports of systemic reactions of a severity that should be categorized as anaphylaxis (Table 6) [79-86]. In 2 case reports, 4 patients had experienced systemic reactions with prior SCIT treatment [79,85]. Two of these 4 experienced anaphylaxis with the first SLIT tablet [79].

**Table 6:** Characteristics of the SLIT-induced anaphylaxis reported in literature

**Author, year [reference]**	**Sex (age)**	**Allergen (producer)**	**Phase**	**Onset**	**Description**	**Epinephrine**
De Groot, 2009 [79]	M (13)	Grass (Grazax, ALK-Abellò)	First dose	15 min	Generalized urticaria, swelling of tongue	No
De Groot, 2009 [79]	F (27)	Grass (Grazax, ALK-Abellò)	First dose	5 min	Abdominal cramps, asthma, generalized itching, hypotension	Yes (SC)
Blazowski, 2008 [80]	F (16)	HDM (Staloral, Stallergenes)	Maintenance overdose (60 drops)	10 min	Hypotension-collapse, flushing, urticaria	Yes (IM)
Eifan, 2007 [81]	F (11)	Mixture (dust mite + grass pollen mix (Stallergenes)	Maintenance	3 min	Abdominal pain, chest pain, fever, nausea	Not specified
Dunsky, 2006 [82]	F (31)	*Alternaria*, cat, dog grass, ragweed, (Greer)	2nd day of updosing	5 min	Angioedema, dizziness, dyspnea, generalized itching	No
Antico, 2006 [83]	F (36)	Latex	End of rush buildup	10 min	Asthma, generalized urticaria	Not specified

SC: subcutaneous IM: intramuscular.

### Risk factors for SLIT adverse events

No clear predictors for SLIT AEs have been identified, although some of the factors in the SLIT anaphylaxis case reports are recognized as risk factors for SCIT: height of season [81], history of previous systemic reactions [79], dose [80], and accelerated schedules [83]. In addition, most of the patients with SLIT-related SAEs or anaphylaxis had asthma; symptomatic asthma has been identified as a risk factor for AEs with SCIT [70,87].

The numbers have been too small to identify risk factors for SLIT SAEs. In 6 case reports of SLIT-associated anaphylaxis [79-84], 5 of 6 patients were female, all were adolescents or young adults, 5 of the 6 had a history of asthma, and 2 had a previous history of severe reactions to SCIT. In a study evaluating the safety of SLIT in 43 patients, 3 of the 5 patients who experienced a SLIT systemic reaction had a history of a previous SCIT systemic reaction [86]. Prior systemic reaction with SCIT was identified as a possible risk factor in 4 patients in case reports [79,85].

In general, patients receiving SLIT are not prescribed injectable epinephrine in the event of a rare systemic reaction. However, in the study of 43 patients that identified prior SCIT systemic reaction as a possible predictor for SLIT systemic reaction, injectable epinephrine was prescribed as an ethics committee requirement, and 2 patients used it [86]. The Federal Drug Administration (FDA) has stipulated a similar requirement for prescribing epinephrine autoinjectors for the subjects participating in US SLIT clinical trials. In 2 of these studies, which together included 345 children and 439 adults, a small number of patients in the placebo (n = 2) and SLIT (n = 4) groups used the epinephrine autoinjector [40,44]. In 3 instances, it was used for symptoms not caused by active treatment. One patient in the placebo group used it 12 hours after the 137^th^ dose because of wheezing related to exposure to a grassy field [40], and another placebo patient used it in response to what was subsequently deemed to be an anxiety attack. In the SLIT group, self-administered epinephrine was used in 4 patients for symptoms diagnosed later as viral pharyngitis [40], flushing and chest tightness [44], lip angioedema and cough [40], and uvula/pharyngeal edema [85]. However, in other US clinical trials that collectively included over 1000 patients, there was no use of the epinephrine autoinjectors for SLIT-related reactions [46,51,52], and epinephrine autoinjectors are not routinely prescribed or recommended in countries where SLIT is registered and commercially available. The relatively rare and potentially inappropriate use of injectable epinephrine in these studies raises concerns about the benefits and harms of routine prescribing of injectable epinephrine, which may become standard practice if the product information for an FDA- approved SLIT formulation includes this recommendation. In addition, the US clinical trials required that the first SLIT dose be administered in the study site. Administration of the first dose in a medically supervised setting has never been a requirement in the European Union.

### Allergen dose, formulation, and adverse reaction rate

The literature does not appear to show a consistent correlation between the administered SLIT dose and the rate or severity of AEs [69]. For example, one study of dust mite–allergic asthmatic children that employed a relatively low-dose dust mite SLIT (15 mcg cumulative monthly dose [CMD] of Der p 1) reported a systemic reaction rate of 0.46% per dose [88]. In contrast, another study of dust mite–allergic asthmatic children treated with a CMD 50 times greater (783 mcg CMD of mixed mite) reported no serious AEs and no significant difference in the incidence of AEs between the SLIT and placebo groups [89]. A relationship between dose and the frequency and severity of AEs has been demonstrated in some allergen dose-response studies [90,91], but so far a “maximum tolerated dose” has not been documented for SLIT.

Tolerability may vary with the extract and formulation. A dose-response study compared the safety of 6 doses of ragweed tablets (3, 6, 12, 24, 50, or 100 U Amb a 1) in 53 subjects with ragweed-induced allergic rhinitis. Recruitment to 50 U Amb a 1 was discontinued, and the 100 U Amb a 1 dose was not initiated after 3 subjects experienced systemic reactions at doses ≥24 U Amb a 1 [91]. In contrast, the treatment and placebo groups had similar frequencies of systemic AEs in an RDBPC dose-response study of 115 ragweed-allergic rhinitis patients randomized to receive 4.8 or 48 mcg of Amb a 1 sublingual ragweed extract solution [46]. Similar safety was demonstrated with this same ragweed extract solution in a subsequent RDBPC study of 429 patients who received up to 50 mcg of Amb a 1 or placebo [92]. The difference in tolerability between similar ragweed doses may be a result of the formulation (tablet versus extract solution). However, studies with grass pollen sublingual tablets and extract solution have demonstrated comparable dose efficacy and safety [54,93,94]. One study comparing the safety of 7 doses of grass tablets, with the highest dose equivalent to 200 mcg of Phl p 5, reported no treatment-related AEs that were serious, systemic, or led to withdrawal [90]. These studies reaffirm that an effective and safe dosing regimen will need to be established for each allergen extract formulation.

### Induction schedule

In contrast to SCIT, accelerated induction schedules with SLIT do not appear to be associated with a greater risk of systemic reaction. Rush, ultra-rush, and no-induction SLIT schedules seem to be tolerated as well as multi-dose, multi-week induction schedules.

Several large multicenter studies, collectively including over 1000 patients, investigated the safety and efficacy of grass and ragweed tablets administered without an updosing phase. There were few reported systemic allergic reactions, primarily WAO Grade 1 or 2, and no Grade 4 reactions [40,44,51,94,95]. Similar safety has been reported with SLIT ultra-rush and rush induction schedules, which allow patients to achieve the target maintenance dose within minutes to hours [46,96-98].

Although the induction phase does not seem to influence the SLIT AE rate, many studies have reported that more AEs occurred during the induction phase than during the maintenance phase. Most occur within the first few days to weeks of treatment and infrequently after this initial phase. The local AEs appear to resolve without any medical interventions, such as dose adjustments or antihistamines. In studies that have utilized discontinuous schedules (pre- and coseasonal), the frequency and intensity of AEs appeared to decline in the later courses of SLIT treatment [3,72,99].

### SLIT in young children

Immunotherapy guidelines do not specify a particular lower age limit for initiating AIT. SCIT is often not prescribed to young children, primarily because of concerns that they may have difficulty cooperating with an immunotherapy program and, in particular, in communicating symptoms of systemic reactions. However, studies that have evaluated the safety of SCIT in children less than 5 years old have reported a similar incidence and severity of AEs as in other age populations [100,101]. Citing these studies, the third update of Allergen Immunotherapy: A Practice Parameter [102] states that:

Immunotherapy can be initiated in young children less than 5 years of age if indicated. Indications should be based on the severity of the disease, risk/benefit ratios, and the ability of the physician to correlate the clinical presentation with appropriate and obtainable allergy testing.

The preventive benefits of AIT may be greater if initiated early in the course of the allergic disease [103]. SLIT’s favorable safety profile and a regimen that does not require needles or frequent trips to a medical clinic may make this disease-modifying treatment more available and attractive to young children and their caregivers, allowing for treatment initiation at an age where disease progression may be more easily influenced.

Observational and postmarketing survey studies specifically designed to evaluate the safety of SLIT in young children found that most reactions were mild or moderate and resolved without treatment [104-106]. Dose reduction by changing from a sublingual-swallow to a sublingual-spit method controlled gastrointestinal reactions in one study [106]. One further study with dust mite SLIT in 138 children aged 2–5 years with asthma or rhinitis showed only mild to moderate local AEs [107].

Recognizing the importance of treatment adherence for SLIT efficacy, one study evaluated the adherence to SLIT in 150 children <6 years old over a 2-year period [74]. Overall, 45% of the children discontinued treatment. The percentage of discontinuations was significantly higher in the children <4 years old than in the older children, with all discontinuations in this age group occurring within the first 3 months of treatment. The most common cause for withdrawal in the children <4 years old was “… the subjective discomfort in keeping under the tongue drops/tablets, or children’s refusal, without apparent side effects.” The parents attributed their children’s refusal to unpleasant taste. There was no difference in adherence between tablets or drops in the older children, but tablets were discontinued in 100% of the children <4 years old. There was one asthma reaction leading to SLIT withdrawal in the <4-year-old group. The authors speculated that the local SLIT side effects are more troublesome in young children and suggested that SLIT would best be started after age 4 because of poor adherence in younger children.

### Multiallergen SLIT

Two of the case reports of SLIT anaphylaxis involved multiallergen SLIT, and most of the SLIT studies employed single allergens. However, 2 studies specifically designed to compare the safety of single versus multiallergen SLIT in adults and children found no significant differences in terms of frequency or severity of treatment-related AEs [108,109]. Other studies designed to compare the efficacy of single and multiallergen SLIT have reported similar safety for both [110,111]. Collectively, these studies suggest that multiallergen SLIT is not associated with a greater safety risk than single allergen treatment. However, questions remain as to whether multiallergen immunotherapy is effective [68,110].

### SLIT safety special considerations: autoimmunity, immunodeficiency, and pregnancy

In general, AIT has been studied in adults and children without any significant concomitant chronic illness, and no controlled studies have evaluated effectiveness or risks associated with immunotherapy in patients with immunodeficiency or autoimmune disorders. However, case reports [112-114] and registry studies [115] have been reassuring in terms of the safety of AIT in these conditions. The immunotherapy practice guidelines state that “Immunotherapy can be considered in patients with immunodeficiency and autoimmune disorders” [102]. Most of these studies were based on SCIT, but these findings should apply to SLIT because the immunological mechanism between the routes are thought to be the similar [8].

Generally, AIT is not initiated during pregnancy but can be continued without updosing if treatment was begun prior to conception. There have been no randomized, controlled, prospective studies investigating the safety of AIT during pregnancy. However, retrospective studies of women who received SCIT during pregnancy suggest that there is no greater risk of prematurity, fetal abnormality, or other adverse pregnancy outcome [116,117]. A 6-year study evaluating the safety of SLIT during pregnancy found a lower incidence of abortion, perinatal mortality, prematurity, toxemia, and congenital malformation in 155 women who received SLIT than in the general population [118]. In 24 women, SLIT was initiated for the first time during pregnancy.

Although these studies are reassuring in terms of the safety of SLIT during pregnancy, the numbers are small and the risks and benefits should be considered on an individual basis. A similar approach should be taken when considering SLIT for patients with an immune deficiency or autoimmune disorder, conditions for which there have been no studies specifically investigating the safety of SLIT, although there is indirect evidence from SCIT supporting the safety of AIT in these conditions.

### SLIT safety: patient selection and instructions

Because this treatment is administered at home without direct medical supervision, patients should be provided with specific instructions regarding how to manage adverse reactions or unplanned treatment interruptions, when and what to report to the prescribing physician, and situations when SLIT should be withheld (e.g., oropharyngeal infection, oral abrasion, acute gastroenteritis, asthma exacerbation). Careful consideration should also be given to the ability of the patients or their families to adhere to these instructions and the treatment regimen.

### SLIT safety summary

In general, SLIT appears to be associated with fewer and less severe AEs than SCIT. Oropharyngeal reactions are the most common AEs with SLIT, but other reactions, such as asthma, urticaria, and abdominal pain have been reported. There have been a few case reports of anaphylaxis with SLIT, including 2 reports of anaphylaxis with the first dose. Risk factors for SLIT AEs have not been clearly established. Some studies suggest a greater frequency of AEs during the induction phase than in the maintenance phase, but there does not seem to be a relationship between induction schedule and SLIT AEs, with ultra-rush and no-induction schedules reported as being well tolerated in several studies.

Because this treatment is administered in a medically unsupervised setting, it is particularly important for further studies to be conducted to help identify and characterize SLIT risk factors, appropriate patients, patient instructions to minimize and/or treat AEs, and methods or interventions to monitor and optimize patient adherence.

### Unmet needs

Several issues regarding the safety of SLIT remain unresolved:

• Is SLIT safe in individuals with moderate to severe asthma?

○ Are there specific precautions to be taken for asthma patients before taking SLIT, such as obtaining peak flow measurement?

• Is SLIT safe in patients who have had systemic reactions with SCIT?

• Interruptions in treatment:

○ After how long an interruption between doses is it safe to resume the usual dose

▪ during the updosing phase?

▪ during the maintenance phase?

○ Would the recommendations for interruptions in maintenance treatment be different for regimens with an updosing phase than regimens without an updosing phase?

• Is it safe to administer all formulations of SLIT without induction? Or do some require an updosing phase?

• Are oropharyngeal infections or lesions (e.g., apthous ulcers, gingivitis, eosinophilic esophagitis) risk factors for SLIT systemic reactions?

• Under which clinical situations should a SLIT dose be withheld (e.g., recent respiratory tract infection, recent exacerbation of asthma, gastroenteritis)?

• Is SLIT safe in pregnant or breastfeeding women?

• Is SLIT safe in patients with immune deficiency and autoimmune conditions?

• Are there any risk factors that identify which patients may experience a systemic reaction with SLIT?

## Chapter 6: Impact of sublingual immunotherapy on the natural history of respiratory allergy

• Allergen-specific immunotherapy may alter the natural history of respiratory allergy by preventing the onset of new skin sensitizations and/or reducing the risk of asthma onset.

• Several randomized, double–blind, placebo-controlled (RDBPC) studies in grass pollen rhinoconjunctivitis confirm the persistence of the clinical effects of SLIT for at least 1–2 years after treatment discontinuation.

• There are 2 randomized, open, controlled studies suggesting that SLIT reduces the risk of asthma onset in children with rhinitis. A 5-year prospective RDBPC trial [119] (n = 812 at randomization) in children aged 5–12 years with grass pollen seasonal rhinoconjunctivitis will complete in 2015 and should provide more definitive information.

• Two open, randomized studies have shown that SLIT reduces the onset of new allergen sensitizations. Further RDBPC trials are required.

Sublingual immunotherapy (SLIT) has been shown to be effective in reducing symptoms and medication requirements and in improving quality of life in adults and children with seasonal allergic rhinitis. A particular feature of immunotherapy, unlike usual anti-allergy drugs, is the ability to modify the course of allergic disease. Long-term benefits continue after discontinuation of treatment, and evidence supports reductions in the onset of new sensitizations and the likelihood of progression from rhinitis to asthma in patients with seasonal allergic rhinitis treated for 3 years with subcutaneous immunotherapy (SCIT) (reviewed in the 2009 WAO SLIT Position Paper [9]).

In the previous WAO SLIT position paper [9], preliminary data were presented supporting the long-term benefits of SLIT, largely from small and/or non-randomized controlled trials. There are now convincing data from RDBPC studies of grass pollen SLIT [4,96,120] that show persistent clinical benefit for at least 1 or 2 years following treatment discontinuation (Table 7), illustrating that SLIT as well as SCIT is able to modify the disease and provide long-term benefit. There is also a single open, pharmacotherapy-controlled study in mite-allergic adults followed for a total of 10 to 12 years [121] that provides preliminary data that house dust mite SLIT may provide long-term protection after treatment discontinuation, although further studies are required.

**Table 7:** Long-term efficacy of sublingual immunotherapy after discontinuation of treatment

**First author publication year [ref]**	**Diagnosis**	**Age (years)**	**Study design**	**n Baseline**	**Allergens**	**Allergen daily dose**	**Treatment duration (years)**	**Years after cessation**	**Years blinded**	**n End of follow-up**	**Main results**
**OTT** 2009 [5]	ARC	8–65	RDBPC	213*	5 grass pollens: *D. glomerata, P. pratensis, L. perenne, A. odoratum.* and *P. pratense*	300 IR/mL (21 mcg of Phl p 5)	3	1	3	91**	In the third season, the median of the combined symptom and medication scores had decreased by -–44.7% in the SLIT group and -14.7% in the placebo group compared with baseline values. Symptom scores were reduced by 39.7% in the SLIT group and 1.51% in the placebo group (*P* < 0.05).
											Reductions in combined scores (*P* = 0.0508) and symptom scores (*P* = 0.0144) were observed in the participants treated with SLIT during follow up.
**Marogna** 2010 [6]^2^	AR with or without asthma	18-–65	Open, pharmacotherapy controlled trial	78	House dust mite	10,000 RAST units/mL; 3 times per week	3 to 5	10 to 12	None	59	The clinical effects persisted for 7 years for those on SLIT for 3 years and for 8 years for on those on SLIT for 4 or 5 years. New sensitizations occurred in all the control subjects and in up to 25% of those on SLIT.
**Durham** 2012 [4]	ARC with or without asthma	18–65	RDBPC	634*	Single grass tablet: *Phleum pratense*	75,000 SQ-T/2,800 BAU (15 mcg of Phl p 5)	3	2	5	241	The mean rhinoconjunctivitis daily symptom score was reduced by 25% to 36% (*P* ≤ 0.004) in the SLIT group compared with the placebo group over the 5 grass pollen seasons. The rhinoconjunctivitis DMS was reduced by 20% to 45% (*P* ≤ 0.022 for seasons 1–4; *P* = 0.114 for season 5), and the rhinoconjunctivitis combined score was reduced by 27% to 41% (*P* ≤ 0.003) in favor of active treatment.
											The percentage of days with severe symptoms during the peak grass pollen exposure was in all seasons lower in the active group than in the placebo group, with relative differences of 49% to 63% (*P* ≤ 0.0001). Efficacy was supported by long-lasting significant effects on the allergen-specific antibody response.
**Didier** 2013 [3]	ARC	18–51	RDBPC	633*	5 grasses tablet: *D. glomerata, P. pratensis, L. perenne, A. odoratum,* and *P. pratense*	300 IR (25 mcg - group 5 major allergens)†	3	1	3	435	For the year 4 pollen period, significant reductions in the AAdSS LS means were observed for both the 300IR (4M) and 300IR (2M) groups, -22.9% and -28.5% respectively (compared to placebo).
											There was no significant difference between the 2 active treatment groups, and no impact of asthma or sensitization status on the efficacy results.

**ARC**: Allergic rhinoconjunctivitis; **AR**: Allergic rhinitis; **RDBPC**: Randomized, double-blind, placebo-controlled trial; **SP**: Supralingual; **LS**: Least-Squares; **AAdSS**: Average Adjusted Symptom Score; **DMS**: Daily medication score; **IR**: Index of Reactivity; **SQ-T**: Standardized Quality Tablets; **BAU**: Bioequivalent allergy unit;

*Randomized (n); **Per protocol population; †Placebo or a 300IR tablet daily beginning either 4 months (4M) or 2 months (2M) prior to each pollen season and continuing for its duration for 3 consecutive years.

Following on from the preventive allergy treatment (PAT) study [122], which suggested that SCIT may prevent the onset of asthma for 10 years, the Grass Asthma Prevention (GAP) trial [119] has been initiated and is due to be completed in 2015. In this study, 810 children aged 5–12 years who have grass pollen rhinoconjunctivitis will receive sublingual single-grass pollen extract allergy immunotherapy tablets daily for 3 years, followed by 2 years of continued blinded follow up. The primary end point is the time to the onset of asthma.

In summary, since the previous position paper there is now good evidence for long-term efficacy after treatment discontinuation and a disease-modifying effect of SLIT in grass pollen rhinoconjunctivitis. Further studies to assess the impact of SLIT for rhinoconjunctivitis on the onset of new sensitizations and asthma are in progress. Similar studies for perennial allergies represent an important unmet need.

## Chapter 7: Efficacy of SLIT in children

• Grass-pollen sublingual immunotherapy (SLIT) is effective in seasonal allergic rhinitis in children ≥5 years of age.

• Grass-pollen SLIT is probably effective in seasonal allergic rhinitis in children ≥4 to <5 years of age.

• Grass or house dust mite (HDM) SLIT can be used for allergic rhinitis in children with asthma.

• Pre-coseasonal SLIT with grass pollen in children might be as effective as continuous treatment.

• SLIT must not be suggested as monotherapy for treating asthma.

• House dust mite SLIT is effective in children with asthma and allergic rhinitis.

• More large randomized trials are needed, especially with HDM SLIT in children.

• No new data on the preventive effect of SLIT in children have been published.

A total of 51 manuscripts were identified that contained original studies that had been published since the first WAO SLIT position paper [123], beginning in 2009 (PubMed search with the terms SUBLINGUAL AND IMMUNOTHERAPY and the filters: publication date 2009–; Humans; Clinical Trial; Randomized Controlled Trial; Child: birth–18 years). Twenty-six articles were excluded: 2 had already been included in the original WAO SLIT paper [124,125], 1 was on subcutaneous immunotherapy (SCIT), 1 was for a non-allergic indication, and 22 had almost exclusively adult patients. The remaining 25 original articles form the basis for this update.

All included papers were evaluated with the GRADE system on the quality of scientific evidence from 4 (high) to 1 (very low), as described [126]. A table with the complete GRADE evaluation can be found in a recent publication [127].

Three of the 25 papers only evaluated the safety of SLIT. Of the 21 that evaluated efficacy, the prime allergic disease was allergic rhinitis/rhinoconjunctivitis in 14 papers, allergic asthma in 3, both asthma and rhinitis in 3, and food allergy in 2 (Table 8).

**Table 8:** Clinical efficacy of SLIT in children: update 2009 to September 2012

**AUTHOR, year [reference]**	**Age (y)**	**A/P**	**Drop-out (A/P)**	**Allergen, drop or tablet**	**Duration**	**Dose (mcg/dose and dosing frequency)**	**Versus SCIT**	**Disease**	**Manu-facturer**	**Main positive results**	**Negative results**
***Randomized, double-blind, placebo-controlled***											
Wahn 2012 [11]	4–12	158/49	26/2	6-grass drops	8 mo	40 mcg group 5 daily	NS	RC(A)	All Pharm	**SLIT vs placebo:** Change in pre-post treatment higher for symptom-medication, symptom, & medication scores in SLIT group. Higher rate of positive response with SLIT (≥40% decrease of the AUC of the symptom-medication score).	**SLIT vs placebo:** Mean number of well days
Stelmach, 2012 [17]	6–18	Cont 20	1	Grass, drops	2 y	10 mcg group 5 daily Cont: for 2 y	NS	RCA	Stal	**Both active groups vs placebo:** Significant improvement, med+symp score, symptom score, FeNO.	Medication score in continuous group.
		Pre-co 20	3								
		Plac 20	2								Pulmonary function tests, Metacholine challenge
						Pre-co: 2 × 6 mo					
										Pre-coseasonal group vs placebo: significant reduction of med score	
De Bot, 2012 [12]	6–18	126/125	15/17	Mite, drops	2 y	2.03 mcg Der p 1 twice per week. Total cumulative dose (2 y): 435 mcg	NS	RC	ART	No positive results	Total nasal symptom score, QoL, med score, well days
Yukselen 2012 [19]	Mean 10 (± 3)	SLIT 11	1	Mite, drops	1 y (+1 y obser-vation)	Dpt+Df: SLIT: 1000 TU/mL: 28 drops 3×/week.	4.2	R&A	AllerPhar	**SCIT vs SLIT:** SCIT reduced asthma symptoms significantly more than SLIT.	**SLIT vs placebo:** NS for all clinical parameters. NS for rhinitis and asthma VAS.
		SCIT 10	0								
		Plac 11	1								
	
										**SCIT vs placebo:** Rhinitis symptoms, asthma symptoms, total symptoms, rhinitis meds, and asthma meds improved. VAS score was significantly reduced for both rhinitis and asthma. **SLIT and SCIT vs. baseline year:** both improved almost all clinical parameters	
						SCIT: 3368 TU/4 wk					**SCIT vs SCIT:** NS for rhinitis symptoms and meds and asthma meds.
Blaiss 2011 [10]	5–17	175/169	33/29	Grass, tablets	6 mo	15 mcg Phl p5 daily	NS	RC(A)	ALK	**SLIT:** Improvements in daily symptoms (25%), daily meds (81%), total score (26%), and QoL (18%) (all *P* ≤ 0.04 vs. placebo).	Asthma symptoms
Kim 2011 [20]	1–11	11/7	0/0	Peanut, drops	12 m	2000 mcg daily (8 pumps)	No data on SCIT dosing	Peanut allergy	Greer	Food challenge: Significantly greater safe ingestion of peanut than placebo group; improvements in skin prick test and basophil responsiveness.	No statistically significant changes were found in IL-13 levels, the percentage of regulatory T cells, or IL-10 and IFN-gamma production.
Yonekura, 2010 [26]	7–15	20/11	1/2	Mite, drops	1 y	0.5 mcg Der f 1 once a week	20	RC	TOR	**Active-placebo:** week 30: reduced symptom score.	**Active-placebo:** combined sympt-med score
										**Change from initial (wks 0-3) to end (wks 37-40) in the active group:** Decrease in symptoms and symptom-med score.	
Mösges, 2010 [27]	6–14	27/27	0/0	Tree pollen drops	Updosing	30-90-150-300 IR each 30 min	NS	Asthma	Stal	Not an efficacy trial	No difference in PFR change between the active and placebo groups during updosing and no serious AEs.
Halken 2010 [7] (additional data to Wahn 2009 [3])	5–17	TOTAL 278 : 131/135		Grass tablet	6 mo	25 mcg Phl p 5 daily (300IR)		RC	Stal	**Active-placebo:** Total symptom score reduced over whole season and at peak pollen season. Nasal and ocular symptoms reduced. Rescue medication used less during whole and peak pollen season.	None
Nieminen 2010 [8] (subgroup of study Valovirta 2006 [6])	5–15	10 low 10 high 10 Plac		Birch-alder-hazel mix, drops	2 y	24,000 SQ-U/wk (3.6 mcg group 1), 200,000 SQ-U/wk (30 mcg grp 1)	0.5 and 4.5	RC(A)	ALK	Mechanistic study: **Patients with elevated symptom and medication score:** increase in allergen-induced PBMC mRNA IL-17 expression;	
										a positive and dose-dependent correlation SMS and IL-17 production. **High-dose group vs placebo at 2 y:** increase in FOXP3 mRNA expression. FOXP3 mRNA changes correlate with IL-10 and TGF-beta mRNA.	
Stelmach 2009 [15]	6–17	20/15	5/10	Grass, drops	pre-co for 2 y	10 mcg group 5 grass drops daily	NS	A	Stal	SLIT vs Plac: asthma symptoms, nasal symptoms, nasal+asthma symptoms, medication score, nasal+asthma+med score.	SLIT vs Plac: ocular symptoms, total Asthma+nose+eye symptoms
***Randomized controlled***											
Keet 2012 [21]	6–17	SLIT 10	0	Milk protein drops	14 mo	SLIT 7 mg, OIT-A 2000 mg, OIT-B 1000 mg milk protein daily	NS	CM		Food challenge passed by more OIT pts vs SLIT alone (SLIT 1, SLIT/OITB 6, SLIT/OITA 8)	3 of 6 desensitized OITB pts, 3 of 8 OITA pts regained hyperreactivity after 6 wk milk avoidance
		SLIT start then:									
		OIT-A 10 OIT-B									
		10	0								
			0								
Pajno 2011 [23]	8–16	Cont/coseasonal: 40/40	3/5	Grass drops	Cont: 3 y	8 mcg group 5, 5 times/week	NS	RA	Stal	Continuous vs coseasonal: 1^st^ year: symptoms+med, symptoms, chest symptoms, and med scores improved more with continuous SLIT.	3^rd^ year: no difference in clinical outcomes between continuous vs coseasonal SLIT
					Coseasonal: 3 × 4 mo						
Keles 2011 [24]	5–12	SCIT 15	4	HDM	18 mo	SCIT: 13 mcg Der p+f 1/mo	0.75	A (and R)	ALK	**Active vs pharmacotherapy:** SCIT→SLIT: all clinical parameters improved at 12 mo, half at 4 mo. SCIT: all but rhinitis score improved at 12 mo. SLIT: only asthma med improved at 12 mo.	SLIT vs pharmacotherapy: only asthma med improved.
				SCIT: alum adsorbed, SLIT drops							
		SLIT 15	2								
						SLIT: 0.75 mcg Der p+f 1 3 times/week					
		build-up SCIT	1								Pharmacotherapy: no clinical parameters improved
		then SLIT 15									
	
		Pharmacotherapy									
		15	3								
										**Within group:** Asthma medication and asthma attacks reduced at 4, 12, 18 mo compared to baseline with SCIT and SCIT→SLIT, reduced at 12 mo with SLIT.	
Marogna 2011 [121]	5–17	SLIT 34/ Cetirizine 34	3/4	HDM (not specified drop-tab)	36 mo	1000 AU 1/w	?	R2+A	Lofarma	**SLIT non-smoking:** clinical scores, nasal CS, B2 use, and pulmonary function tests all improved	**Cetirizine + non-smoking:** clinical and pulmonary function tests
		50% of each group: passive cigarette smoke*									
										**SLIT smoking:** all showed a trend to improvement, but only MEF25 was statistically significant.	
										**Cetirizine + Smoking:** all parameters worsened	
Pozzan 2010 [28], low quality, only once per year evaluation by patient	10–65	SLIT 34, Control 18	1/0	*Alternaria* drops	36 mo	1 dosis SLITone daily	?	R (A)	ALK	**Active vs control:** Symptom score reduced, med score reduced	**Active vs control:** No med score reduction
										**Active pre-post:** med score reduced	
Eifan 2010 [14]	5–10 y	SLIT16, SCIT16, Pharma 16	1/2	HDM SCIT: alum adsorbed, SLIT drops	12 mo	Dosing not clear (SLIT: 3.8 mcg Der p+f 1 3 times/wk	2.2?	A (R)	ALK	**SLIT and SCIT vs pharmacotherapy:** total rhinitis symptoms, asthma symptoms, medication, and VAS score.	**SLIT vs SCIT:** no difference in total rhinitis symptoms, asthma symptoms, medication, or VAS score.
						SCIT: 22.2 mcg Der p+f 1/m)					
***Open controlled, no randomization***											
Aquistapace 2009 [29]	6–18	cases 90/control81	NA	Several, drops	2 y	varied	NS	RC(A)	ALK (SLITone)	SLIT vs controls: reduced symptoms, med score, new sensitizations.	SLIT vs control: asthma symptoms
***Observational, prospective***											
Lee 2011 [13]	mean 14.7 (4–53)	Mono-sensitized 70 Multi 64	NS	HDM, drops	12 mo	5 drops 1000 STU/mL Dpt-Df 3/week	NS	R	ALK	none	Mono- and multisensitized symptom and medication scores: all improved. No difference between any variable.
Roger 2011 [22]	4–15 (total subjects 4–64)	122 (total n = 218)	none	HDM, drops	Updosing	Every 30 min: 30-60-120-240IR	30	R and/or A	Stal	8 systemic reactions (3 moderate), all continued SLIT. Higher frequency of AEs in asthmatic patients.	No difference in frequency or severity of AEs in patients under 15 y.
***Observational, retrospective***											
Trebuchon, 2012 [18]	735 pts 5–18 (1289 pts total)	No active or control groups	No active or control groups	HDM, drops	2+ y	Variable, most 300IR daily	No comparison possible	Resp. allergy	Stal, some ALK	Descriptive study of how SLIT is given, dosing schedules, duration, etc. Treatment ‘(very) effective’, according to physician: 82%. Reduction in asthma medication: 26% stopped taking ICS.	

*parental smoking (at least 20 cigarettes per day).

*Abbreviations:* AE, adverse event, A/P, active/placebo; NS, not signficant; HDM, house dust mite; Cont, continuous; plac, placebo; pre-co, pre-coseasonal; QoL, quality of life; TU, therapeutic units; AU, area under the curve; med(s), medication(s); OIT, oral immunotherapy; AE, adverse event; VAS, visual analog scale; pts, patients; PFR, peak flow rate; SAE, serious adverse event; RC, rhinoconjunctivitis; RCA, rhinoconjunctivitis and (mild) asthma; [I]CS, [inhaled] corticosteroid; STAL, Stallergenes; GRE, Greer; ALK, ALK-Abelló; TOR, Torii Pharmaceutical, Japan.

### Pediatric SLIT and allergic rhinitis

The efficacy of grass pollen SLIT tablets for seasonal allergic rhinitis in children had already been established in the first WAO SLIT position paper, based primarily on the results of 3 large trials done in Europe [124,125,128]. Two of these trials [125,128] had an extension published within the time frame reviewed here: one of them included an analysis of subgroups [129], and the other showed mechanistic data [25]. Halken et al. [129] showed that the 5-grass tablet was just as effective in school children (5–11 y) as in adolescents (12–17 y), and there was enhanced efficacy during the peak pollen season. The tablets were also highly effective if eye symptoms were evaluated separately.

A review of SLIT trials performed in the United States (US) was recently published [130]. It highlighted one new high-quality trial conducted among American children with grass-pollen allergic rhinoconjunctivitis [40] as well as another randomized, double-blind, placebo-controlled (RDBPC) trial conducted with a highly concentrated grass pollen sublingual solution [54].

There are several other SLIT studies with grass pollen that do not add much to the already accepted profile mentioned above, and most of them are of lower quality. However, some of them explored new aspects of SLIT. A- HDM SLIT study that recruited patients from primary care practices reported no effect of 2 years of treatment [53]. However, the clinical trial had several flaws (see Chapter 4) and is rated GRADE 2. A seemingly positive HDM SLIT trial was conducted in Japan, but the scientific quality of this study was GRADE 1 [66].

### Pediatric SLIT and asthma

There were 2 randomized trials directly studying SLIT in allergic asthma. Eifan et al. demonstrated that 1 year of treatment with HDM SLIT was more effective than pharmacotherapy in controlling asthma symptoms, medication, and visual analog scale (VAS) scores [27]. Bronchial- and nasal-specific hyper-reactivity were also reduced. No difference was shown between the SLIT group and a third group treated with SCIT; however, because the study was underpowered, no statement can be made about the SLIT–SCIT comparison. Grass pollen extract SLIT strongly improved asthma symptoms and medication scores in comparison to a blinded placebo, but the study quality was low due to a 40% dropout rate in the placebo group [131]. SLIT trials in asthmatic patients generally allow for the administration of maintenance and/or as-needed medication; thus, SLIT must not be suggested as monotherapy for treating asthma.

### Allergic respiratory disease: asthma and rhinitis

Four studies investigated SLIT in allergic respiratory conditions in children with both allergic rhinitis and asthma [47,62,132,133]. Three used HDM and one used grass extracts. The SCIT versus SLIT results of the HDM trial by Yukselen et al. [62] are discussed in more detail in the next section. Here, the SLIT versus placebo comparison was not significant on most parameters, but there were only 10 children in each group. Details of the other trials can be found in the table.

### SCIT versus SLIT for respiratory allergy

Yukselen et al. [62] conducted an RDBPC comparison between HDM SCIT and SLIT with a double-blind double-dummy design and concluded that both active treatments are effective, SCIT more so than SLIT. Although the study was - underpowered, with 10 patients in each of the 4 groups, the tendency was clear: both treatments improved rhinitis and asthma symptoms and medication scores, but the changes only reached statistical significance in comparison to placebo in the SCIT group. Also, the response to nasal challenge with HDM improved in both groups, but the bronchial HDM challenge dose improved only in the SCIT group, accompanied by a reduction in the post-challenge eosinophil count in broncho-alveolar lavage. Similar immunologic changes (HDM-IgE reduction, IL-10 rise) were seen after both SCIT and SLIT, with the exception of HDM-IgG4, which only rose in the SCIT group, reaching a statistically significant difference with the SLIT group [62].

A similar study was conducted by Eifan et al. [27]. In a randomized trial, they studied SLIT versus SCIT versus pharmacotherapy alone. Although the study groups were somewhat larger, the study was still underpowered, and no difference between SCIT and SLIT was shown in any of the many parameters examined.

### Food allergy

Early work in food SLIT suggests some benefit in terms of desensitization, but this has been limited to a few small trials. A small US study investigated SLIT for peanut-allergic children in an RDBPC trial [134]. Data suggested that SLIT could induce desensitization, but long-term studies are needed to see if tolerance will develop. Keet et al. [135] compared the efficacy of oral immunotherapy (OIT) versus SLIT for cow’s milk allergy in children. This study showed that OIT was superior to SLIT alone at inducing desensitization to cow’s milk, but desensitization was lost as early as 1 week off therapy.

### New insights

Research published since 2009 has provided some interesting new insights into the effects of SLIT.

• Age: 2 of the rhinitis studies [54,66], 1 also including asthmatic children [66], and 1 safety trial [97] included children ≥4 years old, and 1 food-allergy trial included children ≥1 year old [134]. Therefore, we now have medium-quality preliminary evidence of SLIT efficacy for rhinitis in children from 4 years of age [54].

• Two medium quality trials investigated continuous versus co- or pre-coseasonal grass SLIT administration:

○ A pre-coseasonal course of grass SLIT in drops over 2 consecutive seasons was compared with continuous administration of SLIT for 2 seasons and placebo in children with allergic rhinitis [47]. Although the study was underpowered to show intergroup differences, both active treatments reduced the combined symptoms and medication score statistically significantly better than placebo. Only the pre-coseasonal schedule reduced the medication score as well.

○ Pajno et al. demonstrated that 3 years of continuous or co-seasonal SLIT with grass pollen extract had different efficacy in children with seasonal asthma and rhinitis. At the end of 3 years, both treatments were equally efficacious in reducing total symptoms and lung symptoms and in inducing immunological changes, but during the first 2 years these changes were more pronounced for the continuously treated group [63].

• SCIT updosing for 4 months followed by SLIT maintenance turned out to be at least as effective as SCIT with regard to efficacy and immunological changes, but without the safety problems often seen with SCIT (see efficacy chapter for further in-depth discussion). In this study, 2 of 13 SCIT patients dropped out because of systemic adverse reactions) [24].

• A French group of investigators introduced a new efficacy variable, the adjusted symptom score (AdSS): by adjusting the rhinitis total symptom score for rescue medication use, the AdSS can estimate symptom severity and the treatment effect more accurately. Applying this variable post-hoc to published adult and pediatric trials, the investigators showed a reduction of the observed placebo effect [136].

• The deleterious effect of passive smoking in children with intermittent asthma and perennial HDM allergic rhinitis could be partially ameliorated by SLIT, although the improvement in β-2 use, pulmonary function tests, and nonspecific bronchial hyper-reactivity was more pronounced in the SLIT group without passive smoke exposure, as was the reduction in nasal eosinophils [132]. However, this needs to replicated as it was an open study, and it surely does not mean SLIT protects against passive smoke health effects.

• A very-low-quality study [66] indicated that mono- or polysensitized patients respond equally well to single-allergen SLIT.

• Adherence in pre-school children was promising in an Italian study [74].

### Unmet needs

A number of issues regarding the use of SLIT in children remain to be resolved:

• Dosing:

○ What is the optimal dose of allergens other than grass pollen in children?

○ What is the bioavailability of drops and tablets in children, and how will this affect the optimal dose?

○ Is efficacy retained in SLIT with multiple non-cross-reacting allergens?

○ What is the optimal duration of treatment needed to maintain long-term effects?

• Indications:

○ How efficacious is SLIT in children who are unresponsive to pharmacotherapy?

○ What is the long-term efficacy of SLIT?

○ Can SLIT prevent respiratory allergy in children with only eczema, or persistent asthma in children with rhinitis?

○ Can SLIT be used in children <4 years old?

• Other allergens

○ What are the safety, efficacy, and optimal dosing of SLIT for latex allergy?

○ What are the safety and efficacy of sublingual versus oral immunotherapy for food allergies, for example to milk, peanut, or hazelnut?

## Chapter 8: Definition of SLIT patient selection

• To be eligible for sublingual immunotherapy (SLIT), patients should have

○ History of symptoms related to allergen exposure documented positive allergen-specific IgE test.

• The allergen used for immunotherapy must be clinically relevant to the clinical history.

• A molecular allergy diagnosis provides further guidance for an appropriate SLIT prescription.

• Age does not appear to be a limitation.

• Single-allergen SLIT has been demonstrated to be effective in both monosensitized and polysensitized patients.

• Use of SLIT for latex allergy, atopic dermatitis, food allergy, and Hymenoptera venom is under investigation; more evidence is needed to support its clinical use for these indications.

• SLIT may be considered as initial treatment. Failure of pharmacological treatment is not an essential prerequisite for the use of SLIT.

• SLIT may be proposed as an early treatment in the therapeutic strategy for respiratory allergy.

• SLIT may be particularly indicated in the following patients:

○ Patients whose allergy is uncontrolled with optimal pharmacotherapy (that is, those with severe chronic upper airway disease).

○ Patients in whom pharmacotherapy induces undesirable side effects.

○ Patients who refuse injections.

○ Patients who do not want to be on constant or long-term pharmacotherapy.

### Molecular allergy diagnosis

The identification of disease-eliciting allergens is a prerequisite for accurate prescription of allergen-specific immunotherapy. Molecular allergy diagnosis (component-resolved diagnosis) represents a major advance for the selection of patients eligible for AIT.

Grass pollen is a major cause of respiratory allergy worldwide and contains a number of allergenic molecules, some of which (Phl p 1, Phl p 2, Phl p 5, and Phl p 6 from *Phleum pratense*, and their homologues in other grasses) are known to be major allergens [137].

In children treated with SLIT using a 5-grass pollen extract, sIgE and sIgG4 responses significantly increased to Phl p 1, Phl p 2, Phl p 5, and Phl p 6, but not to Phl p 7 or Phl p 12 [138]. This study confirms that the initial phase of SLIT with a grass pollen extract enhances sIgE synthesis and response to the same allergen components that induce IgE reactivity at natural exposure.

Recently Tripodi et al. [139] reported that IgE sensitization profiles to *P. pratense* are highly heterogeneous. When molecularly designed SLIT preparations are tailored to patients’ needs, this high heterogeneity should be taken into consideration and formulations should be driven by the allergens identified in locally performed population studies.

### SLIT in polysensitized patients

More data are needed to validate the efficacy of sublingual and subcutaneous multiallergen immunotherapy in clinical practice in polysensitized patients. Component-resolved diagnosis could improve the reliability of choosing particular molecular components of allergens for immunotherapy. This method provides specific information on the molecular component of allergens, confirming or excluding true sensitizations [140].

In large-scale clinical trials of grass pollen sublingual tablets, polysensitized patients benefited at least as much from allergen immunotherapy as did monosensitized patients [68]. However, in the “molecular allergy era,” monosensitization seems to be extremely difficult to identify, because the patient is in general sensitized to more than one component of each allergen [141].

The IgE response to an antigenically complex whole allergen extract includes antibodies to irrelevant molecules and to highly cross-reactive allergens. IgE epitopes may be limited to some allergenic molecules by abundance and perhaps solubility, and may also be affected by the potentially large variability between preparations of crude extracts.

The discrepancy between the information acquired using traditional diagnostic procedures and molecular diagnosis emphasizes the usefulness of component-resolved diagnosis, at least in areas of complex sensitization to pollens, in better determining the correct indication for allergy immunotherapy [142].

## Chapter 9: The future of immunotherapy in the community care setting

• The significance of primary care

○ The prevalence of allergic diseases is increasing rapidly worldwide; the point of first contact for most allergy patients is primary care.

○ Globally, allergic diseases are under-recognized and under- or misdiagnosed because the symptoms of IgE-mediated allergic disease (e.g., rhinitis, asthma, eczema, conjunctivitis) overlap with many other conditions.

○ The corollary is that allergic diseases are frequently treated inappropriately.

• Allergy education

○ Allergy teaching should become a core part of undergraduate and postgraduate curricula.

○ Primary care teams, in particular, require further training in the detection, diagnosis, management (including prevention), and treatment of allergic disorders.

○ Pragmatic programs need to be developed for a better patient-physician partnership.

• Delivery of SLIT in the community setting

○ Primary care physicians (PCP) and general practitioners (GPs) should know how to select the appropriate treatment for a patient’s illness and should be trained to make a comprehensive assessment and to recognize treatment failure (inadequate therapy, improperly administered therapy, inadequate control) and exacerbations of illness.

○ PCPs/GPs interested in treating allergic diseases with allergen immunotherapy (AIT) should be trained in all aspects of SLIT, including assessment of patients and administration of SLIT. Emphasis should be placed on detection and management of side effects, including local and systemic reactions.

○ Before SLIT therapy is devolved from allergists to primary care, carefully performed research to identify the risks, benefits, and cost-effectiveness of treatment will be required. This will be a requirement for commissioners, and without it, implementation is unlikely.

• Collaboration between primary care team and allergists

○ In order to control allergic diseases, it is essential to encourage and promote cooperation and collaboration between primary health care clinicians (including physicians, nurses, and others) and relevant specialists. Currently the status quo does not reflect this prerequisite for successful vertical integration of allergy care.

○ Primary health care clinicians should be able to administer SLIT under the mentorship of a trained allergist and maintain regular liaisons with the allergist.

○ In collaboration, the allergist and the PCP/GP will plan the SLIT, administer it to the patient, and arrange follow up as and when needed; they will also jointly decide when to discontinue therapy.

○ However, the decision whether or not to initiate SLIT (as for SCIT) should be made by the allergist.

### Introduction

Over the last 50 years or so, allergic diseases have increased to epidemic proportions globally, as clearly demonstrated in longitudinal population studies [143] with a concomitant rise in hospital admissions for severe disease [144]. Allergic diseases manifest in many different organ systems, often causing distressing and disabling symptoms for the sufferers and their families alike. These are currently managed suboptimally in the community setting [145,146], and because of their relative scarcity, allergy specialists are often difficult to access.

It is important that primary care physicians and general practitioners (PCP/GPs) working in the community have a clear understanding of allergy in order to differentiate allergic from non-allergic causes, such as sensitivity or intolerance, for which allergy medicines have limited effectiveness and for which there is no role for immunotherapy. However, H1-antihistamines (ideally second generation) and other agents may benefit the patient in conditions mimicking allergy (e.g., where pharmacological, hormonal, neurogenic, or other stimuli initiate direct degranulation of the mast cell, as for example with urticaria). Many symptoms of allergy can be managed with the judicious use of pharmacotherapy, but for some, particularly where medications are not effective or where very-long-term treatment is required, AIT offers the prospect of a cure. Subcutaneous immunotherapy (SCIT) was once available in the community but, in some countries, was withdrawn due to safety concerns (see Immunotherapy below). The advent of sublingual immunotherapy (SLIT) now offers the possibility of once again providing immunotherapy in the community setting.

### The language of allergy

Most patients with allergic diseases consult primary care physicians [147], but many people consult their primary health care teams with wide-ranging symptoms that may or may not be caused by allergy; the most common of these are rhinitis, asthma, and eczema. Allergy is a set of signs and symptoms triggered by release of chemical mediators from the degranulation of mast cells in response to crosslinking of IgE molecules bound to the membranes of these mast cells by an allergen. However, the term “allergy” is loosely used by both patients and health care clinicians, with patients ascribing many symptoms to an allergic cause when a carefully taken history reveals this is not the case [148,149].

Similarly lax use of the term by clinicians creates further misunderstanding and misdiagnosis, for example, describing the watering of eyes while cutting onions as an allergic reaction, or suggesting that the flushing of the face on exposure to strong sunlight is a “solar allergy”. We have a duty of care to our patients to attempt to make the correct diagnosis by taking a careful history and performing appropriate examinations and investigations [150]. Failure to meet patients’ needs leads them to seek help from alternative practitioners who may do more harm than good, and often at great expense to the patient.

### Educational needs

In many medical schools, the subject of allergy is not given a high priority or even included in the medical curriculum. This is compounded by the paucity of allergy education given to or acquired by those working in the community setting [151-153]. A description of those educational needs is beyond the scope of this statement but has been addressed elsewhere [154,155]. It is imperative that clear educational messages are made available to health care planners and the general public concerning what is, and is not, allergic disease and what treatments are and are not effective [156]. The educational needs of today will need to be revisited frequently in order to maintain relevance in a dynamic therapeutic field with an evolving system of language and definitions [157].

### Allergy management

Allergy management consists of a variety of strategies, foremost of which is avoidance of the offending allergen. This, of course, may not be possible, such as with the ubiquitous house dust mite (HDM) [158], but for other allergens, for example, peanuts or shellfish, avoidance is currently the only reasonable course of action. Many allergies can be managed by the judicious use of medications. For some diseases, such as rhinitis and asthma, there are clear guidelines for medication use, such as those from Allergic Rhinitis and its Impact on Asthma (ARIA) [159], Global Initiative on Asthma (GINA) [160], and the International Primary Care Respiratory Group (IPCRG) [161]. The need and priorities for research into best practices for diagnosis and management of allergy and asthma in primary care have been detailed by the IPCRG in a global Delphi exercise [162,163] and by the European Academy of Allergy & Clinical Immunology (EAACI) [164]. The importance of the PCP/GP for diagnosis and management of allergy in low- and middle-income countries has also been highlighted [165].

Rescue medications may be needed to treat some allergic conditions, for example, use of adrenaline in acute anaphylaxis or oral corticosteroids for an exacerbation of asthma or severe acute intermittent rhinitis. Similarly, routine medications such as antihistamines and intranasal steroids may provide adequate control of many allergic problems, such as urticaria or intermittent rhinitis.

### Immunotherapy

The use of allergen immunotherapy in the primary care setting [166-169] and the use of allergen extracts for the diagnosis of allergic disease [170,171] have been well documented. Before the mid-1980s many patients received SCIT in the community setting. Skin prick testing was used to assess patients before administration of allergen extract solutions. Anecdotally, many of these patients benefitted from this therapy, although it was delivered in a haphazard, random fashion with no true systematic evaluation. This resulted in a number of deaths and led to the abandonment of immunotherapy in primary care, coupled with a loss of confidence in this treatment modality, especially in the United Kingdom [172].

Recent research has shown that both SCIT [173,174] and SLIT [175-180] are effective treatments for allergic diseases. Given the significant burden these allergic diseases impose on the health care system, AIT appears to be a cost-effective adjunctive treatment in modifying the existing disease state [61,181]. SLIT represents an effective and well-tolerated treatment for seasonal allergic rhinoconjunctivitis in adults, but studies performed in children in primary care have not yielded the same degree of success as in adults for HDM allergy [53] or for grass pollen allergy [182]. Current ongoing pediatric trials and evaluation of long-term effects in adults will further define its role in therapy [183], but there is evidence that there is potential for early and significant cost savings in children with allergic rhinitis treated with immunotherapy; thus, immunotherapy could significantly reduce both allergic rhinitis–related morbidity and its economic burden [184].

For the near future, some forms of immunotherapy (e.g., Hymenoptera venom) will have to continue to be administered in specialist units because of the risk of anaphylaxis. Nonetheless, SLIT offers an effective [185,186], safe [86,187-189], and easy-to-use form of treatment that can be administered in primary care [167,190-192]. Fatal anaphylaxis to SLIT has not been documented, although local side effects are relatively common [193].

Because patients self-administer SLIT at home, there is considerable saving of time, both for the patient and of the primary care team, who only have to supervise the first dose, thus improving convenience and cost effectiveness for the patient [194-197].

There is now a wide range of allergens available for SLIT, such as grass [198] and HDM [199-205], and evidence for cumulative benefit is emerging [206,207]. Oral immunotherapy to food allergens, especially peanut allergens, is also a promising new use of this form of therapy [208].

The current challenge is to identify those patients who are most likely to benefit from the administration of SLIT, including discovering the steps necessary to identify likely candidates, the investigations needed to validate that choice, and the mechanisms needed to ensure efficient, effective, cost effective, and safe delivery of this new technology. One suggestion is to identify a subgroup of general practitioners who have a special interest in the field of allergic disease diagnosis and management. This new subgroup would receive a higher level of allergy training and would be provided with greater resources to assess and investigate patient needs, especially when access to allergy specialist care is difficult. Some title or credential would indicate that these GPs have reached level higher in the field of allergy than would be the case for most GPs [209]. For the immediate future, it would still be advisable that the decision of whether or not to initiate SLIT (as for SCIT) should be made by the allergist. The average GP currently has limited knowledge concerning SLIT [210].

Interested primary and secondary care organizations should work towards developing a framework that will lead to greater accessibility and availability of SLIT and improved education of patients and providers alike.

### Unmet needs

• Primary health care providers should learn to differentiate between allergic disease and symptoms with non-allergic causes such as respiratory viral infections and common, pharmacologically mediated reactions to foodstuffs, such as chilies and spices, which cause a runny nose and watery eyes.

• PCP/GPs should be educated about the local allergens in their areas of practice and their seasonal prevalence. This may include seasonal airborne allergens other than plant pollens.

• Primary health care clinicians should be able to use readily available pharmaceutical agents to ameliorate the symptoms of allergic rhinitis.

• Primary health clinicians, allergists, and other specialists who treat allergy-related illnesses, such as pulmonologists, otorhinolaryngologists, ophthalmologists, and dermatologists, should cooperate and collaborate to plan preventive and therapeutic measures.

• Primary health care clinicians need educational initiatives to help them to understand immunotherapy and, more importantly, to be able to recognize which patients might benefit from SLIT.

• Primary health care clinicians should collaborate with their specialist colleagues to develop care pathways to develop effective service delivery.

## Chapter 10: Methodology of clinical trials

• Allergen immunotherapy (AIT) requires specifically designed and sized trials that incorporate adequate methodology and interpretation.

• Subjects included in AIT trials should have experienced moderate to severe disease in previous years.

• Strategies to guarantee adequate allergen exposures and to avoid confounding factors require further development and implementation.

• The risk of unblinding due to side effects requires an analysis of the efficacy that takes into account the incidence of side effects in both the AIT and control groups.

• Standardized and validated primary endpoints that properly assess symptoms and medication usage are of paramount importance for improving the comparability of study results.

• The validation of a clinical minimal difference of the primary outcome and of a “responder” definition is crucial to discriminate improvements in real-life conditions.

• Secondary outcomes and surrogate markers do not replace the primary endpoint and can only provide additional information.

• Safety should be assessed using an universally accepted system to grade and classify adverse events.

• Study duration should be based on the type of efficacy being studied: treatment of allergic symptoms, sustained clinical effect, long-term efficacy and disease-modifying effect, or curative effect.

• Owing to variations in allergen content and formulations between extracts, appropriate SLIT dose-finding studies should be carried out for each product.

• Allergen challenge chambers provide a promising tool for evaluating the therapeutic effects of AIT in phase 2 trials, but additional studies are needed for comparison with natural pollen exposure.

• Large studies with standardized procedures investigating short- and long-term protection against food allergy, atopic dermatitis, and latex allergy are needed.

• Better adherence to the CONSORT criteria is needed to improve the quality of reporting of AIT trials.

### Introduction

#### Quality of clinical trials

The use of appropriate methodology in randomized clinical trials (RCTs) is essential for the critical assessment and registration of therapeutic interventions. In a recent study assessing reports of SLIT RCTs, only 4.2% met all of the criteria of the CONSORT statement [211], although a CONSORT statement checklist for conducting and reporting trials in allergen immunotherapy (AIT) has been published [212].

#### Methodological aspects of meta-analyses

Meta-analysis is a powerful tool for evaluating the efficacy of a therapeutic intervention, and meta-analyses have clearly demonstrated that AIT is effective overall for treating allergic rhinitis and asthma [60]. However, the methodological quality of systematic reviews of SLIT could be improved by including more details about the search strategy used, employing measures to avoid selection bias, assessing the methodological quality of the included studies [213], increasing transparency on sources of funding, and considering heterogeneity before pooling data [214].

#### Differences in performing clinical trials for AIT and pharmacotherapy

AIT and pharmacotherapy should be evaluated in separate, specifically designed RCTs. The results of AIT and drug trials should not be directly compared, because the characteristics of the studies and their settings are fundamentally different, including the severity and persistence of allergic disease in the patients enrolled, the issue of placebo, the allergen exposure, the level and severity of symptoms of placebo-treated patients, the clinical relevance of the efficacy of AIT, the need for validated combined symptom-medication scores, the differences between children and adults, and pharmaco-economic analyses [215]. A major difference between clinical studies testing symptomatic medication and pollen AIT is that symptomatic medication is evaluated during the pollen season when patients have peak levels of symptoms, whereas AIT starts months before the beginning of the pollen season, when patients are still asymptomatic and the intensity of the pollen season and the consequent symptom level are unknown. Therefore, if the intensity of the pollen season and the level of disease activity is low for a subset of the patient population in AIT studies, the efficacy of AIT can be underestimated, because a certain level of symptoms is necessary to demonstrate the activity of any therapy. In addition, AIT studies allow the use of rescue medication, further interfering with the assessment [216]. These reasons explain some of the low level of efficacy (20%–30% improvement over placebo) seen in AIT RCTs as compared with medication RCTs when mean symptom-medication scores are analyzed.

A joint effort between allergists, methodologists, regulators, patient groups, and the allergen manufacturers to address important research questions is desirable to obtain answers as rapidly as possible [215].

### Diseases and allergens to be investigated

Ongoing studies are evaluating the role of SLIT in food allergy and oral allergy syndrome. SLIT studies specifically for milk [135] and peanut [134,217] have shown clinical efficacy and safety. SLIT appears to be less effective, but also less burdened by side effects and systemic reactions, than oral immunotherapy. Its short- and long-term protection, however, remain to be determined in larger controlled studies with standardized procedures for allergen dosing, schedules, and duration [218].

In recent years, SLIT with inhalant allergens has been used to treat oral allergic syndromes induced by foods [219-221]. Larger and more rigorous studies are needed to confirm its efficacy.

Although previous studies have suggested a possible application of SLIT to atopic dermatitis in particular subgroups of patients with specific disease severity and sensitization profiles, the value of SLIT in atopic dermatitis still remains unclear because of limited research in this field, much of which has been of inadequate methodological quality and has produced controversial findings [222].

Further studies are needed to evaluate SLIT for latex allergy. A recent prospective, observational, open, case-control study provided encouraging results in children [223], but was not confirmed in a randomized double-blind placebo-controlled (RDBPC) trial in adults [224].

### Quality and standardization of allergen vaccines

Although the standardization of allergen extracts is important for proper clinical dosing and efficacy, variability in the biological potency of some allergen extracts has been described [225].

Manufacturers in Europe have implemented extensive protocols for standardization and quality control, but each company uses its own in-house reference materials and its own unique units to express potencies [226], making the doses currently used for SLIT rather difficult to evaluate. In addition, the potency of some European allergen extracts differs markedly from standardized allergenic extract preparations licensed in the US [227]. However, because the effectiveness of SLIT also depends on additional factors apart from the exact dose (e.g., qualitative composition, presence of different adjuvant molecules, bioavailability, route of administration), appropriate SLIT dose-finding studies should be done for each product [56].

The CREATE project has provided a major step forward in allergen standardization and provides a model for the development of a comprehensive panel of international reference preparations that will harmonize allergen measurements worldwide [228].

### Placebo

Appropriate placebos for SLIT are needed to preserve the blinding of studies in the absence of local side effects, and any analysis of efficacy should take into account the incidence of side effects in both the SLIT and control groups [215]. An ‘active allergen placebo’ approach has been suggested as an alternative [215], wherein patients with dual sensitivities would be treated with active SLIT for one and a placebo for the other.

The problem of unblinding of subjects due to side effects was raised to argue against the efficacy of SLIT observed in recently concluded large clinical trials [229]. However, side effects were not infrequent in the placebo groups, and the treatment effect was still clearly observed when only groups with comparable local reactions were compared [230]. An approach to verifying the hypothesis of positive bias due to the patients’ recognition of the active treatment would be to assess the effect of dose on the relationship between the occurrence or frequency of side effects and the observed efficacy [231].

Finally, the placebo arm should be considered differently in AIT and allergy medication RCTs, because placebo-controlled studies of SLIT permit free access to usual anti-allergic drugs [215].

### Selection of patients

There is a general perception of somewhat lower efficacy results for AIT therapies compared with symptomatic therapies, owing to some confounding factors such as the inclusion of highly symptomatic patients and the allowed use of reliever medications in AIT studies. However, the analysis of AIT studies shows higher clinical efficacy in highly symptomatic patients. A more accurate comparison based on the high-symptom tertile from AIT studies shows that the relative improvement in the combined symptom score between active and placebo groups ranged from 27% to 37% [216], beyond what is normally reported for symptomatic treatments such as antihistamines (7%) and nasal corticosteroids (18%) [232] and the standard set for minimal clinically relevant efficacy (20%) by a WAO task force [233].

Ideally, subjects included in AIT trials should have experienced moderate to severe disease in previous years [215]. Registration studies should enroll patients who are not taking regular medications for the treatment of allergic diseases in order to assess the magnitude of the treatment effect. However, other studies may be needed to investigate whether AIT is effective in patients whose allergies are not controlled by medications administered at recommended doses. The concept of severe chronic upper airway disease (SCUAD) has been proposed to indicate patients whose allergic rhinitis is uncontrolled despite adequate guideline-based pharmacologic treatment [215].

Subtypes of allergic disease can be characterized in terms of severity, activity, control, and responsiveness to treatment, and these phenotypes can also be used to predict disease severity, progression, and response to treatment. A uniform definition of severe allergic diseases is expected to help in clinical practice, research and epidemiology, public health, education, registration of medicines and reimbursement, personalized medicine, and the development of novel therapies [234].

### Design of clinical trials

#### Phase 2 studies

In past clinical experience, allergen doses were frequently adapted to the individual patient; thus, there are few data on the dose-response relationship of SLIT. Allergen products for SLIT are increasingly being required to conform to regulatory requirements for human medicines, which include the need to demonstrate dose-dependent effects. A recent document, produced by a Task Force of the EAACI Immunotherapy Interest Group, evaluated the currently available data on dose-response relationships in AIT and provided recommendations for the design of future studies [56]. Evidence about dose-response effects on efficacy and immunological outcomes is available from individual SLIT studies using native allergen extracts. Information about the dose-response effect on safety will benefit from future guidelines on the grading and reporting of adverse events, especially local reactions [235].

Owing to variations in allergen content and formulations between products from individual manufacturers and in the choice of endpoints, no comparison between studies and general dosing recommendations can currently be made. As a consequence, SLIT preparations should be studied individually in rigorous phase 2 dose-finding studies, in parallel with studies using surrogate antigen challenges in the eye and nose [235].

No definitive biomarkers predictive of a clinical response to SLIT are currently available. Candidate markers are facilitated allergen binding (FAB) inhibition, functional assays of inhibitory IgG4 and IgE-blocking factor, and basophil sensitivity [38,236], but further investigation is required.

#### Phase 3 studies

##### Baseline assessment

Most large AIT RCTs require participants to have had moderate to severe symptoms in previous years. To achieve this, one approach would be to start a 1-year placebo trial to select patients with sufficiently severe disease for enrollment; however, pollen seasons are highly variable between years and areas, and a run-in period of 1 season before starting AIT is therefore not feasible. Patients’ medical allergy history data are used in most RCTs, but they may not be reliable, because patients are likely to recall the most severe symptoms but not the average symptoms from the previous season.

Nonetheless, many studies have found that the majority of patients consulting allergy practices have moderate to severe intermittent or persistent rhinitis, and thus it is likely that most patients enrolled in AIT RCTs by allergy specialists have moderate to severe symptoms [215].

##### Randomized clinical trials in rhinoconjunctivitis

*Assessment of Allergen Exposure.* The level of pollen exposure should be taken into account when interpreting symptom scores in studies of AIT to treat pollen allergies. There is controversy about the correlation between pollen counts and the degree of clinical symptoms, with some studies showing a relationship [237,238] and others failing to show a connection [239]. The missing correlation may be due to numerous factors, including variations in allergen potency, weather changes such as rain, the presence of pollens outside of the pollen season, patients traveling in and out of pollen areas, heterogeneous pollen sampling and definitions of the pollen interval or threshold, and simultaneous exposure to other aeroallergens and irritants. Confounding elements that can affect clinical symptoms due to house dust mites are polysensitization, in particular to pets, and allergen avoidance measures because, although not clearly effective, they may be beneficial to some subjects [215,240-242].

In addition, the number of pollen grains needed to elicit symptoms is not well defined; therefore, a priori exclusion of centers in which the level of pollen is too low is not an acceptable approach. Using individual samplers to measure pollen and evaluating the effect of AIT relative to these measurements may solve this problem [243], although this goal will be difficult to achieve.

The definition of a peak season based on symptom/medication scores of the placebo group rather than on pollen counts (“peak placebo symptom days”) has also been proposed as an innovative approach to the analysis of seasonal diaries after AIT [244,245]. This approach allows the local allergy burden to be determined at different centers and seems applicable for prospective and retrospective studies, but its use remains to be validated.

Phenology and the biomolecular identification of allergenic pollen may represent a new perspective for future aerobiological monitoring [215,246]. For indoor allergens, the assessments of allergen exposure by measuring major allergens in reservoir dust samples (bed, carpet, soft furnishings, and so forth) using monoclonal antibody–based enzyme-linked immunosorbent assays might provide levels of individual exposure [226].

*Primary Outcome Parameters.* Standardized and validated primary endpoints are of paramount importance to improving the comparability of study results. In real life, patients receive both SLIT and medications to optimally control symptoms. In clinical studies, the prohibition of rescue medication is unethical because the pollen season may be long and intense. Older studies often used the total symptom score and the total medication score individually for the primary efficacy analysis. Because the intake of rescue medication interferes with the assessment of symptom scores, the Committee for Medicinal Products for Human Use of the European Medicines Agency (EMA) has recommended the use of combined symptom–medication scores but has not specified a recommended instrument [247]. Different algorithms have been developed for calculating adjusted symptoms + medication scores, but none is universally accepted and to date only one has been standardized and formally validated by taking into account the global allergy severity, allergy-related consultations, and the (also validated) rhinitis quality of life to produce an “allergy control score” [248]. Another possible approach, described in detail in 2 post-hoc analyses, is the “adjusted symptom score,” which adjusts the symptoms according to the use of medication by calculating the last observation carried forward [136].

A validated clinical minimal difference of the primary outcome parameters in RCTs is still an unmet need [215]. The decrease in symptom scores should be high enough to clearly reduce allergy-related morbidity under “real life” conditions [215]. Beyond the statistical significance of changes emphasized by many trials, the clinical relevance of the studies lies in the ability of patients to discriminate between treatments that are and are not effective [215]. The 20% relative improvement with respect to placebo proposed by WAO addresses this issue [233]. A further approach based on a “modified responder analysis” found that a cutoff of 30% optimally discriminated between active and placebo treatments during the entire season [249].

*Secondary outcome parameters.* A wide range of secondary outcomes are used in clinical trials, but few are validated or standardized. They include individual symptoms, quality of life and patient-reported outcomes [250,251], visual analog scales, and symptom-free or “well days” (described by EMA as “days without intake of rescue medication and a symptom score below a predefined and clinically justified threshold”) [252]. The item “days with severe symptoms” (or worst days) has recently been introduced to describe the treatment effect on the most troublesome days in the pollen season [253].

Functional measures (conjunctival, bronchial, or nasal provocation tests; acoustic rhinometry; spirometry; and peak expiratory flow rate) and in vitro parameters (allergen-specific IgE and specific IgG levels) are useful to support the primary clinical outcome [252], but secondary outcomes and surrogate markers do not replace the primary endpoint and can only provide additional information.

*Exploratory outcome parameters.* Provocation tests (e.g., conjunctival, nasal, or bronchial provocation or allergen exposure in allergen challenge chambers) may be used as primary endpoints in early-stage, dose-ranging (phase 2) studies [247].

Nasal and eye provocation tests have been standardized and used in many studies. Allergen challenge chambers provide a promising tool for evaluating the therapeutic effects of AIT. This method overcomes some drawbacks of conventional clinical outdoor studies, such as the unpredictable levels of pollen, and facilitates both a controlled and reproducible pollen challenge using a predefined protocol, with acceptable sensitivity and specificity in the reproduction of outdoor conditions [19,43,254,255]. This technique has been largely used in studies with drugs, and single challenge tests cannot imitate the priming effect of repeated or continuous allergen exposures seen in real life, which are needed to test AIT. The provocation in allergen chambers might be a helpful substitute, especially in long-term studies over several years, for a pollen season with low pollen counts in which an evaluation of the response to natural exposure is not possible [247]. However, additional studies are needed to validate allergen challenge chambers more thoroughly compared to natural pollen exposure [19,256-258].

Reliable predictive surrogate markers or biomarkers are needed that correlate with real clinical endpoints and could lead to individually tailored immunotherapy treatment. Potential surrogate markers could include early and late skin reactions as well as immunological parameters such as IgE levels, IgG subclasses, mucosal IgA, lymphocyte subsets, cytokines, and local and systemic inflammatory markers, but most markers have had low reproducibility and poor correlation with the clinical outcome in clinical studies. Combined with the low availability of assays for these markers in daily practice and anticipated high costs, these results mean that further investigation is needed to identify an ideal surrogate marker [259].

*Study duration.* In SLIT trials, induction and maintenance schedules varied widely in dosing interval, treatment duration, and whether treatments were administered preseasonally, pre-coseasonally, or perennially. The optimal duration has not been investigated in clinical studies, but the main aim of AIT is a persistent effect due to changes in the immune system that can only be demonstrated in long-term studies. High-dose grass pollen SLIT has been shown to have a sustained effect [3,260], and 3 years of treatment provides a beneficial effect that lasts for a further 2 years after stopping treatment [4,120,261].

In general, according to the EMA [247], different claims for efficacy are possible depending on study duration. These include treatment of allergic symptoms (short-term clinical trials conducted to show efficacy in the first pollen season after start of AIT or to show efficacy in perennial allergies after some months of treatment), sustained clinical effect (maintenance of significant and clinically relevant efficacy during 2 to 3 treatment years), long-term efficacy and disease-modifying effect (sustained significant and clinically relevant efficacy in post-treatment years), and cure (sustained absence of allergic symptoms in post-treatment years). Long-term studies can also be planned to document effects on the prevention of asthma and the spread of sensitization. Studies intended to address more than one claim must be carefully preplanned to avoid methodological problems.

*Adherence to immunotherapy.* Adherence to prescriptions is crucial for all long-term treatments, and this is particularly true for SLIT, which is managed at home by the patients themselves. Available postmarketing studies indicate that the compliance with SLIT ranges from 50% to 95%, depending on age and on duration of treatment [262]. Nonetheless, these reports have an inherent limitation in that the observation itself can distort the results to some extent. Sales data provided by manufacturers show an alarming rate of SLIT discontinuation, approximately 90% at 3 years after the prescription is first written [263]. However, a German analysis of renewal prescription for SLIT showed that after 2 years 51% of subjects had at least one renewal prescription, which is partially reassuring [264].

The major issues influencing patient adherence to SLIT are thought to be the patient’s perception of clinical efficacy, cost, and side effects [265,266]. Among the reasons cited for discontinuing treatment, SLIT patients indicated efficacy concerns as the major basis for withdrawal [267]. Improvement of adherence is likely to be achieved by improving the patient information provided by prescribers [266,268,269] and reducing the interval between follow-up visits [269,270].

*Publication of the results.* As in other areas of medicine, the quality of reporting of most immunotherapy trials is low. According to a recent review, only 4.2% of SLIT RCTs met all the criteria of the CONSORT Statement, suggesting that the use of the CONSORT Statement should be further encouraged [211].

#### Randomized clinical trials in asthma

##### Studies in Children

Because AIT has an indication for treatment of children, AIT products should be tested for efficacy and safety in pediatric populations [247]. However, this is a vulnerable population with specific issues concerning the recording of symptoms, the use of rescue medications, safety, and acceptance, especially in very young patients. The efficacy of products for SLIT therefore has to be evaluated in special trials in the pediatric population and not in combined trials of children and adults or derived from extrapolations [247].

In Europe, a demonstration of long-term efficacy is required for the pediatric investigation plan (PIP) that must accompany applications for marketing authorization submitted to the EMA [235]. Although this approach is scientifically correct, it involves placebo medication for several years at an age when active AIT might be most effective, raising concerns about the ethics of such a strategy, as well as the feasibility [271].

In recent years, large RDBPC trials conducted in Europe [54,124,125] and the US [40] with grass pollen SLIT demonstrated efficacy and immunological effects in children and adolescents during the first treatment seasons, and safety was acceptable. The pediatric population is expected to best benefit from the preventive effects of AIT, leading to the question of when to start treatment to achieve maximum efficacy. SCIT has a relative contraindication in children less than 5 years of age because of the possible severity of adverse effects, but SLIT may have a better safety profile, as documented by post-marketing surveillance safety studies conducted in children under the age of 5 [104-107].

For all these reasons, more priority should be given to the development of studies on primary and secondary prevention using AIT.

##### Prevention studies

Allergic rhinoconjunctivitis is a risk factor for the development of asthma, and treatment of the underlying allergy may represent an attractive method of asthma prevention, mainly in the pediatric population. However, no regulatory guidance exists in this area.

An ongoing multinational RDBPC trial in 5- to 12-year-old children was first designed to assess the preventive effect of high-dose grass SLIT on asthma development, both during treatment and after the end of treatment [119]. A randomized multinational trial conducted by the Immune Tolerance Network is investigating the potential role of SLIT in the primary prevention of aeroallergen sensitization and asthma in children with atopic dermatitis at risk for developing asthma and atopy [119,272]. The outcomes of these preventive intervention studies will provide evidence as to whether or not SLIT can play a role in the primary or secondary prevention of atopic diseases.

##### Cost-effectiveness studies

The cost-effectiveness of immunotherapy depends on the duration of the clinical benefit of immunotherapy following treatment cessation and on the break-even point of cumulative costs between immunotherapy and pharmacotherapy [273].

For SLIT with grass pollen allergens, short- and medium-term effectiveness can be regarded as proven, as long as the indication is appropriate and no contraindications are present. With other seasonal allergens, such as tree pollen allergens, it can be an effective treatment option, but data are rather scarce. No consistent proof of effectiveness is available for SLIT with house dust mite allergens and other perennial allergens [274].

To provide definitive evidence of cost-effectiveness, there is a need for health economic studies based on high-quality prospective and long-term clinical studies comparing immunotherapy with pharmacotherapy in real-life practice and comparing SLIT with SCIT [273,275].

## Chapter 11: Guideline development: from evidence-based medicine to patients' views

• Guidelines should be evidence-based and lately also safety, patient preference and costs are taken into account in the development of recommendations.

• Local guidelines on allergen immunotherapy have now been developed in several different countries/regions of the world. Their content is briefly reviewed in this chapter.

• Immunotherapy –sublingual and subcutaneous- has been included as one of the treatment options in several guidelines on the management of allergic diseases (rhinitis, asthma, etc.)

• There are progressively more systematic reviews on sublingual immunotherapy that sustain guideline recommendations

• The quality of the manuscripts reporting clinical trials on which sublingual immunotherapy guidelines are based can still be improved, e.g. taking into account the CONSORT criteria.

Evidence-based medicine has become an essential component in the preparation of guidelines, in that it attempts to provide a logical, transparent, and applicable framework from which the quality and relevance of clinical studies may be assessed in an unbiased manner [276]. However, it has become clear that factors other than scientific evidence should contribute to recommendations in favor of or against certain actions in the medical treatment of patients. In the course of the past decade, new systems for guideline development and evaluation have been developed, including the Grading of Recommendations Assessment, Development, and Evaluation (GRADE) system [277].

**Figure 4:**
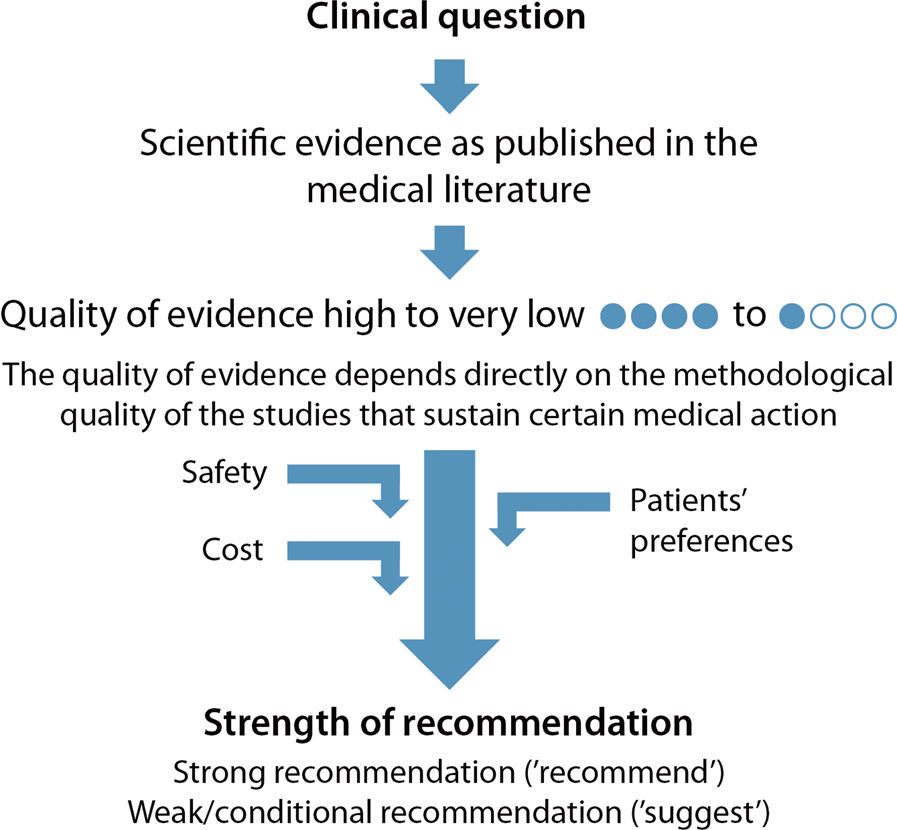
The GRADE system: from clinical question to recommendation.

The GRADE tool has been introduced as a method to support the use of clinical recommendations derived from analyses of different aspects of medical treatment in health policy decision making (Figure 4). Quality evidence from published research forms the basis of the guidelines, but safety, cost, and patients’ preferences are all considered in making the final recommendations. Tools to define the scientific quality of clinical trials that take into account internal and external validation and the risk of bias form an integral part of the GRADE system. In GRADE, all clinical trials—irrespective of their design—are considered, and their quality of evidence is established according to defined parameters (Table 9) [126]. Since 2004, the GRADE system has been adopted by many specialties as a useful tool for the formulation of guidelines.

**Table 9:** Evidence models used to establish guidelines for sublingual immunotherapy

	**Year**	**Evidence model**	No. of RCTs*	**Recommendation**
Specific guidelines on immunotherapy**				
WHO consensus [278]	1989	None	0	None
EAACI 1988 guidelines [279]	1988	None	0	None
EAACI 1992 [280]	1992	None	0	None
WHO Position Paper [281]	1998	None	2	None
EAACI Local	1998	None	4	Suggested in adults
Immunotherapy [282]				
AAAAI/ACAAI Practice parameters [283]	2007	Shekelle [284]	14	SLIT as investigational in US (no FDA approval yet)
**Canadian guidelines**[285]	**2006**	**None**	**10**	**SLIT evaluated positively as ‘novel form’, but no recommendation given**
**Argentinean guidelines**[286]	**2010**	**None**^**†**^	**No review**	**Indications on IT in general (AR, AC, Asthma) as co-treatment with medication. Extra indication for SLIT if SCIT is not tolerated/acceptable**
**AAAAI/ACAAI Practice parameters**[102]	**2011**	**Shekelle**[284]	**9**	**SLIT as investigational in US (No FDA approval yet)**
**British guidelines**[287]	**2011**	**SIGN**	**25**	**SLIT for adults and children with AR, after treatment failure with medication and avoidance.**
**Mexican guidelines**[288]	**2011**	**GRADE**	**18**	**Recommend SLIT for adults and children with AR and asthma; suggest for some cases of atopic dermatitis, latex allergy, and large local reactions to hymenoptera venom.**
**Chinese expert consensus on AIT for AR**[289]	**2011**	**Consensus**	**?**	**?**
**Finnish update on current care guidelines: AIT**[290]	**2012**	**?**	**?**	**Indicated for AR caused by grass pollen. Oral tolerance induction in children older than 5 y with severe food allergy.**
*Other guidelines in which immunotherapy is mentioned*				
ARIA 2001 [291]	2001	Shekelle [284]	12	Recommended in adults, suggested in children
ARIA Update 2008 [159]	2008	Shekelle [284]	36	Indicated in the same conditions as SCIT: patients with rhinitis/conjunctivitis/asthma caused by pollen or HDM; patients who have presented systemic reactions during SCIT.
**ARIA Update 2010**[292]	**2010**	**GRADE**	**63**	**Suggests the use of pollen and HDM SLIT for allergic rhinitis in adults and of pollen SLIT in children. Does not suggest HDM SLIT in children for treatment of AR. Suggests SLIT in patients with AR+Asthma for asthma treatment (low quality evidence).**
**GA**^**2 **^**LEN/EAACI pocket guide for AIT**[293]	**2010**	**Based on WAO IT papers and ARIA 2001, 2008, and 2010**	**No new review**	
**BSACI guidelines on Hymenoptera venom allergy**[294]	**2011**	**NICE accredited**	**0**	**SLIT for venom immunotherapy is mentioned as a future research area.**
**Guidelines for treatment of atopic eczema of the European Academy of Dermatology and Venereology**[295]	**2012**	**Appraisal of Guidelines Research and Evaluation and DELPHI procedure.**	**0 (this is a Review of Guidelines not RCTs)**	**Allergen IT (not stating SLIT or SCIT) to aeroallergens may be useful in selected cases of atopic eczema.**

*number of randomized controlled trials on SLIT the guidelines are based on.

**normal font: published in the original WAO SLIT position paper; **bold font:** new guidelines published since 2009.

^**†**^Table of evidence and recommendation taken from other guidelines based on Shekelle [284].

AC = allergic conjunctivitis; AIT, allergen specific immunotherapy; AR, allergic rhinitis; FDA, US Food and Drug Administration; HDM, house dust mite; IT, immunotherapy; NICE = National institute for Health and Care excellence, RCT, randomized controlled trial; SIGN = Scottish Intercollegiate Guidelines Network (SIGN) (http://www.sign.ac.uk/); SLIT, sublingual immunotherapy.

### Guidelines on immunotherapy

Since the publication of the original WAO sublingual immunotherapy (SLIT) paper [9], several new guidelines on allergen immunotherapy (AIT) have been published in which SLIT has been taken into account. Some of these new guidelines have adopted the GRADE system and its evidence model, whereas others have kept the Shekelle system (Table 9).

The Canadian Society generated a consensus paper on immunotherapy in 2006 [285] that evaluated SLIT positively as a novel form of allergen administration, but no clear recommendation was given. Similarly, the American Practice Parameters on Immunotherapy, 3^rd^ update [102], reviewed SLIT positively, but gave no recommendations for its use in the United States (US) because there is still no Food and Drug Administration–approved product on the US market. Even so, some US physicians do apply SLIT using the subcutaneous immunotherapy (SCIT) concentrated extracts to prepare the vials [296-298]. An expert panel of US allergists concluded that approved SLIT extracts will probably be on the US market in the near future, which should improve access to allergen immunotherapy for American allergy sufferers. However, the panel also raised some questions concerning how the efficacy/safety profile of SLIT compares to that of SCIT and how applicable a single grass allergen tablet will be in treating polysensitized patients, who represent the majority of individuals undergoing SCIT in the US [299].

The Argentinean Guidelines on Allergen Immunotherapy for the Prevention and Treatment of Respiratory Allergy in Childhood [286] analyze immunotherapy in general and include a table with grade of evidence and level of recommendation taken from other guidelines, without a specific new review of the literature. They recommend immunotherapy for allergic rhinitis, allergic conjunctivitis, and asthma as cotreatment with medication. The guidelines make a special note that SLIT is indicated in children who do not tolerate or accept injections.

The British guidelines on immunotherapy for allergic rhinitis state that both SCIT and SLIT are effective treatments for adults and children with severe allergic rhinitis who have failed to achieve sufficient relief of symptoms despite adequate medical treatment with antihistamines and intranasal corticosteroids and allergen avoidance. The guidelines conclude that there is category 1a evidence, according to Scottish Intercollegiate Guidelines Network (SIGN) grading for efficacy (http://www.sign.ac.uk/) in both adults and children and that the evidence is particularly strong for seasonal pollinosis. British experts state that SCIT and SLIT have been shown to give long-lasting benefit for some years after treatment is stopped. However, for asthmatic patients the risk/benefit balance is less favorable than for patients with rhinitis, and therefore immunotherapy for asthma is not routinely recommended in the United Kingdom. Further, the guideline states that the safety profile of SLIT appears to be superior to that of SCIT [287].

The Mexican guidelines include an in-depth analysis of the literature following the GRADE system. SLIT is recommended as an alternative to SCIT in children and adults with allergic rhinitis and/or asthma in whom AIT with pollen and/or house dust mite (HDM) is indicated, but who reject injection treatment or in whom there might be safety issues with SCIT. In Mexico, SLIT is gradually updosed over at least a month’s time. SLIT is also suggested for selected patients with atopic dermatitis and aeroallergen sensitization, latex allergy, or large local reactions secondary to Hymenoptera venom allergy [288].

The Subspecialty Group of Rhinology of the Chinese Society of Otorhinolaryngology Head, Neck Surgery that is part of the Chinese Medical Association wrote an expert consensus on AIT for allergic rhinitis [289]. As the full text is in Chinese we could not analyze the exact content of this document.

### Other guidelines in which immunotherapy is mentioned

There are several other related guidelines in which recommendations for immunotherapy are mentioned, although not all specify the sublingual route. The updated version of ARIA 2010 [292] includes the GRADE system for evaluation of the evidence. ARIA 2010 ‘suggests’ (that is, weakly recommends) the use of SLIT in adults with allergic rhinitis without asthma caused by pollen or HDM allergy and in children with allergic rhinitis caused by pollen allergy. If there is concomitant asthma, ARIA 2010 suggests that SLIT should be part of the integral treatment of the asthma. ARIA 2010 is based on publications up until 2009 and inevitably lacks the inclusion of several recent high-quality SLIT trials, which are likely to influence the next update.

The GA^2^ LEN/EAACI Pocket Guide for Allergen Immunotherapy for Allergic Rhinitis and Asthma [293] quotes the WAO and ARIA guidelines. It recommends considering AIT in patients with moderate to severe intermittent or persistent allergic rhinitis and in mild (controlled) allergic asthma proven to be caused by a well-defined allergen; it particularly recommends AIT in those who do not respond sufficiently to current pharmacological treatment. These guidelines state that independent studies show that both SCIT and SLIT are effective in allergic rhinitis and asthma if optimally used.

Other guidelines in which immunotherapy is mentioned are the British Society for Allergy and Clinical Immunology **(**BSACI) guidelines on Hymenoptera venom allergy [294] and the guidelines for treatment of atopic eczema of the European Academy of Dermatology and Venereology [295] (Table 9).

Italian investigators studied the congruence between international guidelines and AIT prescription patterns in 518 patients from 34 allergy centers. The investigation focused on the fact that ARIA and GINA guidelines recommend prescribing AIT according to disease severity. They concluded that in mite-allergic patients with rhinitis and asthma comorbidity, the severity of rhinitis and young age are the most important factors driving AIT prescriptions. The congruence of AIT prescriptions with guidelines was better for the ARIA (r = 0.87; *P* = 0.001) than for the GINA (*P* = 0.02) guidelines [300].

### Quality of SLIT meta-analyses and clinical trials

The 3 types of scientific sources used to develop the guidelines reviewed above are other guidelines, systematic reviews, and clinical trials. Although systematic reviews are considered one of the main pillars of evidence-based medicine, it has become clear that systematic reviews can be of varying quality, depending on the quality of the included trials and the rigor of the analytical method used. Cochrane meta-analyses, conducted under the auspices of the Cochrane Collaboration Group, must meet the highest quality standards. An updated Cochrane meta-analysis on SLIT for allergic rhinitis published by Radulovic et al. in 2011 included 42 randomized, double-blind, placebo-controlled trials. It showed that SLIT significantly reduced rhinitis symptoms and medication requirements [1].

The quality of the published manuscripts from clinical trials on allergen immunotherapy is not always optimal, as shown in a CONSORT analysis of publications on SCIT and SLIT trials [211]. Only a small percentage of SLIT trials met all CONSORT criteria. Because this is a tool for evaluating published data, it does not necessarily indicate poor quality of the trials themselves, but it does demonstrate that there is a need for improved quality of published documents. To facilitate this task, a CONSORT statement checklist in allergen-specific immunotherapy has been published [212].

### Conclusions and future needs

Since the publication of the original WAO SLIT paper [9], several new guidelines that focus on allergen immunotherapy have been published, as have guidelines that include statements concerning allergen immunotherapy. All reviews support the use of SLIT for treatment of seasonal allergic rhinitis in adults and children, whereas for perennial HDM allergy and in patients with bronchial asthma the data on SLIT efficacy are judged to be less robust. The majority of guidelines recognize SLIT to be safer than SCIT. However, there are 6 reports worldwide of severe systemic reactions to SLIT. Because SLIT is administered at home, patients should be educated on how to recognize and treat systemic reactions.

Two recent, long-term, large randomized controlled trials have provided evidence for the persistent long-term effect of SLIT for at least 1 [120] and 2 [4] years following its discontinuation. The possible preventive effect of SLIT on the development of asthma in children with allergic rhinitis is the primary aim of a large double-blind placebo-controlled study currently in progress (the GAP study) [119].

Systematic reviews and meta-analyses continue to represent the most robust forms of scientific evidence for efficacy, but they do have limitations. For example, systematic reviews are based on clinical trials that recruit highly selected patients, so that extrapolation to the whole allergic population may not always be justified. There is also the potential problem of negative selection bias in the published evidence and the potential for lack of complete reporting of harmful effects, which may skew the results of subsequent analyses. The recent evolution of ‘definitive,’ adequately powered, multicenter randomized controlled trials in both children and adults should minimize these concerns. Furthermore, both the European and American Academies of Allergy, Asthma, and Immunology have established surveillance studies of AIT to be completed by their members [71,301]. Data obtained in these real-life situations will be important for improving decision making for further guideline development.

### Unmet needs

• Immunotherapy guidelines should be based on up-to-date internationally recognized tools such as GRADE (Grading of Recommendations Assessment, Development and Evaluation), SIGN (Scottish Intercollegiate Guidelines Network), or NICE (= National institute for Health and Care excellence) to make them more robust.

• Recommendations given in immunotherapy guidelines should differentiate between and for different allergic diseases, adults versus children, and –in some cases- different allergen groups.

• It is of importance to make the latest evidence of SLIT more visible and accessible, so recommendations on the use of SLIT in Guidelines on the management of related allergic diseases can be based on the latest data.

• To adjust recommendations on SLIT use in Guidelines properly conducted studies on its effects on disease progression and prevention are crucial.

• A recently published standardized reporting system for local side effects associated with SLIT [76] should be used in future clinical trials, so results are more uniform and can be used for issuing the safety part of guidelines.

## Chapter 12: Practical aspects of schedules and dosages and counseling for adherence

• Extracts supplied by different manufacturers are still quantified by in-house reference materials with different (manufacturer-specific) units. As a consequence, a comparison of the potency of different allergy immunotherapy (AIT) products is not feasible.

• Standardization of materials and methods for determining the major allergen content of different AIT products would be preferable. A first approach in this direction has been made by the European Academy of Allergy and Clinical Immunology (EAACI) “CREATE” project.

• Adherence to sublingual immunotherapy (SLIT) is crucial for the effect size of this therapy. Real-life data from the SLIT European market reveal low levels of adherence.

• There is a clear need for improving adherence by systematically addressing it and its determinants and by putting more effort into educating patients, general practitioners (GPs), and specialists.

### Dosages

Although sublingual immunotherapy (SLIT) was first investigated more than 25 years ago in a double-blind placebo-controlled clinical trial [302], extracts supplied by different manufacturers for this (causal) therapy are still quantified by in-house reference materials with different manufacturer-specific units, such as “therapeutic units” (TU), “allergen units” (AU), and “index of reactivity” (IR) [303,304]. This makes it difficult or impossible for the prescribing allergist to compare different SLIT products with regard to the potency.

A review article by Larenas-Linnemann et al. summarized all available information as provided by the manufacturers on the monthly maintenance doses of different SLIT products [303]. The authors reported that these doses were 5 to 45 times higher than the doses for subcutaneous immunotherapy (SCIT) products from the same manufacturers. However, it was not possible to compare different SLIT products, because heterogeneity was found in the major allergen extracts of different products as well as in the enzyme-linked-immunosorbent assays (ELISAs) and antibody sera used for reference extracts. This led the authors to conclude that the “effective dose range [for SLIT] still is to be determined” [303].

As a consequence, it would be advisable to have standardized materials and methods for determining the allergen content of different SLIT products. A first approach in this direction has been made by the CREATE project for the development of certified reference materials for allergenic products and validation of methods for their quantification, which was initiated by the EAACI in the last decade [226,228]. The initial aim was to establish and validate assays and corresponding monoclonal (recombinant) antibodies for the quantification of the 2 most prevalent allergens, Bet v 1 and Phl p 5, in diagnostic and therapeutic extracts. Since then, this project has been pursued by the European Directorate for the Quality of Medicine & HealthCare (EDQM, http://www.edqm.eu). In November 2011, EDQM published valid data for the in vitro quantification of Bet v 1 in different extracts with the aim of having the European Pharmacopoeia adopt the assays and reference materials, which will become a reliable option for quantifying (and comparing) extracts in the near future.

### Amount of Phl p 5 and Bet v 1 allergens in SLIT products

In 2007, Sander et al. [304] investigated the daily amount of the major allergen Phl p 5 in 10 different grass pollen extracts produced by different European manufacturers by using validated ELISAs (Bradford assays, inhibition of IgE binding to *Phleum pratense* ImmunoCaps, and content of the main allergen by 2-site enzyme immunoassays). The authors concluded that the Phl p 5 content in daily doses of the SLIT products ranged from 0.2 to 21.6 μg in the extracts investigated (reviewed in [305]; Table 10).

**Table 10:** Daily doses of Phl p 5 in different SLIT preparations*

Manufacturer**	**Brand (concentration)**	**Daily doses of Phl p 5 (μg), analysis of Sander et al. 2009 [**[304]**]**	**Daily doses of Phl p 5 (μg), analysis of van den Hout et al. 2010 [**[306]**]**
Allergopharma	Allerslit forte	21.6	n.d.
	(715 000 SE/mL)		
Allergy-Therapeutics	Oralvac plus	0.6	n.d.
	(768 000 TU/mL)		
ALK-Abello	Grazax	5.0	5.0
	(75 000 SQ-T)		
ALK-Abello	SLIT one	0.2	1.4
	(1 000 STU/mL)		(SLIT one plus:
			2 500 STU/mL)
ARTU Biologicals	Igevac	7.8	n.d.
	(9 500 BE/mL)		
HAL Allergy	Sublivac B.E.S.T.	3.6	6.2
	(10 000 AU/mL)		(Sublivac FIX:
			10 000 AUN/mL)
Immunotek	Sulgen	0.9	n.d.
	(30 000 TU/mL)		
Laboratorios LETI	Tol SL	0.2	0.2
	(100 HEPL/mL)		
Stallergenes	Staloral 300	8.4	n.d.
	(300 IR/mL)		
Stallergenes	Oralair (300 IR)	n.d.	5.2

*Modified with permission from [304-306].

**Manufacturers are listed in alphabetic order.

HEPL, histamine equivalent prick leti; STU, standard treatment units; TU, therapeutic units; AU, allergen units; BE, biologische Einheiten; IR, index of reactivity; SQ-T, standardized quality units tablet; SE, sublinguale Einheiten; AUN, Allergy Units Native; n.d., not determined.

In 2009, the extracts were reanalyzed by the same working group [306]. In between, the amount of major allergen in 2 liquid SLIT products was modified, and 1 grass-pollen tablet product was measured for the first time because it had not been on the market at the time of the first analysis 2 years earlier. Some SLIT products on the market, such as allergoid tablets, were not included in the analysis. Importantly, the measured doses for all SLIT products were below the amounts published by manufacturers from their in-house assays [305].

The daily amount of the major allergen Bet v 1 in 4 birch SLIT products has been also analyzed ([307], reviewed in [305]; Table 11). The authors concluded that the Bet v 1 amount in the daily doses of investigated SLIT products ranged from 1.0 to 46.7 μg in the extracts investigated.

**Table 11:** Daily doses of Bet v 1 in 4 SLIT preparations*

Manufacturer**	**Brand (concentration)**	**Daily doses of Bet v 1 (μg), analysis of Kerkvliet et al. 2011 [**[307]**]**
ALK-Abello	SLIT one plus	5.8
	(2 500 STU/mL)	
HAL Allergy	Sublivac FIX	46.7
	(10 000 AUN/mL)	
Laboratorios LETI	TOL SL	1.0
	(100 HEPL/mL)	
Stallergenes	Staloral	25.4
	(300 IR/mL)	

*Modified with permission from [305,307].

**Manufacturers are listed in alphabetic order.

HEPL, histamine equivalent prick leti; STU, standard treatment units; IR, index of reactivity; AUN, Allergy Units Native.

Taken together, these heterogeneous data underline the importance of establishing standardized methods to evaluate the major allergen contents in different SLIT products, as proposed by the EDQM. This approach would make feasible a direct comparison of different SLIT products. However, one should keep in mind that using monoclonal “marker” antibodies would only give information on the corresponding major allergen in SLIT preparations. In products such as grass-pollen extracts with multiple dominating major allergens, information on the content of only one “marker” allergen, such as Phl p 5, would be feasible, which would, again, make it difficult to compare SLIT products.

### Dosing schedules for SLIT products on the market

The SLIT products on the market are accompanied by a high variety of recommendations on when treatment should begin and schedules regarding the updosing and maintenance phases (Table 12).

**Table 12:** Specific characteristics of some SLIT products on the market*

**Manufacturer**	**Brand**	**Initiation of therapy**	**Length of induction phase to reach maintenance dose**	**Maintenance therapy**
Allergopharma (manufacturer)	InfectoSlit Gräser (formerly distributed and marketed as Allerslit forte)	Preseasonal start of SLIT is recommended, then perennial therapy	Updosing on 1st day under guidance of physician	4 drops daily, approximately 3 minutes before swallowing, thereafter 30 minutes no drinking or eating
InfectoPharm (distributor)				
ALK-Abelló	SLIToneULTRA	Perennial start possible	SLIToneULTRA is provided in single-dose containers. Induction phase duration 5–10 days. Start with 50 SRU (Standardized Reactivity Units) for 5 days. Maintenance dose is 100 SRU, 150 SRU, or 300 SRU.	0.5 mL (one single-dose container) daily.
				Perennial intake.
ALK-Abelló	GRAZAX	Perennial start possible.	No updosing phase needed, Start with maintenance dose, first intake under guidance of physician	One grass pollen tablet daily, dissolves immediately under the tongue.
				Perennial intake.
Allergy Therapeutics	Oralvac Compact	Perennial start possible	(Classical) Updosing at home over 10 days (bottle 1 to 3)	Dosing with a pump:
				Daily 3 pumps of bottle 3,
				minimum of 1–2 minutes before swallowing
			or Updosing on 1st day under guidance of physician (bottle 3)	
HAL Allergy	Sublivac	Perennial start possible	Updosing at home over 5 days	5 drops daily, minimum of 1 minute, preferably 2–3 minutes before swallowing
Laboratorios LETI	TOL forte	Preseasonal perennial	Updosing at home. Maintenance dose reached in 2 days. Single concentration.	Dosing with a pump 2 pumps daily
Lofarma	LAIS Sublingual drops	Pre/coseasonal or perennial therapy	Updosing at home over 4 days	6 drops daily for 120 days minimum (pre/coseasonal treatment) or 6 drops twice per week (perennial treatment), Sublingual intake minimum of 1–2 minutes before swallowing
Lofarma	LAIS sublingual tablets	Pre/coseasonal or perennial therapy	Updosing at home over 4 days	1 tablet daily for 120 days minimum (pre/coseasonal treatment) or 2 tablets per week (perennial treatment) tablet dissolved under the tongue over a minimum of 1 minute
ROXALL	SULGEN Spray	Perennial start possible (also coseasonal)	Direct start with the maintenance dose at home (no updosing necessary)	2 spray doses daily, perlingual 2 minutes before swallowing
Stallergènes	Staloral	Preseasonal or coseasonal start, therapy interruption after season, optional perennial therapy	(Classical) Updosing at home over 11 days or Updosing on 1st day under guidance of physician (‘ultra-rush’)	8 pumps of bottle with highest concentration daily or optionally 4 pumps daily in perennial therapy
Stallergènes	Oralair	Preseasonal start (recommended 4 months before pollen season), coseasonal treatment, and then therapy interruption after season	Updosing at home over 3 days	One tablet (5-grass extract), dissolving over a minimum of 2 minutes

^*^Modified with permission from [308,309].

### Counseling for adherence

As for every chronic condition, adherence to the treatment is a critical issue. Rates of adherence in individual patients are usually reported as the percentage of the prescribed doses of medication actually taken over a specified period [310]. There is no consensus standard on what represents an acceptable adherence rate (which should, in principle, be 100%); however, as a convention it is considered that adherence greater than 80% is adequate for most treatments [310]. Several methods have been employed for measuring adherence, but none of them can be assumed as the gold standard.

So far, 6 observational studies have specifically addressed this issue [75,311-315]. Overall, 1879 patients were evaluated (848 adults, 154 adolescents, and 877 children). The follow-up duration ranged from 3 months to 3 years, and the average adherence was around 80%. In the so-called “big trials”, which involved more than 150 patients and were mainly performed to assess the clinical efficacy and safety of dissolving tablets, the adherence was reported to be around 90% (range 88%–94%) in both adults and children [183]. Although controlled studies and real life represent different situations, the evaluation of adherence in large trials can be taken as a useful model to predict the potential drawbacks of treatment in real life. However, the small number of patients evaluated in these studies should be taken into account.

In contrast, less encouraging data are coming from the SLIT European market. Two recent studies showed a significant decrease of prescription renewal rates over time [263,264]. According to these data, only 51% of patients in Germany and 30% in Italy stay on SLIT longer than 2 years. In another analysis from the German market the adherence to SLIT was even lower with only 16% after three years of SLIT [316]. A recently published comparison of adherence between SLIT and SCIT has confirmed a low rate of adherence for both routes of administration, even if higher for SCIT [317].

Factors affecting the adherence rate are important to consider. The cost is one of the most relevant, and this is clearly noticeable when comparing the sales of SLIT where the treatment is reimbursed or is paid entirely out of pocket by the patients [263]. Another important issue to consider is patient education and information about the clinical and immunologic benefit of specific AIT. Recently, a small study reported a significantly higher rate of adherence in patients who underwent short educational training than in a group that received only the instructions about SLIT administration [318]. In addition, because SLIT is self-managed at home without direct supervision, regular follow-up is mandatory. Vita and co-workers reported a relationship between the number of follow-up visits and the adherence to treatment [270]. In fact, the number of patients who withdrew from treatment was significantly lower in the group of patients who received 3 visits/year than in the group of patients who were seen at longer intervals. In another study, acceptable adherence was achieved by the use of an electronic device to remind patients to use SLIT regularly over a 10-week period [319].

However, the cost/benefit ratio of such an approach in the long term has to be confirmed. Reasons for poor adherence to SLIT have been addressed in a survey carried out among Italian allergists [265]: in addition to the need for education and regular follow-up, the survey highlights issues such as the tolerability and the treatment schedule. Tolerability plays a pivotal role. Adverse events, mostly local reactions, account for at least one-fourth of all dropouts in clinical trials and the rate is likely to be even higher in real-life setting [320]. In fact severity, persistence or simply poor awareness of local reactions may increase the risk of treatment discontinuation, despite its efficacy. Even the perception of allergen-specific immunotherapy among GPs can be relevant to the adherence to SLIT: In a different Italian survey, an 11-item questionnaire on AIT was emailed to 200 GPs. Although the attitude towards this therapy was generally favorable, fewer than 50% of GPs were aware that international guidelines include SLIT as a treatment option and provide suggestions for its use [210].

In conclusion, it seems that there is room for improving compliance by systematically addressing it and its determinants and by putting more effort into educating patients, GPs, and specialists.

## Chapter 13: Perspectives & new approaches

• Recombinant allergens can be considered the promising future of allergen immunotherapy (AIT). They are currently used in clinical practice for advanced allergy diagnosis and will possibly be used in the future for AIT.

• After the patent expires on a biological therapeutic product, similar products may emerge on the market. These products are not generics, but are rather defined as “biosimilars”. It will be critical to have AIT products in the category of biosimilars to preserve the quality of the treatments.

• Some sublingual immunotherapy (SLIT) preparations include adjuvants with the aim of amplifying the therapeutic effect by modulating the immune response or/and further improving the safety profile.

• Validity of single products should be reported in publications in order to avoid generalized and misleading judgments about AIT and to help regulatory authorities in evaluating specific products and clinicians in choosing scientifically supported immunotherapy products in their practices.

### Recombinant allergens

Modern technology has enabled the synthesis of recombinant proteins whose main advantages are their fully characterized physical, chemical, and immunologic properties. It has become possible to produce well-defined recombinant and synthetic allergy vaccines that can specifically target the mechanisms of the allergic disease [321]. These can be considered the promising future of AIT that will lead to personalized allergen immunotherapy. In accordance with the new regulatory framework, it is likely that the clinical development will be as recently proposed by Cromwell [322]: “*Personalized or patient-tailored immunotherapy is still a very distant prospect, but the first recombinant products based on single allergens or defined mixtures could reach the market within the next 5 years.”*

A more successful utilization of recombinant allergens is already present in clinical practice for advanced allergy diagnosis.

### Biosimilars

After the patent expires on a biological therapeutic product, similar products may emerge on the market. These products are not generics, but are rather defined as “biosimilars” by the major regulatory authorities worldwide [323]. One of the major differences between “generic” and “biosimilar” is that biosimilars must undergo clinical development for registration, including phase 3 studies, just as in the case of the parental drug. The process of registration of AIT products started just a few years ago in many countries.

Although the patent expiration is not an immediate problem, it is critical to have AIT products (which are biological and biotechnological products) in the category of biosimilars to preserve the quality of the treatments provided to allergic patients. In fact, biosimilars imply that specific research, clinical monitoring, and pharmacovigilance have occurred to produce a safe and appropriate use of these products.

### Adjuvants and modified allergens

Some SLIT preparations include adjuvants with the aim of amplifying the therapeutic effect by modulating the immune response or of further improving the safety profile. Adjuvants are substances that have the potential to enhance the immunogenicity of antigens or allergens and have largely been investigated for injected allergen immunotherapy. Oral dendritic cells may be the ideal target cells for adjuvanted SLIT, which could enhance the tolerance mediated by these cells and mimic the natural contact of the individual’s immune system to allergens [324].

Probiotics have been investigated as adjuvants for SLIT in mouse models and have resulted in enhanced induction of tolerance [325,326]. Another approach investigated the use of detoxified bacterial toxins or carbohydrate polymers adjuvanted to allergens as “microparticles” and mucoadhesive particles [324,327-329]. Encouraging preliminary clinical results in humans come from the first phase 1/2, dose-ranging, placebo-controlled clinical trial with monophosphoryl lipid A–adjuvanted SLIT [16]. The specific immunological mechanisms underlying the therapeutic potential of adjuvants and vector systems will be further investigated in upcoming trials.

Chemically modified allergen preparations suitable for sublingual administration have been obtained by carbamylation of the native allergen in order to maintain its molecular dimension [330]. These preparations have shown clinical efficacy and immunological effect in clinical trials [331,332]. Carbamylated allergoids have the advantage of reduced IgE-binding activity, which improves the safety profile, and enhanced bioavailability, as observed in pharmacokinetic studies [333].

These amplified features of SLIT products using adjuvants and modified allergens suggest that fine-tuning of doses and evaluation of the dose response is specifically required with respect to traditional SLIT preparations.

### Validity of single products

Meta-analyses have confirmed the effectiveness and safety of SLIT [1,185,334-336]. Nonetheless, they evaluated the available trials without considering differences between products, preparations, dosages, schedules, and sometimes allergens. Some scientists have called for AIT clinical trial publications to specify the specific brand name of the immunotherapy product used [337] for both positive and negative results. This is crucial to avoid generalized and misleading judgments about AIT and could help regulatory authorities to evaluate specific products and clinicians to choose scientifically supported allergen immunotherapy products in their practices.

## Chapter 14: Raising public awareness about sublingual immunotherapy

• Allergen immunotherapy (AIT) is effective in alleviating allergy symptoms to a similar or even greater extent than pharmacological treatments for both asthma and allergic rhinitis; nonetheless, awareness about AIT is still poor.

• Strategies to increase the awareness of AIT include the following:

○ Patient associations should partner with medical associations.

○ Collaboration between primary care physicians and allergists is essential.

○ Proper educational programs should be designed to increase knowledge about AIT.

○ Allergic diseases and AIT are still under-recognized or not recognized by regulatory authorities in many countries, although the numbers of allergic patients is increasing.

○ Harmonization among the regulations of different countries is needed.

○ Scientific societies should partner, at any level, to provide advice and promote this process.

○ Advocacy and education of government policy makers will be crucial to secure more resources for research on immunotherapy and similar preventative strategies.

Allergen immunotherapy (AIT) is effective in alleviating allergy symptoms to a similar or even greater extent than pharmacological treatments for both asthma and allergic rhinitis; it also has long-lasting effects that persist after the treatment is discontinued. Moreover, immunotherapy has been shown to prevent the progression of allergic diseases and to reduce the development of comorbidities and new sensitizations. Therefore, immunotherapy is currently the only medical intervention that could potentially alter the natural course of allergic diseases. Better-standardized extracts and improved techniques and routes of administration, like the introduction of sublingual immunotherapy (SLIT), have further enhanced the use of this treatment strategy. Increased awareness and appropriate knowledge of immunotherapy amongst patients and policy makers is essential for better accessibility, affordability, and sustainability of immunotherapy as a treatment for all eligible patients.

Patients receiving immunotherapy frequently have poor knowledge and unfounded expectations of various important aspects of their treatment. However, dissemination strategies of immunotherapy have shown that appropriate knowledge is crucial for better compliance and effectiveness of immunotherapy [181]. About a decade ago, a survey-based study documented that 39% of patients expected complete recovery from their allergies, 20% of patients had no idea about the timing of anticipated improvement, and 18% expected improvement to occur within days. Furthermore, 60% of patients were unaware of any specified appropriate treatment duration, 33% considered immunotherapy to be without side effects, and only 3.3% of patients correctly identified all of the allergens to which they were undergoing desensitization [338]. A more recent, cross-sectional, Italian multicenter survey in a large cohort treated with subcutaneous immunotherapy (SCIT) or SLIT found that, despite some gaps and misconceptions, the majority of patients had an adequate level of knowledge, perception, expectations, and satisfaction about AIT that was similar to the level of results expected by physicians [339]. In particular, most patients acquired their knowledge of AIT from their physician and perceived AIT to be safe and easy to integrate into a daily routine. The main motivations for the use of AIT were its potential effect to alter the natural course of the disease, the reduced need for anti-allergy drugs, or dissatisfaction with current pharmacotherapy. Of note, SLIT was frequently considered easy to take and without side effects. Consistent efforts to improve the awareness among the lay public and patients at a global level will be crucial for increasing patients’ knowledge about AIT, improving their compliance, and promoting the success of this therapeutic modality.

Recently, several societies have moved forward to increase the awareness of AIT amongst patients, including the World Allergy Organization and the European Academy of Allergy and Clinical Immunology [340,341] and expert groups [181] focused on AIT awareness and its implementation.

Major problematic issues that were identified by these organizations included the following:

• Underappreciation of the science supporting AIT.

• Increased complexity of and time needed to perform appropriate AIT studies, as compared with clinical trials of drugs.

• Absence of reimbursement for AIT in many countries.

• Limited availability of resources for AIT research and promotion.

### Strategies to increase the awareness of AIT

Efforts by patient organizations, general practitioners, non-allergist health care professionals, and pharmacists will be needed to increase the awareness of AIT among allergic patients. Campaigns should be targeted to patients as well as to policy makers.

The following can contribute to increasing awareness of AIT:

• Patient associations should partner with medical associations to help in disseminating knowledge and awareness of allergy diagnosis.

• Collaboration between primary care physicians and allergists is essential. Proper documentation and instructions from the prescribing allergist’s office as well as forms designed for complete and accurate documentation of therapy are vital components of safe administration.

• Proper educational programs should be designed to increase knowledge about AIT within the community.

• Although allergic diseases and AIT are under consideration by regulatory authorities in many countries, they are still under-recognized or not recognized at all in many other countries with increasing numbers of allergic patients.

• For better, uniform practice and introduction of immunotherapy, harmonization among the regulations of different countries is needed. Scientific societies should partner, at any level, to provide advice and promote this process.

• Advocacy and education of government policy makers will be crucial to secure more resources for research on immunotherapy and similar preventative strategies.

## Competing interests

GW Canonica declares he has received consulting fees from Allergy Therapeutics, ALK, Circassia, Hal, LoFarma, and Stallergenes. L Cox declares she has received consulting fees from Stallergenes. R Pawankar declares she has no competing interests. CE Baena-Cagnani declares he has no competing interests. M Blaiss declares he receives consulting fees from Merck and Circassia and speaker fees from Merck. S Bonini declares he receives fees for participation in Expert Board Meeting of ALK and Stallergenes. J Bousquet declares he has received honoraria for: scientific advisory boards – Actelion, Almirall, Meda, Merck, MSD, Novartis, Sanofi-Aventis, Takeda, Teva, Uriach; and for lectures during meetings – Almirall, AstraZeneca, Chiesi, GSK, Meda, Merck, MSD, Novartis, OM Pharma, Sanofi-Aventis, Schering Plough, Takeda, Teva, Uriach; and for Board of Directors for Stallergenes. He has received honoraria for participation in scientific and advisory boards, lectures, and press engagements from Actelion, Almirall, AstraZeneca, Chiesi, GlaxoSmithKline, Meda, Merck, Merck Sharpe & Dohme, Novartis, oM Pharma, Sanofi-Aventis, Schering Plough, Stallergenes, Takeda, Teva, and Uriach. M Calderón declares he has received consulting fees, honoraria for lectures and/or research funding from ALK, Stallergenes, Merck, Allergopharma, HAL, and Allergy Therapeutics. E Compalati declares he has received scientific consultancy grants for manuscript preparation by Stallergenes and Lofarma spa (not related to the preparation of the present position paper) and fees for some congress lectures. SR Durham declares he has received via Imperical College London consultancy fees from Merck, ALK Abello, Stallergenes, Circassia, Biomay, Boehringer Ingelheim, and lecture fees from Merck and GSK. R Gerth van Wijk declares he has received consultancy fees from ALK-Abello and HAL, and speaker fees from Allergopharma, and research funding from ALK. D Larenas-Linnemann declares she receives speaker fees from MSD, ALK; travel grants from MSD, Stallergenes, ALK, Allerquim; and is on the advisory board of Hollister-Stier. H Nelson declares he has received fees for consulting from Merck, developer of sublingual tables for sale in the United States and Canada. G Passalacqua declares he has received consulting or lecture fees from Lofarma S.p.A., Stallergenes Italia, GSK, MSD, Novartis, though unrelated to this document. N Rosário declares he has no completing interests. L Rosenwasser declares he has no competing interests. O Pfaar has received research grants from ALK-Abello, Denmark; Allergopharma, Germany; Stallergenes, France; HAL, The Netherlands; Artu Biologicals, The Netherlands; Allergy-Therapeutics/Bencard, UK/Germany; Hartington, Spain; Lofarma, Italy; Novartis/Leti, Germany/Spain; Glaxo-Smith-Kline, UK/Germany; Essex-Pharma, Germany; Cytos, Switzerland; Curalogic, Denmark; Roxall, Germany; Biomay, Austria; Thermo-Fisher, Germany; MEDA-Pharma GmbH, Germany; and/or he has served as advisor and on the speaker’ bureaus for some of the aforementioned pharmaceuticals. He has received travel grants from HAL Allergy, The Netherlands/Germany, and Allergopharma, Germany; and he is a consultant of HAL-Allergy, The Netherlands, LETI-Pharma/Novartis, Germany; Bencard, Germany; Stallergenes, France; and MEDA-Pharma, Germany. D Ryan declares he has received fees from Novartis, Meda, Uriach, GSK, MSD, and AstraZeneca. P Schmid-Grendelmeier declares he has served as consultant for Phadia Thermo Fisher AG, Allergopharma Germany, ALK-Abello Denmark, and Stallergenes France. G Senna, E Valovirta, H Van Bever, P Vichyanond, U Wahn, and O Yusuf, declare that they have no competing interests.

## Authors’ contributions

GWC, LC, and RP are Chairpersons and led the document development. GWC provided new figures for Chapter 1. SRD, LR, and RP contributed Chapter 3. GP, DL-L, MC, and JB contributed Chapter 4. LC, GP, and RGvW contributed Chapter 5. SRD, GWC, EV, and UW contributed Chapter 6. DL-L, CEBC, GVB, and MB contributed Chapter 7. GWC, PS-G, NR, and CEBC contributed Chapter 8. YO and DR contributed Chapter 9. MC, EC, MB and SB contributed Chapter 10. DL-L, MC, PV, RGvW and HN contributed Chapter 11. OP, GS, and EV contributed Chapter 12. MC, RP, GWC, LC, JB, and UW contributed Chapter 13. RP, CEBC, and GWC contributed Chapter 14. Representatives from the Member Societies of the World Allergy Organization reviewed and commented on the document in development. The World Allergy Organization Board of Directors approved the document. All authors reviewed and approved the final document.

## References

[B1] Radulovic S Wilson D Calderon M Durham S Systematic reviews of sublingual immunotherapy (SLIT) Allergy 2011 7 740 752 10.1111/j.1398-9995.2011.02583.x 21443635

[B2] Calderon MA Penagos M Sheikh A Canonica GW Durham SR Sublingual immunotherapy for allergic conjunctivitis: cochrane systematic review and meta-analysis Clin Exp Allergy 2011 7 1263 1272 10.1111/j.1365-2222.2011.03835.x 21848759

[B3] Didier A Worm M Horak F Sussman G de Beaumont O Le Gall M Melac M Malling HJ Sustained 3-year efficacy of pre- and coseasonal 5-grass-pollen sublingual immunotherapy tablets in patients with grass pollen-induced rhinoconjunctivitis J Allergy Clin Immunol 2011 7 559 566 10.1016/j.jaci.2011.06.022 21802126

[B4] Durham SR Emminger W Kapp A de Monchy JG Rak S Scadding GK Wurtzen PA Andersen JS Tholstrup B Riis B Dahl R SQ-standardized sublingual grass immunotherapy: confirmation of disease modification 2 years after 3 years of treatment in a randomized trial J Allergy Clin Immunol 2012 7 717 725 e715 10.1016/j.jaci.2011.12.973 22285278

[B5] Akdis CA Akdis M Mechanisms of allergen-specific immunotherapy J Allergy Clin Immunol 2011 7 18 27 quiz 28-19 10.1016/j.jaci.2010.11.030 21211639

[B6] Maggi E Vultaggio A Matucci A T-cell responses during allergen-specific immunotherapy Curr Opin Allergy Clin Immunol 2012 7 1 6 10.1097/ACI.0b013e32834ecc9a 22157159

[B7] Scadding G Durham S Mechanisms of sublingual immunotherapy J Asthma 2009 7 322 334 10.1080/02770900902785729 19484663

[B8] Scadding G Durham SR Mechanisms of sublingual immunotherapy Immunol Allergy Clin North Am 2011 7 191 209 viii 10.1016/j.iac.2011.02.005 21530814

[B9] Canonica GW Bousquet J Casale T Lockey RF Baena-Cagnani CE Pawankar R Potter PC Bousquet PJ Cox LS Durham SR Nelson HS Passalacqua G Ryan DP Brozek JL Compalati E Dahl R Delgado L van Wijk RG Gower RG Ledford DK Filho NR Valovirta EJ Yusuf OM Zuberbier T Akhanda W Almarales RC Ansotegui I Bonifazi F Ceuppens J Chivato T Sub-lingual immunotherapy: World Allergy Organization position paper 2009 Allergy 2009 7 Suppl 91 1 59 20041860 10.1111/j.1398-9995.2009.02309.x

[B10] Allam JP Duan Y Winter J Stojanovski G Fronhoffs F Wenghoefer M Bieber T Peng WM Novak N Tolerogenic T cells, Th1/Th17 cytokines and TLR2/TLR4 expressing dendritic cells predominate the microenvironment within distinct oral mucosal sites Allergy 2011 7 532 539 10.1111/j.1398-9995.2010.02510.x 21087216

[B11] Allam JP Wurtzen PA Reinartz M Winter J Vrtala S Chen KW Valenta R Wenghoefer M Appel T Gros E Niederhagen B Bieber T Lund K Novak N Phl p 5 resorption in human oral mucosa leads to dose-dependent and time-dependent allergen binding by oral mucosal Langerhans cells, attenuates their maturation, and enhances their migratory and TGF-beta1 and IL-10-producing properties J Allergy Clin Immunol 2010 7 638 645 e631 10.1016/j.jaci.2010.04.039 20584546

[B12] Allam JP Stojanovski G Friedrichs N Peng W Bieber T Wenzel J Novak N Distribution of Langerhans cells and mast cells within the human oral mucosa: new application sites of allergens in sublingual immunotherapy? Allergy 2008 7 720 727 10.1111/j.1398-9995.2007.01611.x 18445186

[B13] Palomares O Ruckert B Jartti T Kucuksezer UC Puhakka T Gomez E Fahrner HB Speiser A Jung A Kwok WW Kalogjera L Akdis M Akdis CA Induction and maintenance of allergen-specific FOXP3+ Treg cells in human tonsils as potential first-line organs of oral tolerance J Allergy Clin Immunol 2012 7 510 520 520 e511-519 10.1016/j.jaci.2011.09.031 22051696

[B14] Kücüksezer UC Palomares O Rückert B Jartti T Puhakka T Nandy A Gemicioğlu B Fahrner HB Jung A Deniz G Akdis CA Akdis M Triggering of specific Toll-like receptors and proinflammatory cytokines breaks allergen-specific T-cell tolerance in human tonsils and peripheral blood J Allergy Clin Immunol 2013 7 875 885 10.1016/j.jaci.2012.10.051 23265862

[B15] Scadding GW Shamji MH Jacobson MR Lee DI Wilson D Lima MT Pitkin L Pilette C Nouri-Aria K Durham SR Sublingual grass pollen immunotherapy is associated with increases in sublingual Foxp3-expressing cells and elevated allergen-specific immunoglobulin G4, immunoglobulin A and serum inhibitory activity for immunoglobulin E-facilitated allergen binding to B cells Clin Exp Allergy 2010 7 598 606 20184605 10.1111/j.1365-2222.2010.03462.x

[B16] Pfaar O Barth C Jaschke C Hormann K Klimek L Sublingual allergen-specific immunotherapy adjuvanted with monophosphoryl lipid A: a phase I/IIa study Int Arch Allergy Immunol 2011 7 336 344 10.1159/000321826 20975285

[B17] Piconi S Trabattoni D Rainone V Borgonovo L Passerini S Rizzardini G Frati F Iemoli E Clerici M Immunological effects of sublingual immunotherapy: clinical efficacy is associated with modulation of programmed cell death ligand 1, IL-10, and IgG4 J Immunol 2010 7 7723 7730 10.4049/jimmunol.1002465 21076061

[B18] O’Hehir RE Gardner LM de Leon MP Hales BJ Biondo M Douglass JA Rolland JM Sandrini A House dust mite sublingual immunotherapy: the role for transforming growth factor-beta and functional regulatory T cells Am J Respir Crit Care Med 2009 7 936 947 10.1164/rccm.200905-0686OC 19696440

[B19] Scadding GW Calderon MA Bellido V Koed GK Nielsen NC Lund K Togias A Phippard D Turka LA Hansel TT Durham SR Wurtzen PA Optimisation of grass pollen nasal allergen challenge for assessment of clinical and immunological outcomes J Immunol Methods 2012 7 25 32 10.1016/j.jim.2012.06.013 22759401

[B20] Swamy RS Reshamwala N Hunter T Vissamsetti S Santos CB Baroody FM Hwang PH Hoyte EG Garcia MA Nadeau KC Epigenetic modifications and improved regulatory T-cell function in subjects undergoing dual sublingual immunotherapy J Allergy Clin Immunol 2012 7 215 224 e217 10.1016/j.jaci.2012.04.021 22677046 PMC4161455

[B21] Van Overtvelt L Baron-Bodo V Horiot S Moussu H Ricarte C Horak F Zieglmayer P Zieglmayer R Montagut A Galvain S de Beaumont O Le Gall M Moingeon P Changes in basophil activation during grass-pollen sublingual immunotherapy do not correlate with clinical efficacy Allergy 2011 7 1530 1537 10.1111/j.1398-9995.2011.02696.x 21883279

[B22] Bohle B Kinaciyan T Gerstmayr M Radakovics A Jahn-Schmid B Ebner C Sublingual immunotherapy induces IL-10-producing T regulatory cells, allergen-specific T-cell tolerance, and immune deviation J Allergy Clin Immunol 2007 7 707 713 10.1016/j.jaci.2007.06.013 17681368

[B23] Fujimura T Yonekura S Taniguchi Y Horiguchi S Saito A Yasueda H Nakayama T Takemori T Taniguchi M Sakaguchi M Okamoto Y The induced regulatory T cell level, defined as the proportion of IL-10(+)Foxp3(+) cells among CD25(+)CD4(+) leukocytes, is a potential therapeutic biomarker for sublingual immunotherapy: a preliminary report Int Arch Allergy Immunol 2010 7 378 387 10.1159/000316349 20559004

[B24] Keles S Karakoc-Aydiner E Ozen A Izgi AG Tevetoglu A Akkoc T Bahceciler NN Barlan I A novel approach in allergen-specific immunotherapy: combination of sublingual and subcutaneous routes J Allergy Clin Immunol 2011 7 808 815 e807 10.1016/j.jaci.2011.04.033 21641635

[B25] Nieminen K Valovirta E Savolainen J Clinical outcome and IL-17, IL-23, IL-27 and FOXP3 expression in peripheral blood mononuclear cells of pollen-allergic children during sublingual immunotherapy Pediatr Allergy Immunol 2010 7 e174 e184 10.1111/j.1399-3038.2009.00920.x 19566585

[B26] Wei W Liu Y Wang Y Zhao Y He J Li X Shen K Induction of CD4 + CD25 + Foxp3 + IL-10+ T cells in HDM-allergic asthmatic children with or without SIT Int Arch Allergy Immunol 2010 7 19 26 10.1159/000301575 20357481

[B27] Eifan AO Akkoc T Yildiz A Keles S Ozdemir C Bahceciler NN Barlan IB Clinical efficacy and immunological mechanisms of sublingual and subcutaneous immunotherapy in asthmatic/rhinitis children sensitized to house dust mite: an open randomized controlled trial Clin Exp Allergy 2010 7 922 932 10.1111/j.1365-2222.2009.03448.x 20100188

[B28] Bonvalet M Moussu H Wambre E Ricarte C Horiot S Rimaniol AC Kwok WW Horak F de Beaumont O Baron-Bodo V Moingeon P Allergen-specific CD4+ T cell responses in peripheral blood do not predict the early onset of clinical efficacy during grass pollen sublingual immunotherapy (ClinicalTrials.gov NCT00619827) Clin Exp Allergy 2012 7 1745 1755 10.1111/cea.12015 23181790

[B29] Bonvalet M Wambre E Moussu H Horiot S Kwok WW Louise A Ebo D Hoarau C Van Overtvelt L Baron-Bodo V Moingeon P Comparison between major histocompatibility complex class II tetramer staining and surface expression of activation markers for the detection of allergen-specific CD4(+) T cells Clin Exp Allergy 2011 7 821 829 10.1111/j.1365-2222.2011.03708.x 21418343

[B30] Campbell JD Buchmann P Kesting S Cunningham CR Coffman RL Hessel EM Allergen-specific T cell responses to immunotherapy monitored by CD154 and intracellular cytokine expression Clin Exp Allergy 2010 7 1025 1035 10.1111/j.1365-2222.2010.03505.x 20412135

[B31] Zimmer A Bouley J Le Mignon M Pliquet E Horiot S Turfkruyer M Baron-Bodo V Horak F Nony E Louise A Moussu H Mascarell L Moingeon P A regulatory dendritic cell signature correlates with the clinical efficacy of allergen-specific sublingual immunotherapy J Allergy Clin Immunol 2012 7 1020 1030 10.1016/j.jaci.2012.02.014 22464673

[B32] Di Lorenzo G Mansueto P Pacor ML Rizzo M Castello F Martinelli N Ditta V Lo Bianco C Leto-Barone MS D’Alcamo A Di Fede G Rini GB Ditto AM Evaluation of serum s-IgE/total IgE ratio in predicting clinical response to allergen-specific immunotherapy J Allergy Clin Immunol 2009 7 1103 1110 1110 e1101-1104 10.1016/j.jaci.2009.02.012 19356792

[B33] Gehlhar K Schlaak M Becker W Bufe A Monitoring allergen immunotherapy of pollen-allergic patients: the ratio of allergen-specific IgG4 to IgG1 correlates with clinical outcome Clin Exp Allergy 1999 7 497 506 10.1046/j.1365-2222.1999.00525.x 10202364

[B34] Asero R Component-resolved diagnosis-assisted prescription of allergen-specific immunotherapy: a practical guide Eur Ann Allergy Clin Immunol 2012 7 183 187 23156065

[B35] Wolthers OD Component-resolved diagnosis in pediatrics ISRN Pediatr 2012 7 806920 22919510 10.5402/2012/806920 PMC3420125

[B36] Würtzen PA Lund G Lund K Arvidsson M Rak S Ipsen H A double-blind placebo-controlled birch allergy vaccination study II: correlation between inhibition of IgE binding, histamine release and facilitated allergen presentation Clin Exp Allergy 2008 7 1290 1301 10.1111/j.1365-2222.2008.03020.x 18510696

[B37] Shamji MH Wilcock LK Wachholz PA Dearman RJ Kimber I Wurtzen PA Larche M Durham SR Francis JN The IgE-facilitated allergen binding (FAB) assay: validation of a novel flow-cytometric based method for the detection of inhibitory antibody responses J Immunol Methods 2006 7 71 79 10.1016/j.jim.2006.09.004 17070537 PMC1934503

[B38] Shamji MH Ljorring C Francis JN Calderon MA Larche M Kimber I Frew AJ Ipsen H Lund K Wurtzen PA Durham SR Functional rather than immunoreactive levels of IgG4 correlate closely with clinical response to grass pollen immunotherapy Allergy 2012 7 217 226 10.1111/j.1398-9995.2011.02745.x 22077562

[B39] Pastorello EA Incorvaia C Ortolani C Bonini S Canonica GW Romagnani S Tursi A Zanussi C Studies on the relationship between the level of specific IgE antibodies and the clinical expression of allergy: I. Definition of levels distinguishing patients with symptomatic from patients with asymptomatic allergy to common aeroallergens J Allergy Clin Immunol 1995 7 580 587 10.1016/S0091-6749(95)70255-5 7499673

[B40] Blaiss M Maloney J Nolte H Gawchik S Yao R Skoner DP Efficacy and safety of timothy grass allergy immunotherapy tablets in North American children and adolescents J Allergy Clin Immunol 2011 7 64 71 71 e61-64 10.1016/j.jaci.2010.11.034 21211642

[B41] Bush RK Swenson C Fahlberg B Evans MD Esch R Morris M Busse WW House dust mite sublingual immunotherapy: results of a US trial J Allergy Clin Immunol 2011 7 974 981 e971-977 10.1016/j.jaci.2010.11.045 21333346

[B42] Cortellini G Spadolini I Patella V Fabbri E Santucci A Severino M Corvetta A Canonica GW Passalacqua G Sublingual immunotherapy for Alternaria-induced allergic rhinitis: a randomized placebo-controlled trial Ann Allergy Asthma Immunol 2010 7 382 386 10.1016/j.anai.2010.08.007 21055665

[B43] Horak F Zieglmayer P Zieglmayer R Lemell P Devillier P Montagut A Melac M Galvain S Jean-Alphonse S Van Overtvelt L Moingeon P Le Gall M Early onset of action of a 5-grass-pollen 300-IR sublingual immunotherapy tablet evaluated in an allergen challenge chamber J Allergy Clin Immunol 2009 7 471 477 477 e471 10.1016/j.jaci.2009.06.006 19647862

[B44] Nelson HS Nolte H Creticos P Maloney J Wu J Bernstein DI Efficacy and safety of timothy grass allergy immunotherapy tablet treatment in North American adults J Allergy Clin Immunol 2011 7 72 80 80 e71-72 10.1016/j.jaci.2010.11.035 21211643

[B45] Panizo C Cimarra M Gonzalez-Mancebo E Vega A Senent C Martin S In vivo and in vitro immunological changes induced by a short course of grass allergy immunotherapy tablets J Investig Allergol Clin Immunol 2010 7 454 462 21243928

[B46] Skoner D Gentile D Bush R Fasano MB McLaughlin A Esch RE Sublingual immunotherapy in patients with allergic rhinoconjunctivitis caused by ragweed pollen J Allergy Clin Immunol 2010 7 660 666 666 e661-666 e664 10.1016/j.jaci.2009.12.931 20153030

[B47] Stelmach I Kaluzinska-Parzyszek I Jerzynska J Stelmach P Stelmach W Majak P Comparative effect of pre-coseasonal and continuous grass sublingual immunotherapy in children Allergy 2012 7 312 320 10.1111/j.1398-9995.2011.02758.x 22142341

[B48] Yonekura S Okamoto Y Sakurai D Horiguchi S Hanazawa T Nakano A Kudou F Nakamaru Y Honda K Hoshioka A Shimojo N Kohno Y Sublingual immunotherapy with house dust extract for house dust-mite allergic rhinitis in children Allergol Int 2010 7 381 388 10.2332/allergolint.10-OA-0200 20864799

[B49] Ahmadiafshar A Maarefvand M Taymourzade B Mazloomzadeh S Torabi Z Efficacy of sublingual swallow immunotherapy in children with rye grass pollen allergic rhinitis: a double-blind placebo-controlled study Iran J Allergy Asthma Immunol 2012 7 175 181 22761191

[B50] Bozek A Ignasiak B Filipowska B Jarzab J House dust mite sublingual immunotherapy: a double-blind, placebo-controlled study in elderly patients with allergic rhinitis Clin Exp Allergy 2013 7 242 248 10.1111/cea.12039 23331565

[B51] Cox LS Casale TB Nayak AS Bernstein DI Creticos PS Ambroisine L Melac M Zeldin RK Clinical efficacy of 300IR 5-grass pollen sublingual tablet in a US study: the importance of allergen-specific serum IgE J Allergy Clin Immunol 2012 7 1327 1334 e1321 10.1016/j.jaci.2012.08.032 23122534

[B52] Creticos PS Maloney J Bernstein DI Casale T Kaur A Fisher R Liu N Murphy K Nekam K Nolte H Randomized controlled trial of a ragweed allergy immunotherapy tablet in North American and European adults J Allergy Clin Immunol 2013 7 1342 1349 e1346 10.1016/j.jaci.2013.03.019 23622121

[B53] de Bot CM Moed H Berger MY Roder E Hop WC de Groot H de Jongste JC van Wijk RG Bindels PJ van der Wouden JC Sublingual immunotherapy not effective in house dust mite-allergic children in primary care Pediatr Allergy Immunol 2012 7 150 158 22017365 10.1111/j.1399-3038.2011.01219.x

[B54] Wahn U Klimek L Ploszczuk A Adelt T Sandner B Trebas-Pietras E Eberle P Bufe A High-dose sublingual immunotherapy with single-dose aqueous grass pollen extract in children is effective and safe: a double-blind, placebo-controlled study J Allergy Clin Immunol 2012 7 886 893 e885 10.1016/j.jaci.2012.06.047 22939758

[B55] Wang DH Chen L Cheng L Li KN Yuan H Lu JH Li H Fast onset of action of sublingual immunotherapy in house dust mite-induced allergic rhinitis: a multicenter, randomized, double-blind, placebo-controlled trial Laryngoscope 2013 7 1334 1340 10.1002/lary.23935 23616386

[B56] Calderon MA Larenas D Kleine-Tebbe J Jacobsen L Passalacqua G Eng PA Varga EM Valovirta E Moreno C Malling HJ Alvarez-Cuesta E Durham S Demoly P European academy of allergy and clinical immunology task force report on ‘dose-response relationship in allergen-specific immunotherapy’ Allergy 2011 7 1345 1359 10.1111/j.1398-9995.2011.02669.x 21707645

[B57] Di Bona D Plaia A Scafidi V Leto-Barone MS Di Lorenzo G Efficacy of sublingual immunotherapy with grass allergens for seasonal allergic rhinitis: a systematic review and meta-analysis J Allergy Clin Immunol 2010 7 558 566 10.1016/j.jaci.2010.06.013 20674964

[B58] Radulovic S Calderon MA Wilson D Durham S Sublingual immunotherapy for allergic rhinitis Cochrane Database Syst Rev 2010 7 12 CD002893 21154351 10.1002/14651858.CD002893.pub2 PMC7001038

[B59] Compalati E Passalacqua G Bonini M Canonica GW The efficacy of sublingual immunotherapy for house dust mites respiratory allergy: results of a GA2LEN meta-analysis Allergy 2009 7 1570 1579 10.1111/j.1398-9995.2009.02129.x 19796205

[B60] Calderon MA Boyle RJ Penagos M Sheikh A Immunotherapy: the meta-analyses. What have we Learned? Immunol Allergy Clin North Am 2011 7 159 173 vii 10.1016/j.iac.2011.02.002 21530812

[B61] Viswanathan RK Busse WW Allergen immunotherapy in allergic respiratory diseases: from mechanisms to meta-analyses Chest 2012 7 1303 1314 10.1378/chest.11-2800 22553263 PMC4694090

[B62] Yukselen A Kendirli SG Yilmaz M Altintas DU Karakoc GB Effect of one-year subcutaneous and sublingual immunotherapy on clinical and laboratory parameters in children with rhinitis and asthma: a randomized, placebo-controlled, double-blind, double-dummy study Int Arch Allergy Immunol 2012 7 288 298 10.1159/000327566 22041501

[B63] Pajno GB Caminiti L Crisafulli G Vita D Valenzise M De Luca R Passalacqua G Direct comparison between continuous and coseasonal regimen for sublingual immunotherapy in children with grass allergy: a randomized controlled study Pediatr Allergy Immunol 2011 7 803 807 10.1111/j.1399-3038.2011.01196.x 21929600

[B64] Pozzan M Milani M Efficacy of sublingual specific immunotherapy in patients with respiratory allergy to Alternaria alternata: a randomised, assessor-blinded, patient-reported outcome, controlled 3-year trial Curr Med Res Opin 2010 7 2801 2806 10.1185/03007995.2010.532201 21050060

[B65] Quercia O Bruno ME Compalati E Falagiani P Mistrello G Stefanini GF Efficacy and safety of sublingual immunotherapy with grass monomeric allergoid: comparison between two different treatment regimens Eur Ann Allergy Clin Immunol 2011 7 176 183 22360134

[B66] Lee JE Choi YS Kim MS Han DH Rhee CS Lee CH Kim DY Efficacy of sublingual immunotherapy with house dust mite extract in polyallergen sensitized patients with allergic rhinitis Ann Allergy Asthma Immunol 2011 7 79 84 10.1016/j.anai.2011.03.012 21704889

[B67] Ciprandi G Cadario G Valle C Ridolo E Verini M Di Gioacchino M Minelli M Gangemi S Sillano V Colangelo C Pravettoni V Pellegrino R Borrelli P Fiorina A Carosso A Gasparini A Riario-Sforza GG Incorvaia C Puccinelli P Scurati S Frati F Sublingual immunotherapy in polysensitized patients: effect on quality of life J Investig Allergol Clin Immunol 2010 7 274 279 20815304

[B68] Calderon MA Cox L Casale TB Moingeon P Demoly P Multiple-allergen and single-allergen immunotherapy strategies in polysensitized patients: looking at the published evidence J Allergy Clin Immunol 2012 7 929 934 10.1016/j.jaci.2011.11.019 22244595

[B69] Cox LS Larenas Linnemann D Nolte H Weldon D Finegold I Nelson HS Sublingual immunotherapy: a comprehensive review J Allergy Clin Immunol 2006 7 1021 1035 10.1016/j.jaci.2006.02.040 16675328

[B70] Bernstein DI Wanner M Borish L Liss GM Twelve-year survey of fatal reactions to allergen injections and skin testing: 1990-2001 J Allergy Clin Immunol 2004 7 1129 1136 10.1016/j.jaci.2004.02.006 15208595

[B71] Epstein TG Liss GM Murphy-Berendts K Bernstein DI Immediate and delayed-onset systemic reactions after subcutaneous immunotherapy injections: ACAAI/AAAAI surveillance study of subcutaneous immunotherapy: year 2 Ann Allergy Asthma Immunol 2011 7 426 431 e421 10.1016/j.anai.2011.05.020 22018614 PMC8207523

[B72] Wessel F Chartier A Meunier JP Magnan A Safety and tolerability of an SQ-standardized GRAss ALlergy immunotherapy tablet (GRAZAX(R)) in a real-life setting for three consecutive seasons - the GRAAL trial Clin Drug Investig 2012 7 451 463 10.2165/11634270-000000000-00000 22594491

[B73] Cox L Larenas-Linnemann D Lockey RF Passalacqua G Speaking the same language: The World Allergy Organization subcutaneous immunotherapy systemic reaction grading system J Allergy Clin Immunol 2010 7 569 574 574 e561-574 e567 10.1016/j.jaci.2009.10.060 20144472

[B74] Pajno GB Caminiti L Crisafulli G Barberi S Landi M Aversa T Valenzise M Passalacqua G Adherence to sublingual immunotherapy in preschool children Pediatr Allergy Immunol 2012 7 688 689 10.1111/j.1399-3038.2012.01317.x 22985448

[B75] Pajno GB Vita D Caminiti L Arrigo T Lombardo F Incorvaia C Barberio G Children’s compliance with allergen immunotherapy according to administration routes J Allergy Clin Immunol 2005 7 1380 1381 10.1016/j.jaci.2005.07.034 16337474

[B76] Passalacqua G Baena-Cagnani CE Bousquet J Canonica GW Casale TB Cox L Durham SR Larenas-Linnemann D Ledford D Pawankar R Potter P Rosario N Wallace D Lockey RF Grading local side effects of sublingual immunotherapy for respiratory allergy: speaking the same language J Allergy Clin Immunol 2013 7 1 93 98 10.1016/j.jaci.2013.03.039 23683513

[B77] Bousquet C Lagier G Lillo-Le Louet A Le Beller C Venot A Jaulent MC Appraisal of the MedDRA conceptual structure for describing and grouping adverse drug reactions Drug Saf 2005 7 19 34 10.2165/00002018-200528010-00002 15649103

[B78] MedDRA Medical Dictionary for Regulatory Activities Maintenance Support Services and Organization Available from: http://www.meddra.org/about-meddra/organisation/msso

[B79] de Groot H Bijl A Anaphylactic reaction after the first dose of sublingual immunotherapy with grass pollen tablet Allergy 2009 7 963 964 10.1111/j.1398-9995.2009.01998.x 19222420

[B80] Blazowski L Anaphylactic shock because of sublingual immunotherapy overdose during third year of maintenance dose Allergy 2008 7 374 18076729 10.1111/j.1398-9995.2007.01563.x

[B81] Eifan AO Keles S Bahceciler NN Barlan IB Anaphylaxis to multiple pollen allergen sublingual immunotherapy Allergy 2007 7 567 568 10.1111/j.1398-9995.2006.01301.x 17313400

[B82] Dunsky EH Goldstein MF Dvorin DJ Belecanech GA Anaphylaxis to sublingual immunotherapy Allergy 2006 7 1235 10.1111/j.1398-9995.2006.01137.x 16942576

[B83] Antico A Pagani M Crema A Anaphylaxis by latex sublingual immunotherapy Allergy 2006 7 1236 1237 10.1111/j.1398-9995.2006.01155.x 16942577

[B84] Calderon MA Simons FE Malling HJ Lockey RF Moingeon P Demoly P Sublingual allergen immunotherapy: mode of action and its relationship with the safety profile Allergy 2012 7 302 311 10.1111/j.1398-9995.2011.02761.x 22150126

[B85] Cochard MM Eigenmann PA Sublingual immunotherapy is not always a safe alternative to subcutaneous immunotherapy J Allergy Clin Immunol 2009 7 378 379 10.1016/j.jaci.2009.04.040 19541352

[B86] Rodriguez-Perez N Ambriz-Moreno Mde J Canonica GW Penagos M Frequency of acute systemic reactions in patients with allergic rhinitis and asthma treated with sublingual immunotherapy Ann Allergy Asthma Immunol 2008 7 304 310 10.1016/S1081-1206(10)60496-6 18814454

[B87] Amin HS Liss GM Bernstein DI Evaluation of near-fatal reactions to allergen immunotherapy injections J Allergy Clin Immunol 2006 7 169 175 10.1016/j.jaci.2005.10.010 16387602

[B88] Tari MG Mancino M Monti G Efficacy of sublingual immunotherapy in patients with rhinitis and asthma due to house dust mite. A double-blind study Allergol Immunopathol (Madr) 1990 7 277 284 2097894

[B89] Niu CK Chen WY Huang JL Lue KH Wang JY Efficacy of sublingual immunotherapy with high-dose mite extracts in asthma: a multi-center, double-blind, randomized, and placebo-controlled study in Taiwan Respir Med 2006 7 1374 1383 10.1016/j.rmed.2005.11.016 16403616

[B90] Kleine-Tebbe J Ribel M Herold DA Safety of a SQ-standardised grass allergen tablet for sublingual immunotherapy: a randomized, placebo-controlled trial Allergy 2006 7 181 184 10.1111/j.1398-9995.2006.00959.x 16409193

[B91] Nayak AS Atiee GJ Dige E Maloney J Nolte H Safety of ragweed sublingual allergy immunotherapy tablets in adults with allergic rhinoconjunctivitis Allergy Asthma Proc 2012 7 404 410 10.2500/aap.2012.33.3605 23026182

[B92] Creticos P Esch RE Couroux P Gentile DA D’Angelo P Whitlow B Alexander M Coyne T A randomized, double-blind, placebo-controlled, parallel trial of standardized short Ragweed (RW) Sublingual Allergy Immunotherapy Liquid Extract (RW-SAIL) in adult subjects with ragweed-induced allergic rhinoconjunctivitis [abstract 519] J Allergy Clin Immunol 2013 7 AB240 AB293 10.1016/j.jaci.2013.10.041 24332263

[B93] Didier A [The development of sublingual desensitization] Rev Prat 2005 7 1334 16180303

[B94] Durham SR Yang WH Pedersen MR Johansen N Rak S Sublingual immunotherapy with once-daily grass allergen tablets: a randomized controlled trial in seasonal allergic rhinoconjunctivitis J Allergy Clin Immunol 2006 7 802 809 10.1016/j.jaci.2005.12.1358 16630937

[B95] Creticos P Maloney J Nolte H Berman G Cheema A Kaur A Hebert J Efficacy and safety of a novel ragweed allergy immunotherapy tablet (AIT) during peak season in North America J Allergy Clin Immunol 2012 7 AB143 10.1016/j.jaci.2011.08.032

[B96] Ott H Sieber J Brehler R Folster-Holst R Kapp A Klimek L Pfaar O Merk H Efficacy of grass pollen sublingual immunotherapy for three consecutive seasons and after cessation of treatment: the ECRIT study Allergy 2009 7 179 186 19076534 10.1111/j.1398-9995.2008.01875.x

[B97] Roger A Justicia JL Navarro LA Eseverri JL Ferres J Malet A Alva V Observational study of the safety of an ultra-rush sublingual immunotherapy regimen to treat rhinitis due to house dust mites Int Arch Allergy Immunol 2011 7 69 75 10.1159/000319211 20664280

[B98] Tripodi S Di Rienzo Businco A Benincori N Scala G Pingitore G Safety and tolerability of ultra-rush induction, less than one hour, of sublingual immunotherapy in children Int Arch Allergy Immunol 2006 7 149 152 10.1159/000090391 16374025

[B99] Sieber J Neis M Brehler R Folster-Holst R Kapp A Klimek L Merk H Increasing long-term safety of seasonal grass pollen sublingual immunotherapy: the ECRIT study Expert Opin Drug Saf 2012 7 7 13 10.1517/14740338.2012.626765 21980934

[B100] Roberts G Hurley C Turcanu V Lack G Grass pollen immunotherapy as an effective therapy for childhood seasonal allergic asthma J Allergy Clin Immunol 2006 7 263 268 10.1016/j.jaci.2005.09.054 16461125

[B101] Rodriguez Perez N Ambriz Moreno Mde J [Safety of immunotherapy and skin tests with allergens in children younger than five years] Rev Alerg Mex 2006 7 47 51 16884027

[B102] Cox L Nelson H Lockey R Calabria C Chacko T Finegold I Nelson M Weber R Bernstein DI Blessing-Moore J Khan DA Lang DM Nicklas RA Oppenheimer J Portnoy JM Randolph C Schuller DE Spector SL Tilles S Wallace D Allergen immunotherapy: a practice parameter third update J Allergy Clin Immunol 2011 7 S1 S55 10.1016/j.jaci.2010.09.034 21122901

[B103] Des Roches A Paradis L Menardo JL Bouges S Daures JP Bousquet J Immunotherapy with a standardized dermatophagoides pteronyssinus extract. VI. Specific immunotherapy prevents the onset of new sensitizations in children J Allergy Clin Immunol 1997 7 450 453 10.1016/S0091-6749(97)70069-1 9111487

[B104] Agostinis F Tellarini L Canonica GW Falagiani P Passalacqua G Safety of sublingual immunotherapy with a monomeric allergoid in very young children Allergy 2005 7 133 10.1111/j.1398-9995.2004.00616.x 15575951

[B105] Fiocchi A Pajno G La Grutta S Pezzuto F Incorvaia C Sensi L Marcucci F Frati F Safety of sublingual-swallow immunotherapy in children aged 3 to 7 years Ann Allergy Asthma Immunol 2005 7 254 258 10.1016/S1081-1206(10)61222-7 16200816

[B106] Rienzo VD Minelli M Musarra A Sambugaro R Pecora S Canonica WG Passalacqua G Post-marketing survey on the safety of sublingual immunotherapy in children below the age of 5 years Clin Exp Allergy 2005 7 560 564 10.1111/j.1365-2222.2005.02219.x 15898975

[B107] Rodriguez Santos O Sublingual immunotherapy in allergic rhinitis and asthma in 2-5 year-old children sensitized to mites [In Spanish] Rev Alerg Mex 2008 7 71 75 19058484

[B108] Agostinis F Foglia C Landi M Cottini M Lombardi C Canonica GW Passalacqua G The safety of sublingual immunotherapy with one or multiple pollen allergens in children Allergy 2008 7 1637 1639 10.1111/j.1398-9995.2008.01742.x 19032238

[B109] Lombardi C Gargioni S Cottini M Canonica GW Passalacqua G The safety of sublingual immunotherapy with one or more allergens in adults Allergy 2008 7 375 376 10.1111/j.1398-9995.2007.01608.x 18269680

[B110] Amar SM Harbeck RJ Sills M Silveira LJ O’Brien H Nelson HS Response to sublingual immunotherapy with grass pollen extract: monotherapy versus combination in a multiallergen extract J Allergy Clin Immunol 2009 7 150 156 e151-155 10.1016/j.jaci.2009.04.037 19523672

[B111] Ciprandi G Cadario G Di Gioacchino M Gangemi S Minelli M Ridolo E Valle C Verini M Boccardo R Incorvaia C Puccinelli P Scurati S Frati F Sublingual immunotherapy in polysensitized allergic patients with rhinitis and/or asthma: allergist choices and treatment efficacy J Biol Regul Homeost Agents 2009 7 165 171 19828093

[B112] Marshall GD Jr AIDS, HIV-positive patients, and allergies Allergy Asthma Proc 1999 7 301 304 10.2500/108854199778251979 10566099

[B113] Randhawa IS Junaid I Klaustermeyer WB Allergen immunotherapy in a patient with human immunodeficiency virus: effect on T-cell activation and viral replication Ann Allergy Asthma Immunol 2007 7 495 497 10.1016/S1081-1206(10)60767-3 17521037

[B114] Steiner UC Furrer H Helbling A Specific immunotherapy in a pollen-allergic patient with human immunodeficiency virus infection World Allergy Organ J 2009 7 57 58 10.1097/WOX.0b013e31819bcae7 23282982 PMC3651045

[B115] Linneberg A Jacobsen RK Jespersen L Abildstrom SZ Association of subcutaneous allergen-specific immunotherapy with incidence of autoimmune disease, ischemic heart disease, and mortality J Allergy Clin Immunol 2012 7 413 419 10.1016/j.jaci.2011.09.007 22004944

[B116] Metzger WJ Turner E Patterson R The safety of immunotherapy during pregnancy J Allergy Clin Immunol 1978 7 268 272 10.1016/0091-6749(78)90202-6 632475

[B117] Shaikh WA A retrospective study on the safety of immunotherapy in pregnancy Clin Exp Allergy 1993 7 857 860 10.1111/j.1365-2222.1993.tb00264.x 10780893

[B118] Shaikh WA Shaikh SW A prospective study on the safety of sublingual immunotherapy in pregnancy Allergy 2012 7 741 743 10.1111/j.1398-9995.2012.02815.x 22486626

[B119] Valovirta E Berstad AK de Blic J Bufe A Eng P Halken S Ojeda P Roberts G Tommerup L Varga EM Winnergard I Design and recruitment for the GAP trial, investigating the preventive effect on asthma development of an SQ-standardized grass allergy immunotherapy tablet in children with grass pollen-induced allergic rhinoconjunctivitis Clin Ther 2011 7 1537 1546 10.1016/j.clinthera.2011.09.013 21999887

[B120] Didier A Malling HJ Worm M Horak F Sussman G Melac M Soulie S Zeldin RK Post-treatment efficacy of discontinuous treatment with 300IR 5-grass pollen sublingual tablet in adults with grass pollen-induced allergic rhinoconjunctivitis Clin Exp Allergy 2013 7 568 577 10.1111/cea.12100 23600548

[B121] Marogna M Spadolini I Massolo A Canonica GW Passalacqua G Long-lasting effects of sublingual immunotherapy according to its duration: a 15-year prospective study J Allergy Clin Immunol 2010 7 969 975 10.1016/j.jaci.2010.08.030 20934206

[B122] Jacobsen L Niggemann B Dreborg S Ferdousi HA Halken S Host A Koivikko A Norberg LA Valovirta E Wahn U Moller C Specific immunotherapy has long-term preventive effect of seasonal and perennial asthma: 10-year follow-up on the PAT study Allergy 2007 7 943 948 10.1111/j.1398-9995.2007.01451.x 17620073

[B123] Canonica GW Bousquet J Casale T Lockey RF Baena-Cagnani CE Pawankar R Potter PC Bousquet PJ Cox LS Durham SR Nelson HS Passalacqua G Ryan DP Brozek JL Compalati E Dahl R Delgado L van Wijk RG Gower RG Ledford DK Filho NR Valovirta EJ Yusuf OM Zuberbier T Sub-lingual immunotherapy: World Allergy Organization position paper 2009 World Allergy Organ J 2009 7 233 281 10.1097/WOX.0b013e3181c6c379 23268425 PMC3488881

[B124] Bufe A Eberle P Franke-Beckmann E Funck J Kimmig M Klimek L Knecht R Stephan V Tholstrup B Weisshaar C Kaiser F Safety and efficacy in children of an SQ-standardized grass allergen tablet for sublingual immunotherapy J Allergy Clin Immunol 2009 7 167 173 e167 10.1016/j.jaci.2008.10.044 19130937

[B125] Wahn U Tabar A Kuna P Halken S Montagut A de Beaumont O Le Gall M Efficacy and safety of 5-grass-pollen sublingual immunotherapy tablets in pediatric allergic rhinoconjunctivitis J Allergy Clin Immunol 2009 7 160 166 e163 10.1016/j.jaci.2008.10.009 19046761

[B126] Brozek JL Akl EA Alonso-Coello P Lang D Jaeschke R Williams JW Phillips B Lelgemann M Lethaby A Bousquet J Guyatt GH Schunemann HJ Grading quality of evidence and strength of recommendations in clinical practice guidelines. Part 1 of 3. An overview of the GRADE approach and grading quality of evidence about interventions Allergy 2009 7 669 677 10.1111/j.1398-9995.2009.01973.x 19210357

[B127] Larenas-Linnemann D Blaiss M Van Bever HP Compalati E Baena-Cagnani CE Pediatric sublingual immunotherapy efficacy: evidence analysis, 2009-2012 Ann Allergy Asthma Immunol 2013 7 402 415 e409 10.1016/j.anai.2013.02.017 23706708

[B128] Valovirta E Jacobsen L Ljorring C Koivikko A Savolainen J Clinical efficacy and safety of sublingual immunotherapy with tree pollen extract in children Allergy 2006 7 1177 1183 10.1111/j.1398-9995.2006.01190.x 16942565

[B129] Halken S Agertoft L Seidenberg J Bauer CP Payot F Martin-Munoz MF Bartkowiak-Emeryk M Vereda A Jean-Alphonse S Melac M Le Gall M Wahn U Five-grass pollen 300IR SLIT tablets: efficacy and safety in children and adolescents Pediatr Allergy Immunol 2010 7 970 976 10.1111/j.1399-3038.2010.01050.x 20718927

[B130] Park D Daher N Blaiss MS Adult and pediatric clinical trials of sublingual immunotherapy in the USA Expert Rev Clin Immunol 2012 7 557 564 10.1586/eci.12.41 22992150

[B131] Stelmach I Kaczmarek-Wozniak J Majak P Olszowiec-Chlebna M Jerzynska J Efficacy and safety of high-doses sublingual immunotherapy in ultra-rush scheme in children allergic to grass pollen Clin Exp Allergy 2009 7 401 408 10.1111/j.1365-2222.2008.03159.x 19134016

[B132] Marogna M Massolo A Colombo F Isella P Bruno M Falagiani P Children passive smoking jeopardises the efficacy of standard anti-allergic pharmacological therapy, while sublingual immunotherapy withstands Allergol Immunopathol (Madr) 2011 7 60 67 10.1016/j.aller.2010.05.002 21216083

[B133] Trebuchon F David M Demoly P Medical management and sublingual immunotherapy practices in patients with house dust mite-induced respiratory allergy: a retrospective, observational study Int J Immunopathol Pharmacol 2012 7 193 206 22507332 10.1177/039463201202500122

[B134] Kim EH Bird JA Kulis M Laubach S Pons L Shreffler W Steele P Kamilaris J Vickery B Burks AW Sublingual immunotherapy for peanut allergy: clinical and immunologic evidence of desensitization J Allergy Clin Immunol 2011 7 640 646 e641 10.1016/j.jaci.2010.12.1083 21281959 PMC3052379

[B135] Keet CA Frischmeyer-Guerrerio PA Thyagarajan A Schroeder JT Hamilton RG Boden S Steele P Driggers S Burks AW Wood RA The safety and efficacy of sublingual and oral immunotherapy for milk allergy J Allergy Clin Immunol 2012 7 448 455 455 e441-445 10.1016/j.jaci.2011.10.023 22130425 PMC3437605

[B136] Grouin JM Vicaut E Jean-Alphonse S Demoly P Wahn U Didier A de Beaumont O Montagut A Le Gall M Devillier P The average adjusted symptom score, a new primary efficacy end-point for specific allergen immunotherapy trials Clin Exp Allergy 2011 7 1282 1288 10.1111/j.1365-2222.2011.03700.x 21375606

[B137] Hrabina M Peltre G Van Ree R Moingeon P Grass pollen allergens Clin Exp Allergy Rev 2008 7 7 11 10.1111/j.1472-9733.2008.00126.x

[B138] Marcucci F Sensi L Incorvaia C Dell’Albani I Di Cara G Frati F Specific IgE response to different grass pollen allergen components in children undergoing sublingual immunotherapy Clin Mol Allergy 2012 7 7 10.1186/1476-7961-10-7 22694773 PMC3511885

[B139] Tripodi S Frediani T Lucarelli S Macri F Pingitore G Di Rienzo Businco A Dondi A Pansa P Ragusa G Asero R Faggian D Plebani M Matricardi PM Molecular profiles of IgE to Phleum pratense in children with grass pollen allergy: implications for specific immunotherapy J Allergy Clin Immunol 2012 7 834 839 e838 10.1016/j.jaci.2011.10.045 22206774

[B140] Ciprandi G Incorvaia C Puccinelli P Dell’Albani I Frati F What should drive the choice of allergen for immunotherapy in polysensitized patients? Ann Allergy Asthma Immunol 2012 7 148 149 10.1016/j.anai.2012.06.007 22840259

[B141] Melioli G Canonica GW Molecular allergy diagnosis: we need to become more knowledgeable Ann Allergy Asthma Immunol 2012 7 387 10.1016/j.anai.2012.04.011 22626588

[B142] Sastre J Landivar ME Ruiz-Garcia M Andregnette-Rosigno MV Mahillo I How molecular diagnosis can change allergen-specific immunotherapy prescription in a complex pollen area Allergy 2012 7 709 711 10.1111/j.1398-9995.2012.02808.x 22379958

[B143] Ninan TK Russell G Respiratory symptoms and atopy in Aberdeen schoolchildren: evidence from two surveys 25 years apart BMJ 1992 7 873 875 10.1136/bmj.304.6831.873 1392746 PMC1882832

[B144] Gupta R Sheikh A Strachan D Anderson HR Increasing hospital admissions for systemic allergic disorders in England: analysis of national admissions data BMJ 2003 7 1142 1143 10.1136/bmj.327.7424.1142 14615340 PMC261813

[B145] Rabe KF Vermeire PA Soriano JB Maier WC Clinical management of asthma in 1999: the Asthma Insights and Reality in Europe (AIRE) study Eur Respir J 2000 7 802 807 10.1183/09031936.00.16580200 11153575

[B146] Ryan D Grant-Casey J Scadding G Pereira S Pinnock H Sheikh A Management of allergic rhinitis in UK primary care: baseline audit Prim Care Respir J 2005 7 204 209 10.1016/j.pcrj.2005.03.009 16701726 PMC6743577

[B147] van Weel C General practitioners’ central role in management of asthma and allergic rhinitis Allergy 2008 7 1005 1007 10.1111/j.1398-9995.2008.01655.x 18691303

[B148] Haubrich W Medical meanings: a glossary of word origins 2003 2 Philadelphia: American College of Physicians

[B149] Kaminski ER Bethune CA Jones RB Complexity of case mix in a regional allergy service BMC Res Notes 2012 7 103 10.1186/1756-0500-5-103 22340023 PMC3305415

[B150] Ryan D van Weel C Bousquet J Toskala E Ahlstedt S Palkonen S van den Nieuwenhof L Zuberbier T Wickman M Fokkens W Primary care: the cornerstone of diagnosis of allergic rhinitis Allergy 2008 7 981 989 10.1111/j.1398-9995.2008.01653.x 18691300

[B151] Baptist AP Baldwin JL Physician attitudes, opinions, and referral patterns: comparisons of those who have and have not taken an allergy/immunology rotation Ann Allergy Asthma Immunol 2004 7 227 231 10.1016/S1081-1206(10)61492-5 15478380

[B152] Shehata Y Ross M Sheikh A Undergraduate allergy teaching in a UK medical school: comparison of the described and delivered curriculum Prim Care Respir J 2007 7 16 21 10.3132/pcrj.2007.00004 17297522 PMC6634171

[B153] Yusuf MO Yusuf SO Khan MS Khan SM Mahmood N ABS26: under-diagnosing allergies and asthma - the need for training primary care physicians Prim Care Respir J 2006 7 192-c

[B154] Potter PC Warner JO Pawankar R Kaliner MA Del Giacco S Rosenwasser L Recommendations for competency in allergy training for undergraduates qualifying as medical practitioners: a position paper of the World Allergy Organization World Allergy Organ J 2009 7 150 154 23283109 10.1186/1939-4551-2-8-150 PMC3651006

[B155] Wallengren J Identification of core competencies for primary care of allergy patients using a modified Delphi technique BMC Med Educ 2011 7 12 10.1186/1472-6920-11-12 21463506 PMC3073956

[B156] Agache I Ryan D Rodriguez MR Yusuf O Angier E Jutel M Allergy management in primary care across European countries – actual status Allergy 2013 7 836 843 10.1111/all.12150 23735183

[B157] Calderón MA Casale T Cox L Akdis CA Burks WA Nelson HS Jutel M Demoly P Allergen immunotherapy: a new semantic framework from the European Academy of Allergy and Clinical Immunology/American Academy of Allergy, Asthma and Immunology/PRACTALL consensus report Allergy 2013 7 825 828 10.1111/all.12180 24010140

[B158] Gøtzsche PC Hammarquist C Burr M House dust mite control measures in the management of asthma: meta-analysis BMJ 1998 7 1105 1110 discussion 1110 10.1136/bmj.317.7166.1105 9784442 PMC28691

[B159] Bousquet J Khaltaev N Cruz AA Denburg J Fokkens WJ Togias A Zuberbier T Baena-Cagnani CE Canonica GW van Weel C Agache I Ait-Khaled N Bachert C Blaiss MS Bonini S Boulet LP Bousquet PJ Camargos P Carlsen KH Chen Y Custovic A Dahl R Demoly P Douagui H Durham SR van Wijk RG Kalayci O Kaliner MA Kim YY Kowalski ML Allergic Rhinitis and its Impact on Asthma (ARIA) 2008 update (in collaboration with the World Health Organization, GA(2)LEN and AllerGen) Allergy 2008 7 Suppl 86 8 160 18331513 10.1111/j.1398-9995.2007.01620.x

[B160] Bateman ED Hurd SS Barnes PJ Bousquet J Drazen JM FitzGerald M Gibson P Ohta K O’Byrne P Pedersen SE Pizzichini E Sullivan SD Wenzel SE Zar HJ Global strategy for asthma management and prevention: GINA executive summary Eur Respir J 2008 7 143 178 10.1183/09031936.00138707 18166595

[B161] Price D Bond C Bouchard J Costa R Keenan J Levy ML Orru M Ryan D Walker S Watson M International Primary Care Respiratory Group (IPCRG) guidelines: management of allergic rhinitis Prim Care Respir J 2006 7 58 70 10.1016/j.pcrj.2005.11.002 16701759 PMC6730679

[B162] Pinnock H Østrem A Rodriguez MR Ryan D Ställberg B Thomas M Tsiligianni I Williams S Yusuf O Prioritising the respiratory research needs of primary care: the International Primary Care Respiratory Group (IPCRG) e-Delphi exercise Prim Care Respir J 2012 7 19 27 10.4104/pcrj.2012.00006 22273628 PMC6547908

[B163] Pinnock H Thomas M Tsiligianni I Lisspers K Østrem A Ställberg B Yusuf O Ryan D Buffels J Cals JW Chavannes NH Henrichsen SH Langhammer A Latysheva E Lionis C Litt J van der Molen T Zwar N Williams S The International Primary Care Respiratory Group (IPCRG) research needs statement 2010 Prim Care Respir J 2010 7 Suppl 1 S1 S20 10.4104/pcrj.2010.00021 PMC6602279 20514388

[B164] Papadopoulos NG Agache I Bavbek S Bilo BM Braido F Cardona V Custovic A Demonchy J Demoly P Eigenmann P Gayraud J Grattan C Heffler E Hellings PW Jutel M Knol E Lotvall J Muraro A Poulsen LK Roberts G Schmid-Grendelmeier P Skevaki C Triggiani M Vanree R Werfel T Flood B Palkonen S Savli R Allegri P Annesi-Maesano I Research needs in allergy: an EAACI position paper, in collaboration with EFA Clin Transl Allergy 2012 7 21 10.1186/2045-7022-2-21 23121771 PMC3539924

[B165] Yusuf OM Management of co-morbid allergic rhinitis and asthma in a low and middle income healthcare setting Prim Care Respir J 2012 7 228 230 10.4104/pcrj.2012.00036 22643360 PMC6547926

[B166] Craig T Sawyer AM Fornadley JA Use of immunotherapy in a primary care office Am Fam Physician 1998 7 1888 1894 1897-1888 9575326

[B167] Kramer J Crocetti S Allergy immunotherapy in the primary care setting J Healthc Qual 2003 7 8 13 quiz 13-14 10.1111/j.1945-1474.2003.tb01082.x 12971098

[B168] Li JT Lockey RF Bernstein IL Portnoy JM Nicklas RA Allergen immunotherapy: a practice parameter Ann Allergy Asthma Immunol 2003 7 1 40

[B169] Platts-Mills T Leung DY Schatz M The role of allergens in asthma Am Fam Physician 2007 7 675 680 17894137

[B170] Bousquet J Heinzerling L Bachert C Papadopoulos NG Bousquet PJ Burney PG Canonica GW Carlsen KH Cox L Haahtela T Lodrup Carlsen KC Price D Samolinski B Simons FE Wickman M Annesi-Maesano I Baena-Cagnani CE Bergmann KC Bindslev-Jensen C Casale TB Chiriac A Cruz AA Dubakiene R Durham SR Fokkens WJ Gerth-van-Wijk R Kalayci O Kowalski ML Mari A Mullol J Practical guide to skin prick tests in allergy to aeroallergens Allergy 2012 7 18 24 10.1111/j.1398-9995.2011.02728.x 22050279

[B171] Niggemann B Nilsson M Friedrichs F Paediatric allergy diagnosis in primary care is improved by in vitro allergen-specific IgE testing Pediatr Allergy Immunol 2008 7 325 331 10.1111/j.1399-3038.2007.00651.x 18312533

[B172] CSM update: desensitising vaccines Br Med J (Clin Res Ed) 1986 7 1 948 20742706 PMC1341725

[B173] Calderon MA Alves B Jacobson M Hurwitz B Sheikh A Durham S Allergen injection immunotherapy for seasonal allergic rhinitis Cochrane Database Syst Rev 2007 1 CD001936 17253469 10.1002/14651858.CD001936.pub2 PMC7017974

[B174] Moreno C Cuesta-Herranz J Fernandez-Tavora L Alvarez-Cuesta E Immunotherapy safety: a prospective multi-centric monitoring study of biologically standardized therapeutic vaccines for allergic diseases Clin Exp Allergy 2004 7 527 531 10.1111/j.1365-2222.2004.1819.x 15080803

[B175] Grazax for hay fever? Drug Ther Bull 2008 7 9 10 18256175 10.1136/dtb.2008.01.0001

[B176] Halken S Lau S Valovirta E New visions in specific immunotherapy in children: an iPAC summary and future trends Pediatr Allergy Immunol 2008 7 Suppl 19 60 70 18665964 10.1111/j.1399-3038.2008.00768.x

[B177] Markert UR Local immunotherapy in allergy: prospects for the future Chem Immunol Allergy 2003 7 127 135 12947999 10.1159/000071547

[B178] Pfaar O Klimek L Efficacy and safety of specific immunotherapy with a high-dose sublingual grass pollen preparation: a double-blind, placebo-controlled trial Ann Allergy Asthma Immunol 2008 7 256 263 10.1016/S1081-1206(10)60451-6 18426146

[B179] Smith H White P Annila I Poole J Andre C Frew A Randomized controlled trial of high-dose sublingual immunotherapy to treat seasonal allergic rhinitis J Allergy Clin Immunol 2004 7 831 837 10.1016/j.jaci.2004.06.058 15480323

[B180] van Wijk RG Sublingual immunotherapy in children Expert Opin Biol Ther 2008 7 291 298 10.1517/14712598.8.3.291 18294100

[B181] Canonica GW Baena-Cagnani CE Compalati E Bohle B Bonini S Bousquet J Cox L Fink-Wagner AH Gonzalez Diaz S Jacobsen L Passalacqua G Pawankar R Vieths S Yusuf O Zuberbier T 100 years of immunotherapy: the Monaco charter. under the high patronage of His Serene Highness Prince Albert II of Monaco Int Arch Allergy Immunol 2013 7 346 349 10.1159/000343883 23183050

[B182] Röder E Berger MY Hop WC Bernsen RM de Groot H Gerth van Wijk R Sublingual immunotherapy with grass pollen is not effective in symptomatic youngsters in primary care J Allergy Clin Immunol 2007 7 892 898 10.1016/j.jaci.2006.12.651 17321581

[B183] Durham SR Sublingual immunotherapy: what have we learnt from the ‘big trials’? Curr Opin Allergy Clin Immunol 2008 7 577 584 10.1097/ACI.0b013e3283196764 18978475

[B184] Hankin CS Cox L Lang D Bronstone A Fass P Leatherman B Wang Z Allergen immunotherapy and health care cost benefits for children with allergic rhinitis: a large-scale, retrospective, matched cohort study Ann Allergy Asthma Immunol 2010 7 79 85 10.1016/j.anai.2009.11.010 20143650

[B185] Calamita Z Saconato H Pela AB Atallah AN Efficacy of sublingual immunotherapy in asthma: systematic review of randomized-clinical trials using the cochrane collaboration method Allergy 2006 7 1162 1172 10.1111/j.1398-9995.2006.01205.x 16942563

[B186] Saporta D McDaniel AB Efficacy comparison of multiple-antigen subcutaneous injection immunotherapy and multiple-antigen sublingual immunotherapy Ear Nose Throat J 2007 7 493 497 17915673

[B187] Esch RE Sublingual immunotherapy Curr Opin Otolaryngol Head Neck Surg 2008 7 260 264 10.1097/MOO.0b013e3282fc706f 18475082

[B188] Esch RE Bush RK Peden D Lockey RF Sublingual-oral administration of standardized allergenic extracts: phase 1 safety and dosing results Ann Allergy Asthma Immunol 2008 7 475 481 10.1016/S1081-1206(10)60474-7 18517081

[B189] Passalacqua G Guerra L Compalati E Canonica GW The safety of allergen specific sublingual immunotherapy Curr Drug Saf 2007 7 117 123 10.2174/157488607780598340 18690957

[B190] Charron M Kramer J Crocetti S Allergy immunotherapy in the primary care setting: integrating national practice standards to promote safe delivery J Nurs Care Qual 2006 7 187 193 10.1097/00001786-200604000-00015 16540788

[B191] de Bot CM Moed H Berger MY Roder E de Groot H de Jongste JC van Wijk RG van der Wouden JC Randomized double-blind placebo-controlled trial of sublingual immunotherapy in children with house dust mite allergy in primary care: study design and recruitment BMC Fam Pract 2008 7 59 10.1186/1471-2296-9-59 18937864 PMC2577674

[B192] Stokes JR Casale TB Allergy immunotherapy for primary care physicians Am J Med 2006 7 820 823 10.1016/j.amjmed.2006.02.019 17000208

[B193] Ciprandi G Marseglia GL Safety of sublingual immunotherapy J Biol Regul Homeost Agents 2011 7 1 6 21382267

[B194] Berto P Frati F Incorvaia C Cadario G Contiguglia R Di Gioacchino M Puccinelli P Senna GE Valle C Comparison of costs of sublingual immunotherapy and drug treatment in grass-pollen induced allergy: results from the SIMAP database study Curr Med Res Opin 2008 7 261 266 10.1185/030079908X253726 18053319

[B195] Brüggenjürgen B Reinhold T Brehler R Laake E Wiese G Machate U Willich SN Cost-effectiveness of specific subcutaneous immunotherapy in patients with allergic rhinitis and allergic asthma Ann Allergy Asthma Immunol 2008 7 316 324 10.1016/S1081-1206(10)60498-X 18814456

[B196] Canonica GW Poulsen PB Vestenbaek U Cost-effectiveness of GRAZAX for prevention of grass pollen induced rhinoconjunctivitis in Southern Europe Respir Med 2007 7 1885 1894 10.1016/j.rmed.2007.05.003 17611095

[B197] Weber RW Allergic rhinitis Prim Care 2008 7 1 10 v 10.1016/j.pop.2007.09.001 18206714

[B198] Mösges R Brüning H Hessler HJ Gotz G Knaussmann HG Sublingual immunotherapy in pollen-induced seasonal rhinitis and conjunctivitis: a randomized controlled trial Acta Dermatovenerol Alp Panonica Adriat 2007 7 143 148 18204744

[B199] Antúnez C Mayorga C Corzo JL Jurado A Torres MJ Two year follow-up of immunological response in mite-allergic children treated with sublingual immunotherapy. Comparison with subcutaneous administration Pediatr Allergy Immunol 2008 7 210 218 10.1111/j.1399-3038.2007.00604.x 18399897

[B200] Cadario G Galluccio AG Pezza M Appino A Milani M Pecora S Mastrandrea F Sublingual immunotherapy efficacy in patients with atopic dermatitis and house dust mites sensitivity: a prospective pilot study Curr Med Res Opin 2007 7 2503 2506 10.1185/030079907X226096 17784996

[B201] Lue KH Lin YH Sun HL Lu KH Hsieh JC Chou MC Clinical and immunologic effects of sublingual immunotherapy in asthmatic children sensitized to mites: a double-blind, randomized, placebo-controlled study Pediatr Allergy Immunol 2006 7 408 415 10.1111/j.1399-3038.2006.00443.x 16925685

[B202] Marogna M Bruno M Massolo A Falagiani P Long-lasting effects of sublingual immunotherapy for house dust mites in allergic rhinitis with bronchial hyperreactivity: a long-term (13-year) retrospective study in real life Int Arch Allergy Immunol 2007 7 70 78 10.1159/000096001 17016060

[B203] Nuhoglu Y Ozumut SS Ozdemir C Ozdemir M Nuhoglu C Erguven M Sublingual immunotherapy to house dust mite in pediatric patients with allergic rhinitis and asthma: a retrospective analysis of clinical course over a 3-year follow-up period J Investig Allergol Clin Immunol 2007 7 375 378 18088019

[B204] Ozdemir C Yazi D Gocmen I Yesil O Aydogan M Semic-Jusufagic A Bahceciler NN Barlan IB Efficacy of long-term sublingual immunotherapy as an adjunct to pharmacotherapy in house dust mite-allergic children with asthma Pediatr Allergy Immunol 2007 7 508 515 10.1111/j.1399-3038.2007.00549.x 17680909

[B205] Pajno GB Caminiti L Vita D Barberio G Salzano G Lombardo F Canonica GW Passalacqua G Sublingual immunotherapy in mite-sensitized children with atopic dermatitis: a randomized, double-blind, placebo-controlled study J Allergy Clin Immunol 2007 7 164 170 10.1016/j.jaci.2007.04.008 17543376

[B206] Frew AJ Smith HE Sublingual immunotherapy J Allergy Clin Immunol 2001 7 441 444 10.1067/mai.2001.113525 11240942

[B207] Marogna M Spadolini I Massolo A Zanon P Berra D Chiodini E Canonica WG Passalacqua G Effects of sublingual immunotherapy for multiple or single allergens in polysensitized patients Ann Allergy Asthma Immunol 2007 7 274 280 10.1016/S1081-1206(10)60718-1 17378260

[B208] Sheikh A Nurmatov U Venderbosch I Bischoff E Oral immunotherapy for the treatment of peanut allergy: systematic review of six case series studies Prim Care Respir J 2012 7 41 49 10.4104/pcrj.2011.00071 21938350 PMC6548306

[B209] Ryan D Levy M Morris A Sheikh A Walker S Management of allergic problems in primary care: time for a rethink? Prim Care Respir J 2005 7 195 203 10.1016/j.pcrj.2005.01.003 16701725 PMC6743575

[B210] Lombardi C Bettoncelli G Canonica GW Passalacqua G The perception of allergen-specific immunotherapy among Italian general practitioners Eur Ann Allergy Clin Immunol 2012 7 80 82 22768727

[B211] Bousquet PJ Calderon MA Demoly P Larenas D Passalacqua G Bachert C Brozek J Canonica GW Casale T Fonseca J Dahl R Durham SR Merk H Worm M Wahn U Zuberbier T Schunemann HJ Bousquet J The Consolidated Standards of Reporting Trials (CONSORT) Statement applied to allergen-specific immunotherapy with inhalant allergens: a Global Allergy and Asthma European Network (GA(2)LEN) article J Allergy Clin Immunol 2011 7 49 56 56 e41-11 10.1016/j.jaci.2010.09.017 21112079

[B212] Bousquet PJ Brozek J Bachert C Bieber T Bonini S Burney P Calderon M Canonica GW Compalati E Daures JP Delgado L Demoly P Dahl R Durham SR Kowalski ML Malling HJ Merk H Papadopoulos N Passalacqua G Simon HU Worms M Wahn U Zuberbier T Schunemann HJ Bousquet J The CONSORT statement checklist in allergen-specific immunotherapy: a GA2LEN paper Allergy 2009 7 1737 1745 10.1111/j.1398-9995.2009.02232.x 19860788

[B213] Sieber J Necessity of product-specific assessments or restrictions of meta-analyses to well-designed and well-powered studies J Allergy Clin Immunol 2011 7 1075 1076 author reply 1077-1078 10.1016/j.jaci.2010.12.1092 21306765

[B214] de Bot CM Moed H Berger MY Roder E van Wijk RG van der Wouden JC Sublingual immunotherapy in children with allergic rhinitis: quality of systematic reviews Pediatr Allergy Immunol 2011 7 548 558 10.1111/j.1399-3038.2011.01165.x 21919934

[B215] Bousquet J Schunemann HJ Bousquet PJ Bachert C Canonica GW Casale TB Demoly P Durham S Carlsen KH Malling HJ Passalacqua G Simons FE Anto J Baena-Cagnani CE Bergmann KC Bieber T Briggs AH Brozek J Calderon MA Dahl R Devillier P Gerth van Wijk R Howarth P Larenas D Papadopoulos NG Schmid-Grendelmeier P Zuberbier T How to design and evaluate randomized controlled trials in immunotherapy for allergic rhinitis: an ARIA-GA(2)LEN statement Allergy 2011 7 765 774 10.1111/j.1398-9995.2011.02590.x 21496059

[B216] Howarth P Malling HJ Molimard M Devillier P Analysis of allergen immunotherapy studies shows increased clinical efficacy in highly symptomatic patients Allergy 2012 7 321 327 10.1111/j.1398-9995.2011.02759.x 22142377

[B217] Kulis M Saba K Kim EH Bird JA Kamilaris N Vickery BP Staats H Burks AW Increased peanut-specific IgA levels in saliva correlate with food challenge outcomes after peanut sublingual immunotherapy J Allergy Clin Immunol 2012 7 1159 1162 10.1016/j.jaci.2011.11.045 22236732 PMC3763925

[B218] Narisety SD Keet CA Sublingual vs oral immunotherapy for food allergy: identifying the right approach Drugs 2012 7 1977 1989 10.2165/11640800-000000000-00000 23009174 PMC3708591

[B219] Cortellini G Spadolini I Santucci A Cova V Conti C Corvetta A Passalacqua G Improvement of shrimp allergy after sublingual immunotherapy for house dust mites: a case report Eur Ann Allergy Clin Immunol 2011 7 162 164 22145252

[B220] Mauro M Russello M Incorvaia C Gazzola G Frati F Moingeon P Passalacqua G Birch-apple syndrome treated with birch pollen immunotherapy Int Arch Allergy Immunol 2011 7 416 422 10.1159/000323909 21832831

[B221] Nucera E Aruanno A Rizzi A Buonomo A Pecora V Colagiovanni A Pascolini L Ricci AG Schiavino D Profilin desensitization in two patients with plant-derived food allergy Int J Immunopathol Pharmacol 2012 7 531 535 22697087 10.1177/039463201202500225

[B222] Compalati E Rogkakou A Passalacqua G Canonica GW Evidences of efficacy of allergen immunotherapy in atopic dermatitis: an updated review Curr Opin Allergy Clin Immunol 2012 7 427 433 10.1097/ACI.0b013e328354e540 22622475

[B223] Lasa Luaces EM Tabar Purroy AI Garcia Figueroa BE Anda Apinaniz M Sanz Laruga ML Raulf-Heimsoth M Barber Hernandez D Component-resolved immunologic modifications, efficacy, and tolerance of latex sublingual immunotherapy in children Ann Allergy Asthma Immunol 2012 7 367 372 10.1016/j.anai.2012.03.005 22541410

[B224] Gastaminza G Algorta J Uriel O Audicana MT Fernandez E Sanz ML Munoz D Randomized, double-blind, placebo-controlled clinical trial of sublingual immunotherapy in natural rubber latex allergic patients Trials 2011 7 191 10.1186/1745-6215-12-191 21827704 PMC3175458

[B225] Rossi R Monasterolo G Passalacqua G The biological potency of different extracts for sublingual immunotherapy assessed by skin prick tests Eur Ann Allergy Clin Immunol 2010 7 112 114 20648773

[B226] van Ree R Chapman MD Ferreira F Vieths S Bryan D Cromwell O Villalba M Durham SR Becker WM Aalbers M Andre C Barber D Cistero Bahima A Custovic A Didierlaurent A Dolman C Dorpema JW Di Felice G Eberhardt F Fernandez Caldas E Fernandez Rivas M Fiebig H Focke M Fotisch K Gadermaier G Das RG Gonzalez Mancebo E Himly M Kinaciyan T Knulst AC The CREATE project: development of certified reference materials for allergenic products and validation of methods for their quantification Allergy 2008 7 310 326 10.1111/j.1398-9995.2007.01612.x 18269676

[B227] Larenas-Linnemann D Esch R Plunkett G Brown S Maddox D Barnes C Constable D Maintenance dosing for sublingual immunotherapy by prominent European allergen manufacturers expressed in bioequivalent allergy units Ann Allergy Asthma Immunol 2011 7 448 458 e443 10.1016/j.anai.2011.07.001 22018618

[B228] Chapman MD Ferreira F Villalba M Cromwell O Bryan D Becker WM Fernandez-Rivas M Durham S Vieths S van Ree R The European Union CREATE project: a model for international standardization of allergy diagnostics and vaccines J Allergy Clin Immunol 2008 7 882 889 e882 10.1016/j.jaci.2008.07.030 18762328

[B229] Kette F Smith W Sublingual immunotherapy: allergen specific or placebo effect? J Allergy Clin Immunol 2011 7 430 author reply 430-432 21665261 10.1016/j.jaci.2011.04.048

[B230] Blaiss M Nelson HS Durham SR Dahl R Bufe A Reply J Allergy Clin Immunol 2011 7 430 43221665261

[B231] Didier A Reply J Allergy Clin Immunol 2011 7 43221665255

[B232] Wilson AM O’Byrne PM Parameswaran K Leukotriene receptor antagonists for allergic rhinitis: a systematic review and meta-analysis Am J Med 2004 7 338 344 10.1016/j.amjmed.2003.10.030 14984820

[B233] Canonica GW Baena-Cagnani CE Bousquet J Bousquet PJ Lockey RF Malling HJ Passalacqua G Potter P Valovirta E Recommendations for standardization of clinical trials with allergen specific immunotherapy for respiratory allergy. A statement of a World Allergy Organization (WAO) taskforce Allergy 2007 7 317 324 10.1111/j.1398-9995.2006.01312.x 17298350

[B234] Bousquet J Anto JM Demoly P Schunemann HJ Togias A Akdis M Auffray C Bachert C Bieber T Bousquet PJ Carlsen KH Casale TB Cruz AA Keil T Lodrup Carlsen KC Maurer M Ohta K Papadopoulos NG Roman Rodriguez M Samolinski B Agache I Andrianarisoa A Ang CS Annesi-Maesano I Ballester F Baena-Cagnani CE Basagana X Bateman ED Bel EH Bedbrook A Severe chronic allergic (and related) diseases: a uniform approach–a MeDALL–GA2LEN–ARIA position paper Int Arch Allergy Immunol 2012 7 216 231 22382913 10.1159/000332924

[B235] Calderon M Cardona V Demoly P One hundred years of allergen immunotherapy European Academy of Allergy and Clinical Immunology celebration: review of unanswered questions Allergy 2012 7 462 476 10.1111/j.1398-9995.2012.02785.x 22309435

[B236] Francis JN The facilitated antigen binding (FAB) assay–a protocol to measure allergen-specific inhibitory antibody activity Methods Mol Med 2008 7 255 261 10.1007/978-1-59745-366-0_21 18612614

[B237] Brito FF Gimeno PM Carnes J Martin R Fernandez-Caldas E Lara P Lopez-Fidalgo J Guerra F Olea europaea pollen counts and aeroallergen levels predict clinical symptoms in patients allergic to olive pollen Ann Allergy Asthma Immunol 2011 7 146 152 10.1016/j.anai.2010.11.003 21277516

[B238] Jariwala SP Kurada S Moday H Thanjan A Bastone L Khananashvili M Fodeman J Hudes G Rosenstreich D Association between tree pollen counts and asthma ED visits in a high-density urban center J Asthma 2011 7 442 448 10.3109/02770903.2011.567427 21453203

[B239] Kirmaz C Yuksel H Bayrak P Yilmaz O Symptoms of the olive pollen allergy: do they really occur only in the pollination season? J Investig Allergol Clin Immunol 2005 7 140 145 16047715

[B240] Buters JT Weichenmeier I Ochs S Pusch G Kreyling W Boere AJ Schober W Behrendt H The allergen Bet v 1 in fractions of ambient air deviates from birch pollen counts Allergy 2010 7 850 858 10.1111/j.1398-9995.2009.02286.x 20132158

[B241] Ellis AK Ratz JD Day AG Day JH Factors that affect the allergic rhinitis response to ragweed allergen exposure Ann Allergy Asthma Immunol 2010 7 293 298 10.1016/j.anai.2010.01.012 20408338

[B242] Feo Brito F Mur Gimeno P Carnes J Fernandez-Caldas E Lara P Alonso AM Garcia R Guerra F Grass pollen, aeroallergens, and clinical symptoms in Ciudad Real, Spain J Investig Allergol Clin Immunol 2010 7 295 302 20815307

[B243] Boehm G Leuschner RM Experiences with the ‘Individual Pollen Collector’ developed by G. Boehm Experientia Suppl 1987 7 87 88 10.1007/978-3-0348-7491-5_15 2958356

[B244] Frew A Fischer Von Weikersthal-Drache K Huber B Lee D An innovative approach to the analysis of seasonal diaries after specific immunotherapy—definition of peak season based on symptom/medication scores of the placebo group rather than on pollen counts [abstract 1876] Allergy 2010 7 69019909295

[B245] Frew AJ DuBuske L Keith PK Corrigan CJ Aberer W von Weikersthal-Drachenberg KJ F Assessment of specific immunotherapy efficacy using a novel placebo score-based method Ann Allergy Asthma Immunol 2012 7 342 347 e341 10.1016/j.anai.2012.08.013 23062390

[B246] Longhi S Cristofori A Gatto P Cristofolini F Grando MS Gottardini E Biomolecular identification of allergenic pollen: a new perspective for aerobiological monitoring? Ann Allergy Asthma Immunol 2009 7 508 514 10.1016/S1081-1206(10)60268-2 20084845

[B247] Commitee for medicinal products for human use (CHMP) Guideline on the clinical development of products for specific immunotherapy for the treatment of allergic diseases. Document No. CHMP/EWP/18504/2006 2008 London Available at: http://www.ema.europa.eu/docs/en_GB/document_library/Scientific_guideline/2009/09/WC500003605.pdf

[B248] Häfner D Reich K Matricardi PM Meyer H Kettner J Narkus A Prospective validation of ‘Allergy-Control-SCORE(TM)’: a novel symptom-medication score for clinical trials Allergy 2011 7 629 636 10.1111/j.1398-9995.2010.02531.x 21261656

[B249] Pfaar O Robinson DS Sager A Emuzyte R Immunotherapy with depigmented-polymerized mixed tree pollen extract: a clinical trial and responder analysis Allergy 2010 7 1614 1621 10.1111/j.1398-9995.2010.02413.x 20645937

[B250] Baiardini I Bousquet PJ Brzoza Z Canonica GW Compalati E Fiocchi A Fokkens W van Wijk RG La Grutta S Lombardi C Maurer M Pinto AM Ridolo E Senna GE Terreehorst I Bom AT Bousquet J Zuberbier T Braido F Recommendations for assessing patient-reported outcomes and health-related quality of life in clinical trials on allergy: a GA(2)LEN taskforce position paper Allergy 2010 7 290 295 10.1111/j.1398-9995.2009.02263.x 19930232

[B251] Braido F Bousquet PJ Brzoza Z Canonica GW Compalati E Fiocchi A Fokkens W Gerth van Wijk R La Grutta S Lombardi C Maurer M Pinto AM Ridolo E Senna GE Terreehorst I Todo Bom A Bousquet J Zuberbier T Baiardini I Specific recommendations for PROs and HRQoL assessment in allergic rhinitis and/or asthma: a GA(2)LEN taskforce position paper Allergy 2010 7 959 968 10.1111/j.1398-9995.2010.02383.x 20486919

[B252] Calderon MA Eichel A Makatsori M Pfaar O Comparability of subcutaneous and sublingual immunotherapy outcomes in allergic rhinitis clinical trials Curr Opin Allergy Clin Immunol 2012 7 249 256 10.1097/ACI.0b013e32835358b3 22499145

[B253] Durham SR Birk AO Andersen JS Days with severe symptoms: an additional efficacy endpoint in immunotherapy trials Allergy 2011 7 120 123 10.1111/j.1398-9995.2010.02434.x 20608918

[B254] Hohlfeld JM Holland-Letz T Larbig M Lavae-Mokhtari M Wierenga E Kapsenberg M van Ree R Krug N Bufe A Diagnostic value of outcome measures following allergen exposure in an environmental challenge chamber compared with natural conditions Clin Exp Allergy 2010 7 998 1006 10.1111/j.1365-2222.2010.03498.x 20412138

[B255] Krug N Usefulness of pollen chamber in the evaluation of clinical trials with allergen products Arb Paul Ehrlich Inst Bundesamt Sera Impfstoffe Frankf A M 2006 7 167 170 discussion 170-161 17393736

[B256] Bernstein JA Correlation between a pollen challenge chamber and a natural allergen exposure study design for eliciting ocular and nasal symptoms: early evidence supporting a paradigm shift in drug investigation? J Allergy Clin Immunol 2012 7 128 129 10.1016/j.jaci.2012.05.032 22742840

[B257] Devillier P Le Gall M Horak F The allergen challenge chamber: a valuable tool for optimizing the clinical development of pollen immunotherapy Allergy 2011 7 163 169 10.1111/j.1398-9995.2010.02473.x 21039599

[B258] Pfaar O Kleine-Tebbe J Hormann K Klimek L Allergen-specific immunotherapy: which outcome measures are useful in monitoring clinical trials? Immunol Allergy Clin North Am 2011 7 289 309 x 10.1016/j.iac.2011.02.004 21530821

[B259] Senna G Calderon M Makatsori M Ridolo E Passalacqua G An evidence-based appraisal of the surrogate markers of efficacy of allergen immunotherapy Curr Opin Allergy Clin Immunol 2011 7 375 380 10.1097/ACI.0b013e328348a7cd 21670667

[B260] Kuna P Samolinski B Worm M Pfaar O Klimek L Sustained clinical efficacy of sublingual immunotherapy with a high-dose grass pollen extract Eur Ann Allergy Clin Immunol 2011 7 117 121 21980799

[B261] Durham SR Emminger W Kapp A Colombo G de Monchy JG Rak S Scadding GK Andersen JS Riis B Dahl R Long-term clinical efficacy in grass pollen-induced rhinoconjunctivitis after treatment with SQ-standardized grass allergy immunotherapy tablet J Allergy Clin Immunol 2010 7 131 138 e131-137 10.1016/j.jaci.2009.10.035 20109743

[B262] Senna G Ridolo E Calderon M Lombardi C Canonica GW Passalacqua G Evidence of adherence to allergen-specific immunotherapy Curr Opin Allergy Clin Immunol 2009 7 544 548 10.1097/ACI.0b013e328332b8df 19823080

[B263] Senna G Lombardi C Canonica GW Passalacqua G How adherent to sublingual immunotherapy prescriptions are patients? The manufacturers’ viewpoint J Allergy Clin Immunol 2010 7 668 669 10.1016/j.jaci.2010.06.045 20816199

[B264] Sieber J De Geest S Shah-Hosseini K Mösges R Medication persistence with long-term, specific grass pollen immunotherapy measured by prescription renewal rates Curr Med Res Opin 2011 7 855 861 10.1185/03007995.2011.559538 21323505

[B265] Passalacqua G Frati F Puccinelli P Scurati S Incorvaia C Canonica GW Hilaire C Adherence to sublingual immunotherapy: the allergists’ viewpoint Allergy 2009 7 1796 1797 10.1111/j.1398-9995.2009.02136.x 19712121

[B266] Scurati S Frati F Passalacqua G Puccinelli P Hilaire C Incorvaia C Adherence issues related to sublingual immunotherapy as perceived by allergists Patient Prefer Adherence 2010 7 141 145 20622914 10.2147/ppa.s10217 PMC2898115

[B267] Hsu NM Reisacher WR A comparison of attrition rates in patients undergoing sublingual immunotherapy vs subcutaneous immunotherapy Int Forum Allergy Rhinol 2012 7 280 284 10.1002/alr.21037 22434716

[B268] Incorvaia C Mauro M Ridolo E Puccinelli P Liuzzo M Scurati S Frati F Patient’s compliance with allergen immunotherapy Patient Prefer Adherence 2008 7 247 251 19920970 10.2147/ppa.s3806 PMC2770419

[B269] Savi E Peveri S Senna G Passalacqua G Causes of SLIT discontinuation and strategies to improve the adherence: a pragmatic approach Allergy 2013 7 9 1193 1195 23915164 10.1111/all.12198

[B270] Vita D Caminiti L Ruggeri P Pajno GB Sublingual immunotherapy: adherence based on timing and monitoring control visits Allergy 2010 7 668 669 10.1111/j.1398-9995.2009.02223.x 19845569

[B271] Calderon MA Gerth van Wijk R Eichler I Matricardi PM Varga EM Kopp MV Eng P Niggemann B Nieto A Valovirta E Eigenmann PA Pajno G Bufe A Halken S Beyer K Wahn U Perspectives on allergen-specific immunotherapy in childhood: an EAACI position statement Pediatr Allergy Immunol 2012 7 300 306 10.1111/j.1399-3038.2012.01313.x 22594930

[B272] Holt PG Sly PD Sampson HA Robinson P Loh R Lowenstein H Calatroni A Sayre P Prophylactic use of sublingual allergen immunotherapy in high-risk children: a pilot study J Allergy Clin Immunol 2013 7 4 991 993.31 10.1016/j.jaci.2013.04.049 23768574

[B273] Simoens S The cost-effectiveness of immunotherapy for respiratory allergy: a review Allergy 2012 7 1087 1105 10.1111/j.1398-9995.2012.02861.x 22765521

[B274] Hagen A Gorenoi V Schonermark MP Specific immunotherapy (SIT) in the treatment of allergic rhinitis GMS Health Technol Assess 2010 7 Doc01 21289874 10.3205/hta000079 PMC3010882

[B275] Meadows A Kaambwa B Novielli N Huissoon A Fry-Smith A Meads C Barton P Dretzke J A systematic review and economic evaluation of subcutaneous and sublingual allergen immunotherapy in adults and children with seasonal allergic rhinitis Health Technol Assess 2013 7 1 322 vi, xi-xiv 10.3310/hta17270 PMC4780904 23827204

[B276] Barton S Which clinical studies provide the best evidence? The best RCT still trumps the best observational study BMJ 2000 7 255 256 10.1136/bmj.321.7256.255 10915111 PMC1118259

[B277] Guyatt GH Oxman AD Vist GE Kunz R Falck-Ytter Y Alonso-Coello P Schunemann HJ GRADE: an emerging consensus on rating quality of evidence and strength of recommendations BMJ 2008 7 924 926 10.1136/bmj.39489.470347.AD 18436948 PMC2335261

[B278] Current status of allergen immunotherapy. Shortened version of a World Health Organisation/International Union of Immunological Societies working group report Lancet 1989 7 259 261 2563421

[B279] Malling H-J Immunotherapy Allergy 1988 7 9 33

[B280] Malling HJ Weeke B Position paper: immunotherapy Allergy 1993 7 9 35 10.1111/j.1398-9995.1993.tb04754.x 8342741

[B281] Bousquet J Lockey R Malling HJ Alvarez-Cuesta E Canonica GW Chapman MD Creticos PJ Dayer JM Durham SR Demoly P Goldstein RJ Ishikawa T Ito K Kraft D Lambert PH Lowenstein H Muller U Norman PS Reisman RE Valenta R Valovirta E Yssel H Allergen immunotherapy: therapeutic vaccines for allergic diseases. World Health Organization. American academy of Allergy, Asthma and Immunology Ann Allergy Asthma Immunol 1998 7 401 405 10.1016/S1081-1206(10)63136-5 9860031

[B282] Malling HJ Abreu-Nogueira J Alvarez-Cuesta E Bjorksten B Bousquet J Caillot D Canonica GW Passalacqua G Saxonis-Papageorgiou P Valovirta E Local immunotherapy Allergy 1998 7 933 944 10.1111/j.1398-9995.1998.tb03793.x 9821472

[B283] Joint Task Force on Practice Parameters Allergen immunotherapy: a practice parameter second update J Allergy Clin Immunol 2007 7 S25 S85 10.1016/j.jaci.2007.06.019 17765078

[B284] Shekelle PG Woolf SH Eccles M Grimshaw J Clinical guidelines: developing guidelines BMJ 1999 7 593 596 10.1136/bmj.318.7183.593 10037645 PMC1115034

[B285] Leith E Bowen T Butchey J Fischer D Kim H Moote B Small P Stark D Waserman S Consensus Guidelines on Practical Issues of Immunotherapy-Canadian Society of Allergy and Clinical Immunology (CSACI) Allergy Asthma Clin Immunol 2006 7 47 61 20525157 10.1186/1710-1492-2-2-47 PMC2876183

[B286] Saranz RJ Lozano A Caceres ME Arnolt RG Maspero JF Bozzola CM Neffen HE Croce VH Gualtier O Procopio N [Allergen immunotherapy for prevention and treatment of respiratory allergy in childhood] Arch Argent Pediatr 2010 7 258 265 20544144 10.1590/S0325-00752010000300020

[B287] Walker SM Durham SR Till SJ Roberts G Corrigan CJ Leech SC Krishna MT Rajakulasingham RK Williams A Chantrell J Dixon L Frew AJ Nasser SM Immunotherapy for allergic rhinitis Clin Exp Allergy 2011 7 1177 1200 10.1111/j.1365-2222.2011.03794.x 21848757

[B288] Larenas-Linnemann D Ortega-Martell JA Del Rio-Navarro B Rodriguez-Perez N Arias-Cruz A Estrada A Becerril-Angeles M Pietropaolo-Cienfuegos DR Ambriz-Moreno Mde J Baez-Loyola C Cossio-Ochoa E Gonzalez-Diaz SN Hidalgo-Castro EM Huerta-Hernandez RE Macias-Weinmann A Oyoqui-Flores J Stone-Aguilar H Trevino-Salinas MB Zarate-Hernandez Mdel C [Mexican clinical practice guidelines of immunotherapy 2011] Rev Alerg Mex 2011 7 3 75 21967873

[B289] Subspecialty Group of Rhinology Expert consensus on allergen specific immunotherapy of allergic rhinitis Zhonghua Er Bi Yan Hou Tou Jing Wai Ke Za Zhi [Chinese Journal of Otorhinolaryngology Head and Neck Surgery] 2011 7 12 976 980 [Article in Chinese] 22336007

[B290] Valovirta E Korhonen K Kuitunen M Kukkonen-Harjula K Lammintausta K Pallasaho P Ralli P Savolainen J Toskala E Virtanen T [Update on current care guidelines: allergen specific immunotherapy] Duodecim; laaketieteellinen aikakauskirja 2012 7 108 109 22312833

[B291] Bousquet J Van Cauwenberge P Khaltaev N Allergic rhinitis and its impact on asthma J Allergy Clin Immunol 2001 7 S147 S334 10.1067/mai.2001.118891 11707753

[B292] Brozek JL Bousquet J Baena-Cagnani CE Bonini S Canonica GW Casale TB van Wijk RG Ohta K Zuberbier T Schunemann HJ Global A, Asthma European N, Grading of Recommendations Assessment D, Evaluation Working G: Allergic Rhinitis and its Impact on Asthma (ARIA) guidelines: 2010 revision J Allergy Clin Immunol 2010 7 466 476 10.1016/j.jaci.2010.06.047 20816182

[B293] Zuberbier T Bachert C Bousquet PJ Passalacqua G Walter Canonica G Merk H Worm M Wahn U Bousquet J GA(2) LEN/EAACI pocket guide for allergen-specific immunotherapy for allergic rhinitis and asthma Allergy 2010 7 1525 1530 10.1111/j.1398-9995.2010.02474.x 21039596

[B294] Krishna MT Ewan PW Diwakar L Durham SR Frew AJ Leech SC Nasser SM British Society for A, Clinical I: Diagnosis and management of hymenoptera venom allergy: British Society for Allergy and Clinical Immunology (BSACI) guidelines Clin Exp Allergy 2011 7 1201 1220 10.1111/j.1365-2222.2011.03788.x 21848758

[B295] Ring J Alomar A Bieber T Deleuran M Fink-Wagner A Gelmetti C Gieler U Lipozencic J Luger T Oranje AP Schafer T Schwennesen T Seidenari S Simon D Stander S Stingl G Szalai S Szepietowski JC Taieb A Werfel T Wollenberg A Darsow U European Dermatology F, European Academy of D, Venereology, European Task Force on Atopic D, European Federation of A, European Society of Pediatric D, Global A, Asthma European N: guidelines for treatment of atopic eczema (atopic dermatitis) Part II Journal of the European Academy of Dermatology and Venereology: JEADV 2012 7 1176 1193 10.1111/j.1468-3083.2012.04636.x 22813359

[B296] Coifman RE Cox LS American Academy of Allergy, Asthma & Immunology member immunotherapy practice patterns and concerns J Allergy Clin Immunol 2006 7 119 1012 1013 10.1016/j.jaci.2007.01.029 17418663

[B297] Sikora JM Tankersley MS Immunotherapy A Diagnostics C Perception and practice of sublingual immunotherapy among practicing allergists in the United States: a follow-up survey Ann Allergy Asthma Immunol 2013 7 194 197 e194 10.1016/j.anai.2012.12.014 23548531

[B298] Tucker MH Tankersley MS Perception and practice of sublingual immunotherapy among practicing allergists Ann Allergy Asthma Immunol 2008 7 419 425 10.1016/S1081-1206(10)60320-1 18939732

[B299] Bernstein DI Blaiss MS Cox LS Finegold I Lanier BQ Nelson HS Wallace DV Current standards and future directions in immunotherapy: perspectives on challenges and opportunities for the allergist Ann Allergy Asthma Immunol 2011 7 422 425 10.1016/j.anai.2011.05.026 22018613

[B300] Antonicelli L Braschi MC Bilo MB Angino A Pala AP Baldacci S Maio S Bonifazi F Congruence between international guidelines and mite specific immunotherapy prescribing practices Respir Med 2011 7 1441 1448 10.1016/j.rmed.2011.05.003 21628094

[B301] Bernstein DI Epstein T Murphy-Berendts K Liss GM Surveillance of systemic reactions to subcutaneous immunotherapy injections: year 1 outcomes of the ACAAI and AAAAI collaborative study Ann Allergy Asthma Immunol 2010 7 530 535 10.1016/j.anai.2010.04.008 20568387 PMC8246419

[B302] Scadding GK Brostoff J Low dose sublingual therapy in patients with allergic rhinitis due to house dust mite Clin Allergy 1986 7 483 491 10.1111/j.1365-2222.1986.tb01983.x 3536171

[B303] Larenas-Linneman D Cox LS European allergen extract units and potency: review of available information Ann Allergy Asthma Immunol 2008 7 2 137 145 10.1016/S1081-1206(10)60422-X 18320915

[B304] Sander I Fleischer C Meurer U Bruning T Raulf-Heimsoth M Allergen content of grass pollen preparations for skin prick testing and sublingual immunotherapy Allergy 2009 7 1486 1492 10.1111/j.1398-9995.2009.02040.x 19385952

[B305] Distler A Lumovic J [Majorallergenbestimmung: publizierte Daten aus sublingualen Allergenpräparaten (AP) bei Gräserpollen (Phelum pratense, Phl p 5) und Birkenpollen (Betula verrucosa, Bet v1) von verschiedenen Herstellern im Vergleich zu Messergebnissen aus externen Laboratorien] Allergo J 2011 7 S41 S42

[B306] van den Hout R Peekel I Busch M Kervliet E Amount of major allergen Phl p 5 and allergen activity in different European products for sublingual immunotherapy (317) Allergy 2010 7 Suppl 92 133

[B307] Kerkvliet E Peekel I van Tuyn J Sleijster H van den Hout R Amount of major allergen Bet v 1 and allergen activity in different European products for sublingual immunotherapy Allergy 2011 7 Suppl 94 622 623

[B308] Pfaar O Klimek L Subcutaneous and sublingual immunotherapy [in German] CME 2010 7 doi:10.1007/s11298-11010-10657-11295

[B309] Pfaar O Klimek L [Specific immunotherapy for allergic rhinitis. Current methods and innovative developments] HNO 2008 7 764 775 10.1007/s00106-008-1732-z 18668217

[B310] Osterberg L Blaschke T Adherence to medication N Engl J Med 2005 7 487 497 10.1056/NEJMra050100 16079372

[B311] Lombardi C Gani F Landi M Falagiani P Bruno M Canonica GW Passalacqua G Quantitative assessment of the adherence to sublingual immunotherapy J Allergy Clin Immunol 2004 7 1219 1220 10.1016/j.jaci.2004.03.013 15214362

[B312] Marogna M Spadolini I Massolo A Canonica GW Passalacqua G Randomized controlled open study of sublingual immunotherapy for respiratory allergy in real-life: clinical efficacy and more Allergy 2004 7 1205 1210 10.1111/j.1398-9995.2004.00508.x 15461603

[B313] Passalacqua G Musarra A Pecora S Amoroso S Antonicelli L Cadario G Di Gioacchino M Lombardi C Ridolo E Sacerdoti G Schiavino D Senna G Quantitative assessment of the compliance with a once-daily sublingual immunotherapy regimen in real life (EASY Project: Evaluation of A novel SLIT formulation during a Year) J Allergy Clin Immunol 2006 7 946 948 10.1016/j.jaci.2005.12.1312 16630956

[B314] Passalacqua G Musarra A Pecora S Amoroso S Antonicelli L Cadario G Di Gioacchino M Lombardi C Ridolo E Sacerdoti G Schiavino D Senna G Quantitative assessment of the compliance with once-daily sublingual immunotherapy in children (EASY project: evaluation of a novel SLIT formulation during a year) Pediatr Allergy Immunol 2007 7 58 62 10.1111/j.1399-3038.2006.00471.x 17295800

[B315] Röder E Berger MY de Groot H Gerth van Wijk R Sublingual immunotherapy in youngsters: adherence in a randomized clinical trial Clin Exp Allergy 2008 7 1659 1667 10.1111/j.1365-2222.2008.03060.x 18631346

[B316] Egert-Schmidt A Martin E Mueller J Schulte M Thum-Oltmer S Patients’ compliance towards high-dose hypoallergenic and unmodified SCIT treatment schedules is superior to sublingual immunotherapy [abstract 1669] Allergy 2011 7 628 629

[B317] Kiel MA Roder E Gerth van Wijk R Al MJ Hop WC Rutten-van Molken MP Real-life compliance and persistence among users of subcutaneous and sublingual allergen immunotherapy J Allergy Clin Immunol 2013 7 353 360 e352 10.1016/j.jaci.2013.03.013 23651609

[B318] Incorvaia C Rapetti A Scurati S Puccinelli P Capecce M Frati F Importance of patient’s education in favouring compliance with sublingual immunotherapy Allergy 2010 7 1341 1342 10.1111/j.1398-9995.2010.02347.x 20192941

[B319] Jansen A Andersen KF Bruning H Evaluation of a compliance device in a subgroup of adult patients receiving specific immunotherapy with grass allergen tablets (GRAZAX) in a randomized, open-label, controlled study: an a priori subgroup analysis Clin Ther 2009 7 321 327 10.1016/j.clinthera.2009.02.005 19302904

[B320] Senna G Caminati M Canonica GW Safety and tolerability of sublingual immunotherapy in clinical trials and real life Curr Opin Allergy Clin Immunol 2013 7 656 662 10.1097/ACI.0000000000000007 24126613

[B321] Valenta R Campana R Marth K van Hage M Allergen-specific immunotherapy: from therapeutic vaccines to prophylactic approaches J Intern Med 2012 7 144 157 10.1111/j.1365-2796.2012.02556.x 22640224 PMC4573524

[B322] Cromwell O Hafner D Nandy A Recombinant allergens for specific immunotherapy J Allergy Clin Immunol 2011 7 865 872 10.1016/j.jaci.2011.01.047 21377719

[B323] Braido F Holgate S Canonica GW From “blockbusters” to “biosimilars”: an opportunity for patients, medical specialists and health care providers Pulm Pharmacol Ther 2012 7 483 486 10.1016/j.pupt.2012.09.005 23010202

[B324] Allam JP Peng WM Appel T Wenghoefer M Niederhagen B Bieber T Berge S Novak N Toll-like receptor 4 ligation enforces tolerogenic properties of oral mucosal Langerhans cells J Allergy Clin Immunol 2008 7 368 374 e361 10.1016/j.jaci.2007.09.045 18036651

[B325] Moingeon P Lombardi V Saint-Lu N Tourdot S Bodo V Mascarell L Adjuvants and vector systems for allergy vaccines Immunol Allergy Clin North Am 2011 7 407 419 xii 10.1016/j.iac.2011.03.001 21530828

[B326] Smits HH Engering A van der Kleij D de Jong EC Schipper K van Capel TM Zaat BA Yazdanbakhsh M Wierenga EA van Kooyk Y Kapsenberg ML Selective probiotic bacteria induce IL-10-producing regulatory T cells in vitro by modulating dendritic cell function through dendritic cell-specific intercellular adhesion molecule 3-grabbing nonintegrin J Allergy Clin Immunol 2005 7 1260 1267 10.1016/j.jaci.2005.03.036 15940144

[B327] Pizza M Giuliani MM Fontana MR Monaci E Douce G Dougan G Mills KH Rappuoli R Del Giudice G Mucosal vaccines: non toxic derivatives of LT and CT as mucosal adjuvants Vaccine 2001 7 2534 2541 10.1016/S0264-410X(00)00553-3 11257389

[B328] Saint-Lu N Tourdot S Razafindratsita A Mascarell L Berjont N Chabre H Louise A Van Overtvelt L Moingeon P Targeting the allergen to oral dendritic cells with mucoadhesive chitosan particles enhances tolerance induction Allergy 2009 7 1003 1013 10.1111/j.1398-9995.2009.01945.x 19220212

[B329] Uddowla S Freytag LC Clements JD Effect of adjuvants and route of immunizations on the immune response to recombinant plague antigens Vaccine 2007 7 7984 7993 10.1016/j.vaccine.2007.09.030 17933440 PMC2443708

[B330] Mistrello G Brenna O Roncarolo D Zanoni D Gentili M Falagiani P Monomeric chemically modified allergens: immunologic and physicochemical characterization Allergy 1996 7 8 15 8721522 10.1111/j.1398-9995.1996.tb04543.x

[B331] Cosmi L Santarlasci V Angeli R Liotta F Maggi L Frosali F Rossi O Falagiani P Riva G Romagnani S Annunziato F Maggi E Sublingual immunotherapy with dermatophagoides monomeric allergoid down-regulates allergen-specific immunoglobulin E and increases both interferon-gamma- and interleukin-10-production Clin Exp Allergy 2006 7 261 272 10.1111/j.1365-2222.2006.02429.x 16499636

[B332] Passalacqua G Albano M Fregonese L Riccio A Pronzato C Mela GS Canonica GW Randomised controlled trial of local allergoid immunotherapy on allergic inflammation in mite-induced rhinoconjunctivitis Lancet 1998 7 629 632 10.1016/S0140-6736(97)07055-4 9500318

[B333] Bagnasco M Mariani G Passalacqua G Motta C Bartolomei M Falagiani P Mistrello G Canonica GW Absorption and distribution kinetics of the major Parietaria judaica allergen (Par j 1) administered by noninjectable routes in healthy human beings J Allergy Clin Immunol 1997 7 122 129 10.1016/S0091-6749(97)70203-3 9257796

[B334] Burks AW Calderon MA Casale T Cox L Demoly P Jutel M Nelson H Akdis CA Update on allergy immunotherapy: American Academy of Allergy, Asthma & Immunology/European Academy of Allergy and Clinical Immunology/PRACTALL consensus report J Allergy Clin Immunol 2013 7 1288 1296 e1283 10.1016/j.jaci.2013.01.049 23498595

[B335] Penagos M Compalati E Tarantini F Baena-Cagnani R Huerta J Passalacqua G Canonica GW Efficacy of sublingual immunotherapy in the treatment of allergic rhinitis in pediatric patients 3 to 18 years of age: a meta-analysis of randomized, placebo-controlled, double-blind trials Ann Allergy Asthma Immunol 2006 7 141 148 10.1016/S1081-1206(10)60004-X 16937742

[B336] Penagos M Passalacqua G Compalati E Baena-Cagnani CE Orozco S Pedroza A Canonica GW Metaanalysis of the efficacy of sublingual immunotherapy in the treatment of allergic asthma in pediatric patients, 3 to 18 years of age Chest 2008 7 599 609 10.1378/chest.06-1425 17951626

[B337] Bachert C Canonica GW Bufe A SIT: efficacy depends on product, not on route of application Pediatr Allergy Immunol 2012 7 401 author reply 401-402 22594932 10.1111/j.1399-3038.2012.01285.x

[B338] Sade K Berkun Y Dolev Z Shalit M Kivity S Knowledge and expectations of patients receiving aeroallergen immunotherapy Ann Allergy Asthma Immunol 2003 7 444 448 10.1016/S1081-1206(10)61511-6 14692426

[B339] Baiardini I Puggioni F Menoni S Boot JD Diamant Z Braido F Canonica GW Patient knowledge, perceptions, expectations and satisfaction on allergen-specific immunotherapy: a survey Respir Med 2013 7 361 367 10.1016/j.rmed.2012.11.004 23218454

[B340] Calderon MA Demoly P Gerth Van Wijk R Bousquet J Sheikh A Frew A Scadding G Bachert C Malling HJ Valenta R Bilo B Nieto A Akdis C Just J Vidal C Varga EM Alvarez-Cuesta E Bohle B Bufe A Canonica WG Cardona V Dahl R Didier A Durham SR Eng P Fernandez-Rivas M Jacobsen L Jutel M Kleine-Tebbe J Klimek L EAACI: A European Declaration on Immunotherapy. Designing the future of allergen specific immunotherapy Clin Transl Allergy 2012 7 20 10.1186/2045-7022-2-20 23110958 PMC3514324

[B341] Pawankar R, Canonica GW, Holgate ST, Lockey RF World Allergy Organization (WAO) White Book on Allergy 2011 Milwaukee, WI: WAO

